# Comparison of Photophysical Properties of Lanthanide(III) Complexes of DTTA- or DO3A-Appended Aryl-2,2′-Bipyridines

**DOI:** 10.3390/molecules28020724

**Published:** 2023-01-11

**Authors:** Alexey P. Krinochkin, Maria I. Valieva, Ekaterina S. Starnovskaya, Yaroslav K. Shtaitz, Dmitry S. Kopchuk, Olga S. Taniya, Grigory A. Kim, Vadim A. Shevyrin, Grigory V. Zyryanov, Oleg N. Chupakhin

**Affiliations:** 1 Chemical Engineering Institute, Ural Federal University, 19 Mira St., Yekaterinburg 620002, Russia; 2 I. Ya. Postovsky Institute of Organic Synthesis of RAS, Ural Division, 22/20 S. Kovalevskoy/Akademicheskaya St., Yekaterinburg 62099, Russia

**Keywords:** DO3A, DTTA, 2,2′-Bipyridines, Eu(III) complex, Tb(III) complex, lanthanide(III) cation luminescence, comparison of photophysical properties

## Abstract

New Tb(III) and Eu(III) complexes based on aryl-2,2′-bipyridine ligands with a cyclic DO3A chelating unit appended in the alpha position of the bipyridine core were synthesized. The photophysical properties of these complexes were compared with those of complexes of ligands with identical aryl-2,2′-bipyridine chromophores, but with an acyclic DTTA residue as an additional chelating site in the alpha position of the bipyridine core. The nature of the polyaminocarboxylic acid fragments was found to have a significant influence on the luminescence. For some of the Eu(III) complexes, upon the transition from acyclic DTTA- to the cyclic DO3A-appended ligands, a noticeable increase in the intensity of Eu(III) luminescence was observed, with an increase in the quantum yield of up to 2.55 times. In contrast, for most of the Tb(III) complexes, a similar transition resulted in a noticeable decrease in the luminescence intensity of the Tb(III) cation.

## 1. Introduction

Water-soluble luminescent complexes of lanthanide(III) cations are of considerable interest [1,2] for applications such as chromophores for phosphorescent labels [3], sensors for metal cations [4,5,6], and singlet oxygen sensors [7].

The ligands associated with these complexes have chromophore parts in their structure that are necessary for absorbing light, in order to transfer energy to the metal cation [8]. The chelating part also needs to be of rigid character to enable strong binding of the lanthanide cation. It should be noted that the presence of a water molecule in the coordination sphere of the lanthanide cation leads to quenching of luminescence due to the nonradiative relaxation of its excited state through energy transfer to the high-frequency oscillating O–H bonds of water molecules. To prevent this, complete filling of the coordination sphere of the lanthanide cation is required.

A number of lanthanide complexes of various 2,2′-bipyridines bearing polyaminocarboxylic acid fragments in the alpha position have been described in the literature. For these complexes, there is a requirement that saturation of all coordination bonds of the lanthanide cation is met, and this has been repeatedly confirmed by calculations based on the values of the luminescence lifetime in water and heavy water (D_2_O). To create these complexes, chromophores such as unsubstituted 2,2′-bipyridine [9], 7-*tert*-butyl-2-(2-pyridyl)-5*H*-chromeno [2,3-*b*]pyridin-5-one [10], 9-methyl-1,10-phenanthroline [11,12], and its 4,7-diphenyl derivative [13], as well as 10,11,12,13-tetrahydrodipyrido [3,2-*a*:2′,3′-*c*]phenazine [10] and various aryl-containing 2,2′-bipyridines [14,15,16,17,18,19], have been used.

In this study, groups such as cyclic DO3A or acyclic DTTA were used as the polyaminocarboxylic acid fragments. However, there are very few reports of the preparation of complexes with a similar chromophore and residues of various polyaminocarboxylic acids in the literature. In one example, the photophysical properties of pairs of luminescent lanthanide complexes with a chromophore based on 5-phenyl-2,2′-bipyridine [19] and 9-methyl-1,10-phenanthroline [11,12] were previously published, and a slight decrease in the quantum yield of the luminescence of europium(III) and terbium(III) cations upon passing from the DTTA to the DO3A residue was observed. In this work, for the first time, we have carried out a broader analysis of the influence of the nature of the polyaminocarboxylic acid, such as being cyclic (DO3A) or acyclic (DTTA), on the photophysical properties of the lanthanide(III) chelates obtained, comparing a number of different aryl-containing 2,2′-bipyridines as the ligands/chromophores.

## 2. Results

### 2.1. Synthesis of the New Water-Soluble Lanthanide(III) Complexes

**Scheme 1:**
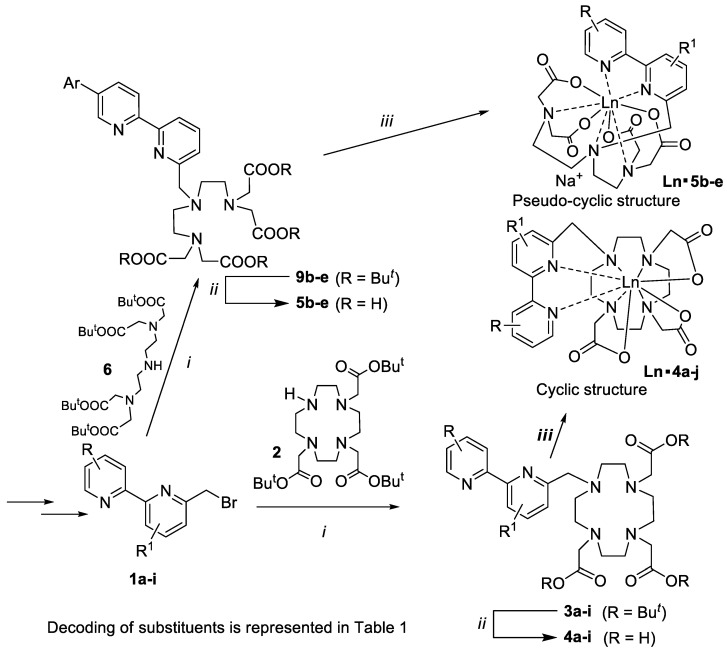
Synthesis of lanthanide complexes **Ln**•**4** and **Ln**•**5.** *Reagents and conditions*: (*i*) K_2_CO_3_/CH_3_CN, 82 °C, 16 h; (*ii*) HCl (5N), rt, overnight, then HCl (11 N), rt, 2 h, then acetonitrile, rt, overnight; (*iii*) NaOH, water, rt, 5 min, then LnCl_3_•6H_2_O, rt.

The synthesis of the target ligands and their complexes is shown in Scheme 1. The corresponding alpha-bromomethylbipyridines (shown at **1** in Scheme 1) were used as key intermediates. Further alkylation of the commercially available tris-*tert*-butyl ester of DO3A (**2**), carried out according to the previously reported procedure [19], made it possible to obtain the precursors of the ligands shown at **3**. The structure of the compounds at **3** was confirmed through ^1^H NMR and mass (electrospray) spectra. Thus, in the ^1^H NMR spectra, signals from the protons of the arylbipyridine system, as well as signals from the DO3A fragment in the resonance region of the aliphatic protons, were observed. Separately, the signals from the protons of the methylene moiety, which connects the corresponding 2,2′-bipyridine fragment and the DO3A moiety, were observed in the region of 3.83–3.89 ppm. In this case, the change in the chemical shifts of these protons compared to those of the bromomethyl-substituted 2,2′-bipyridines shown at **1** was quite informative.

The subsequent removal of *tert*-butyl protection in hydrochloric acid resulted in the ligands shown at **4** manifesting as hydrochlorides; their composition was established on the basis of elemental analysis data. Finally, the interaction of the trisodium salts of the DTTA- or DO3A-based ligands (obtained in situ) with the lanthanide chlorides led to the target **Ln•4** complexes.

**Scheme 2:**
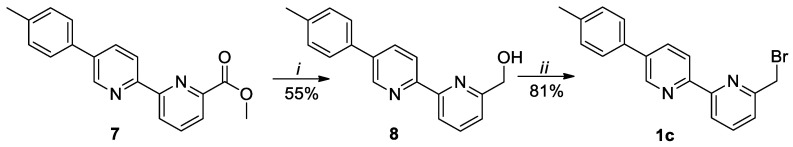
Synthesis of 6-bromomethylbipyridine (**1c**). Reagents and conditions: (*i*) NaBH_4_/EtOH, 78 °C, 4 h; (*ii*) PBr_3_/CH_2_Cl_2_, 50 °C, 2 h.

Most of the complexes of the DTTA-appended ligands studied in this work have been described previously. However, some of them were synthesized for the first time; in particular, the complexes based on 5-aryl-2,2′-bipyridines. Thus, ligands **5d** and **5e** have been described by us earlier [20], and the synthesis of ligand **5b** was performed on the basis of the corresponding 6-bromomethylbipyridine (**1b**) as described earlier [19], as a result of alkylation of the DTTA ester shown at **6** [21,22]. In the case of the 4-tolyl-containing ligand (**5c**), the 5-tolyl-2,2′-bipyridine-6-carboxylic acid methyl ester (**7**) reported above was used as a starting compound [23] (Scheme 2).

**Figure 1:**
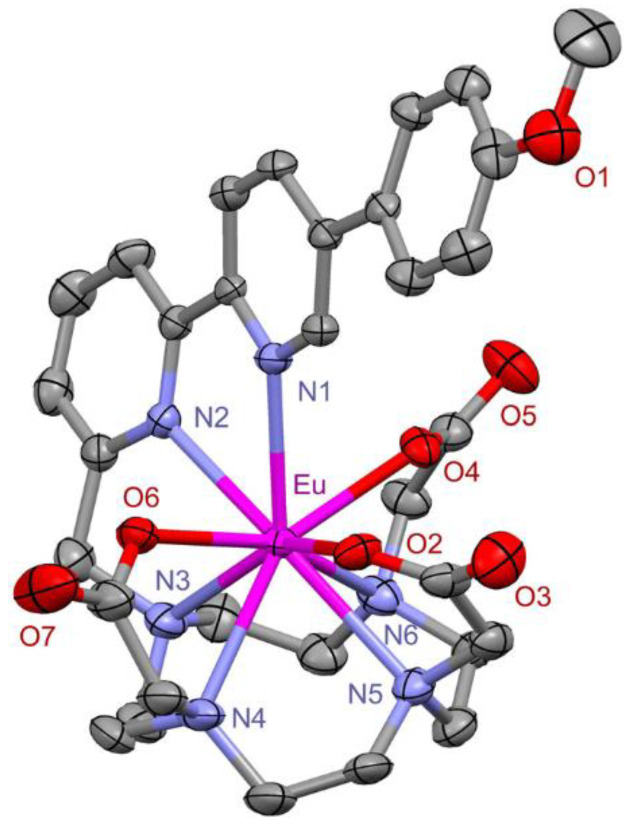
X-Ray diffraction data for the **Eu•4b** complex [19]. Reprinted with the permission of [19]. Copyright © 2011 Elsevier B.V.

The compositions of the new complexes **Ln•4** and **Ln•5** were confirmed on the basis of mass spectrometry (electrospray) and elemental analysis results. It should be noted that the structure of the Eu(III) complex **Eu•4b** bearing 5-(4-methoxyphenyl)-2,2′-bipyridine as a chromophore was previously confirmed by us by means of X-ray diffraction analysis [19] (Figure 1). Thus, the simultaneous participation of the 2,2′-bipyridine moiety and the DO3A fragment in the chelation of the europium(III) cation was confirmed.

### 2.2. Photophysical Properties of the New Water-Soluble Lanthanide(III) Complexes

The results of the photophysical studies of the new complexes, as well as some earlier data, are summarized in Table 1. The quantum yield of the previously described **Tb•5f** complex was measured using standards considered to be more suitable than the quinine sulfate which was used previously [14], such as ([Ru(bpy)_3_]Cl_2_).

**Figure 2:**
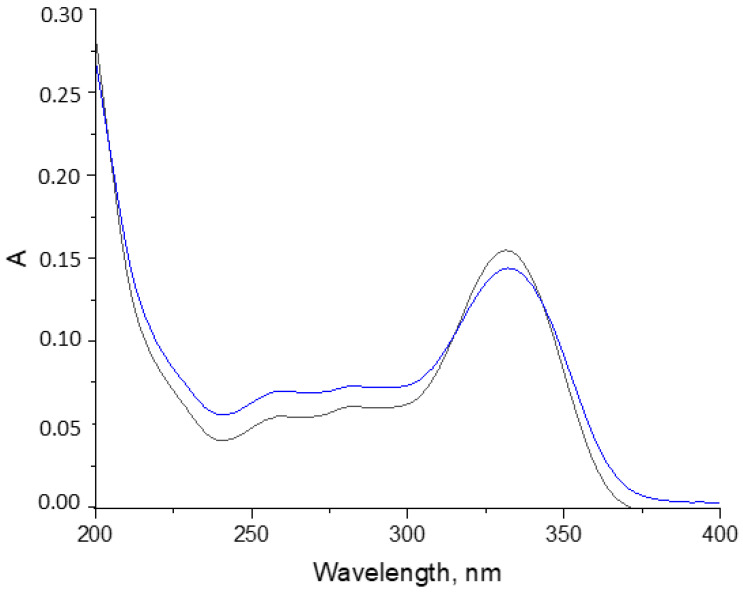
Absorption spectra of complexes **Eu•5c** (black line) and **Eu•4c** (blue line) in water at room temperature.

**Figure 3:**
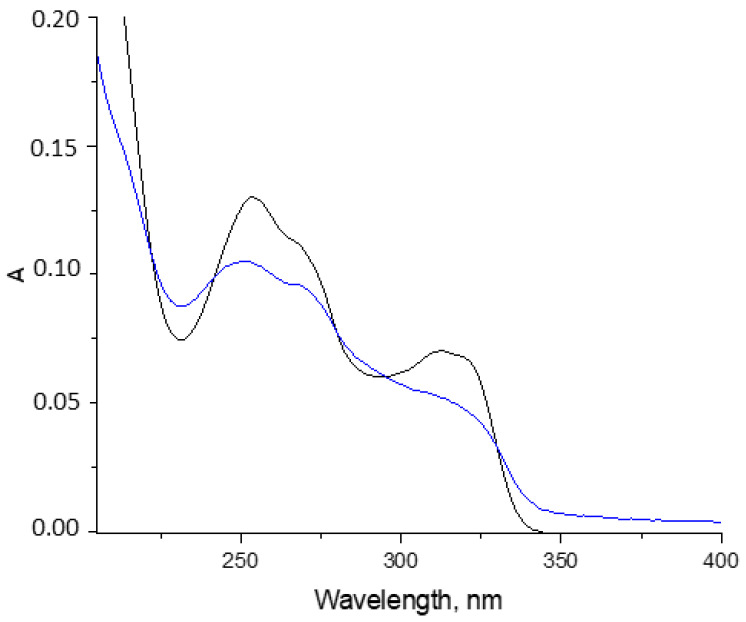
Absorption spectra of complexes **Tb•5g** (black line) and **Tb•4g** (blue line) in water at room temperature.

In regard to the confirmation of the participation of the 2,2′-bipyridine fragment in the chelation of the lanthanide (III) cations in the new complexes, we draw attention to the previously obtained europium complex **Eu•10** based on 5-phenyl-2,2′-bipyridine with a DTTA moiety at the C5′ position (Table 1) [21]. In this case, the chelation of the europium(III) cation by the bipyridine fragment is impossible. Analysis of the absorption maxima of the corresponding complexes **Eu•10** and **Eu•5a** with the same chromophore demonstrated a noticeable bathochromic shift for the complex **Eu•5a** compared to chelate **Eu•10** (327 nm vs. 304 nm). The absorption maxima in water for the corresponding complexes **Ln•4** and **Ln•5** with the same chromophore either differed by several nm or were the same. Taking into account the data from the XRD analysis of the complex **Ln•4b** [19] and these data, we may assume the involvement of the 2,2′-bipyridine fragment in the chelation of lanthanide(III) cations for the complexes termed **Ln•5**. The absorption spectra of the pairs of complexes **Eu•5c**/**Eu•5c** and **Tb•5g**/**Tb•4g** are shown in Figure 2 and Figure 3.

Only for the complexes **Ln•4j** and **Tb•5j** with 1,10-phenanthroline as the chromophore was there a noticable difference between the absorption maxima (16 nm).

Moreover, the calculations based on the values of the luminescence lifetime in water and D_2_O for the complex **Eu•10** showed the presence of two water molecules in the first coordination sphere of the europium(III) cation. This fact can be explained by the absence of the two coordination bonds with the nitrogen atoms of the 2,2′-bipyridine moiety [21]. For all the new complexes termed as **Ln•4** and **Ln•5**, similar calculations showed the absence of any water molecules in the first coordination sphere of the lanthanide cation. Thus, all these data confirmed the structures of the complexes **Ln•4** and **Ln•5**.

**Figure 4:**
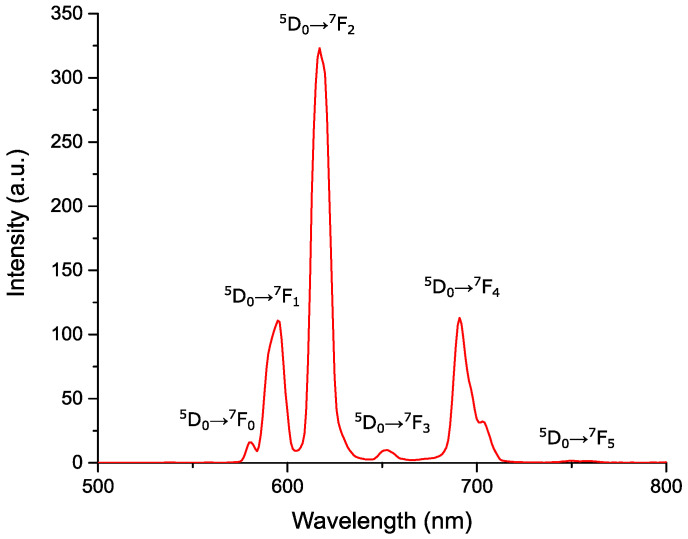
Europium cation luminescence spectrum of complex **Eu•4g** in water at room temperature (excitation at 317 nm).

**Figure 5:**
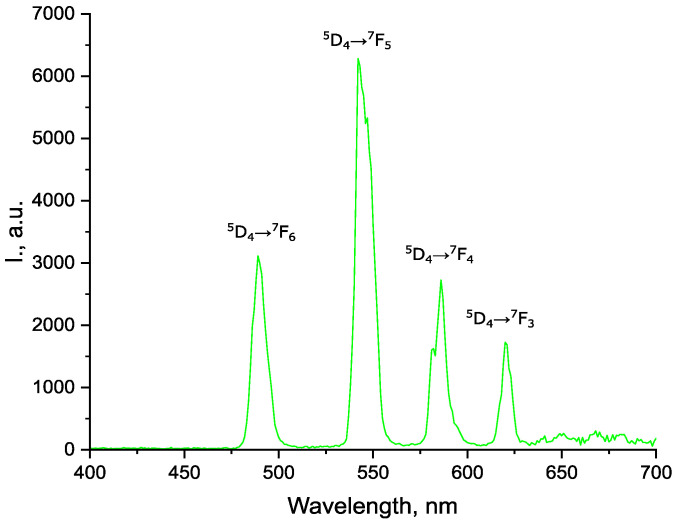
Terbium cation luminescence spectrum of complex **Tb•4g** in water at room temperature (excitation at 317 nm).

Upon photoexcitation, all the new complexes exhibited a pronounced characteristic luminescence of the corresponding lanthanide(III) cation. Examples of the luminescence spectra with attributions of the emission peaks are represented in Figure 4 and Figure 5. The luminescence emission spectra and the decay curves for all the new complexes termed **Ln•4** and **Ln•5** are presented in the Supplementary Materials.

As mentioned above, in the case of previously published complexes, there was no significant difference in the luminescence intensity of the terbium(III) and europium(III) cations, regardless of the nature of the additional chelating site (DTTA or DO3A). There was only a slight decrease in the quantum yields between the acyclic DTTA and the cyclic DO3A fragment. Based on these results, a conclusion could be made that the nature of the additional chelating site has an insignificant influence on the photophysical characteristics of the complexes.

**Figure 6:**
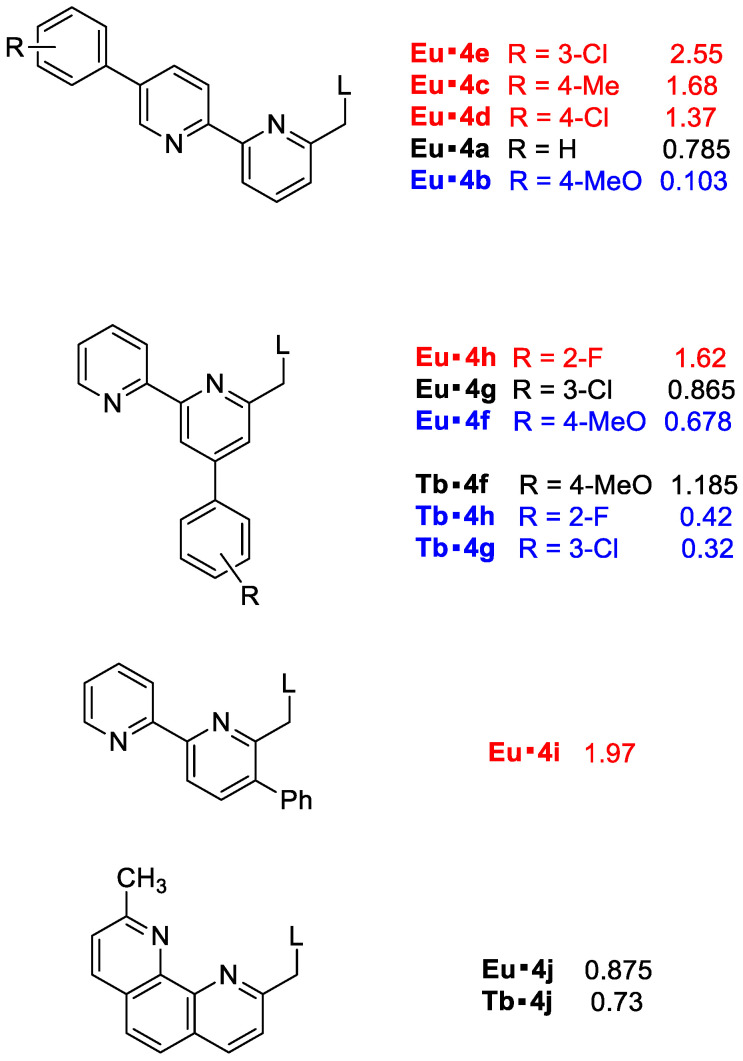
Ratios of quantum yields of lanthanide(III) cation of the corresponding complexes based on DO3A- and DTTA-appended ligands.

However, all of the data collected by us are in strong contradiction to this assumption, since, in a number of cases, there is a significant difference between the values of the lanthanide (III) cation luminescence quantum yields for the corresponding **Ln•4** and **Ln•5** complexes. The relationship between the quantum yields of the complexes with either a cyclic (DO3A) or acyclic (DTTA) chelating part is shown in Figure 6, and the complexes are listed in decreasing order of this indicator in the corresponding series. Where there was a significant increase in the intensity of the lanthanide cation luminescence upon the transition to the DO3A-appended ligand, it is highlighted in red. The cases with a significant decrease in the intensity of the lanthanide (III) cation luminescence are highlighted in blue.

Thus, in a number of the europium(III) complexes, there was a significant increase in the luminescence of the europium(III) cation. Moreover, within the series studied, a record growth in the luminescence quantum yield was observed for the **Eu•4e** complex based on 5-(3-chlorophenyl)-2,2′-bipyridine. A somewhat smaller growth took place for the closest analogs of **Eu•4e** bearing 4-tolyl and 4-chlorophenyl substituents, such as **Eu•4c** and **Eu•4d**. For phenyl substituents, as previously described [19], no significant difference between the luminescence intensities of the **Eu•4a** and **Eu•5a** complexes was observed. An unexpectedly sharp drop in all the photophysical properties, including the luminescence lifetime in water and D_2_O, was observed for the complex **Eu•4b** based on the 4-methoxyphenyl-containing ligand. In the case of 4-aryl-2,2′-bipyridine ligands, a significant increase in the luminescence intensity was observed for the **Eu•4h** complex based on a 2-fluorophenyl-containing ligand (the ratio was 1.62). For the other two complexes, **Eu•4g** and **Eu•4f**, a decreased luminescence of the europium (III) cation was observed. In this regard, this effect seen in the complex of the 4-methoxyphenyl-containing ligand **Eu•4f** correlated well with the data for the **Eu•4b** complex, although the decrease in the luminescence intensity was not as significant as it was for the latter (the ratio was 0.678 instead of 0.103). A significant increase in the value of the quantum yield was also shown by the **Eu•4i** complex of the ligand based on 5-phenyl-2,2′-bipyridine with an additional chelating fragment in the C6 position (in the series studied, the observed ratio of 1.97 was the second highest result after that for the **Eu•4e** complex). When 1,10-phenanthroline was used as a chromophore, no significant changes in the luminescence intensity were observed upon passing to the DO3A-based complex (chelates **Eu•4j** and **Eu•5j**).

We also studied the photophysical properties for the corresponding terbium(III) complexes of 4-aryl-2,2′-bipyridine chromophores, but further series could not be studied due to a lack of satisfactory sensitization of the terbium(III) cation by the other ligands under consideration, namely, those based on 5-aryl-2,2′-bipyridine. According to the data obtained, there was an absence of a noticeable increase in the luminescence intensity of the terbium(III) cation in case of DO3A-appended ligands. Only for the 4-methoxyphenyl-substituted ligand (complex **Tb•4f**) was there a slight increase in the luminescence quantum yield of the terbium(III) cation, where it was higher by a factor of 1.185. In two other cases, the 2-fluorophenyl-substituted ligand (complex **Tb•4h**) and the 3-chlorophenyl-substituted ligand (complex **Tb•4g**), a noticeable decrease in the quantum yield was observed. It is worth mentioning that the ratios of the luminescence quantum yield of the corresponding DTTA-containing Tb(III) complexes were 0.42 and 0.32, respectively. The same trend was observed for the Tb(III) complexes with 2-methyl-1,10-phenanthroline chromophore, such as in complexes **Tb•4j** and **Tb•5j**, and the observed ratio here was 0.73. The **Tb•5i** complex exhibited low terbium (III) luminescence intensity, with a quantum yield as low as 0.43%, while for the similar **Tb•4i** complex based on a DO3A-appended ligand, a decreased terbium luminescence was observed, although we were unable to reliably measure its quantum yield.

An analysis of the luminescence lifetimes in water and D_2_O for the pairs of **Eu•4** and **Eu•5** complexes under consideration showed that, for a number of them, when moving to DO3A-appended ligands, there is an increase in the luminescence lifetime in water and a decrease in D_2_O. In particular, this behaviour was observed for the complexes based on ligands **4a**/**5a**, **4d**/**5d**, and **4e**/**5e**.

## 3. Experimental Methods

### Materials and Equipment

All reagents were purchased from commercial sources and used without further purification. Silica gel 60 (Kieselgel 60, 230–400 mesh) was used for the column chromatography. NMR spectra were recorded on a Bruker Avance-400 (or Bruker Avance-500) spectrometer, 298 K, digital resolution ± 0.01 ppm, using TMS as internal standard. UV-Vis spectra were recorded on UV-2600 spectrophotometer (Shimadzu). Luminescence spectra were recorded on a FS-5 spectrofluorometer (Edinburgh Instruments); luminescence spectra were corrected using Edinburgh correction curves. Mass spectra were recorded on a MicrOTOF-Q II mass spectrometer (Bruker Daltonics) with electrospray ionization. Elemental analyses were performed using a PE 2400 II CHN-analyzer (Perkin Elmer). The chlorine content needed for the elemental analysis of ligands was measured using the mercurimetry method. Compounds **1b** [19], **1d-e** [20], **1f** [14], **1g** [15], **1h** [16], **1i** [18], **5d-e** [20], DTTA ester **6** [21,22], and **7** [23] were synthesized as described in the literature.

Detailed experimental procedures and characterizations of the products are presented in the Supplementary Materials.

## 4. Conclusions

In conclusion, for the first time, a broad comparison of the properties of Eu^3+^ and Tb^3+^ complexes based on ligands with the same aryl-2,2′-bipyridine series chromophores but having core additional chelating sites in the alpha position of the 2,2′-bipyridines, such as non-cyclic DTTA or cyclic DO3A residues, was carried out. In contrast to the previously published reports on the DTTA- and DO3A-based Ln(III) chelates, we found that the nature of the polyaminocarboxylic acid fragment had a significant influence on the Ln(III) luminescence in the resulting Ln(III) complex. For example, in the case of some Eu(III) complexes, passing from the DTTA- to the DO3A-appended ligands resulted in a noticeable increase in the intensity of the lanthanide(III) luminescence, with an increase in the quantum yield of up to 2.55 times. In contrast, for most of the DO3A-based Tb(III) complexes, a decrease in the luminescence intensity of the terbium(III) cation was observed. Most probably, upon the formation of macrocyclic Eu(III) complexes with DO3A-appended 2,2′-bipyridine ligands, a more efficient sensitization of the chelated Eu(III) cation takes place compared to the pseudo-cyclic Eu(III) complexes of the same ligands bearing a DTTA moiety.

## Figures and Tables

**Table 1:** Photophysical properties of new complexes of **Ln•4,5,10** and previously published similar complexes.

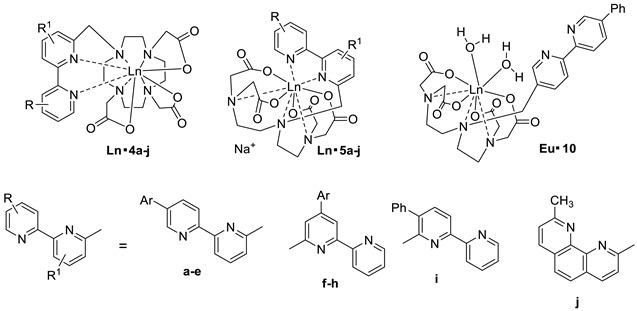									
Entry	Ligand, [Ref.]	Ar	Ln	λ_max_, nm ^a^	E ^b^, 10^−3^ M^−1^·cm^−1^	Φ_Ln_, % ^c^	τ_H2O_, ms ^d^	τ_D2O_, ms ^e^	q ^f^
**1**	**10 [21]**	Ph	Eu	304	7.95	0.34	0.369	1.734	2.02
2	**5a [19]**	Ph	Eu	278, 327	12.02	9.3	0.80	1.30	0.27
3	**4a [19]**	Ph	Eu	325	7.505	7.3	1.00	1.10	−0.19
4	**5b**	4-MeOC_6_H_4_	Eu	261, 339	12.1	10.7	0.95	1.53	0.18
5	**4b [19]**	4-MeOC_6_H_4_	Eu	341	9.5	1.1	0.27	0.30	0.15
6	**5c**	4-Tol	Eu	281, 331	11.5	14.8	1.10	1.90	0.16
7	**4c**	4-Tol	Eu	261, 282, 332	10.8	**24.9**	1.094	1.586	0.05
8	**5d**	4-ClC_6_H_4_	Eu	280, 328	11.64	**17.6**	1.106	1.936	0.17
9	**4d**	4-ClC_6_H_4_	Eu	258_sh_, 280, 331	12.87	24.1	1.20	1.67	−0.02
10	**5e**	3-ClC_6_H_4_	Eu	274, 325	11.72	12.2	1.08	1.927	0.19
11	**4e**	3-ClC_6_H_4_	Eu	255_sh_, 273, 326	11.02	31.1	1.14	1.58	0
12	**5f [14]**	4-MeOC_6_H_4_	Eu	260_sh_, 278, 311	9.2	23.0	0.98	1.02	−0.25
13	**4f**	4-MeOC_6_H_4_	Eu	253, 279, 315	9.78	15.6	1.13	1.61	0.02
14	**5f**	4-MeOC_6_H_4_	Tb	261_sh_, 278, 311	9.3	11.3	0.35	0.37	0.26
15	**4f**	4-MeOC_6_H_4_	Tb	253, 278, 316	9.5	13.4	0.368	0.387	0.32
16	**5g [15]**	3-ClC_6_H_4_	Eu	254, 259_sh_, 265, 276_sh_, 314, 329_sh_	8.6	25.2	1.03	1.71	0.17
17	**4g**	3-ClC_6_H_4_	Eu	250, 269, 317_sh_	9.1	21.8	1.12	1.62	0.03
18	**5g [15]**	3-ClC_6_H_4_	Tb	254, 259_sh_, 265, 276_sh_, 313, 330_sh_	8.6	56.1	1.10	1.23	0.17
19	**4g**	3-ClC_6_H_4_	Tb	253, 268_sh_, 317_sh_	9.2	18.1	0.473	0.503	0.28
20	**5h [16]**	2-FC_6_H_4_	Eu	251, 267, 274_sh_, 312, 322_sh_	8.9	16.2	1.07	1.86	0.18
21	**4h**	2-FC_6_H_4_	Eu	251, 266_sh_, 314,321_sh_	9.5	26.2	1.13	1.66	0.04
22	**5h [16]**	2-FC_6_H_4_	Tb	251, 267, 274_sh_, 312, 322_sh_	9.2	49.2	0.99	1.27	0.68
23	**4h**	2-FC_6_H_4_	Tb	251, 267_sh_, 312, 322_sh_	9.9	20.9	0.527	0.567	0.32
24	**5i [18]**	Ph	Eu	254, 316	9.7	12.8	1.09	1.88	0.17
25	**4i**	Ph	Eu	253, 262_sh_, 316	9.6	25.2	1.16	1.70	0.03
26	**5i [18]**	Ph	Tb	254, 317	9.8	0.43	-	-	-
27	**4i**	Ph	Tb	254, 262_sh_, 317	10.2	<0.1	-	-	-
28	**5** **j [12]**	-	Eu	279	-	24	1.25	1.88	0.02
29	**4j [11]**	-	Eu	295	-	21	1.24	1.77	−0.01
30	**5j [12]**	-	Tb	279	-	15	0.78	0.82	0.01
31	**4j [11]**	-	Tb	295	-	11	0.31	0.32	0.2

^a^ Absorption maxima in water at room temperature; ^b^ Molar extinction coefficients correspond to the longest wavelength absorption maxima; ^c^ Lanthanide fluorescence quantum yield was determined relative to [Ru(bpy)_3_]Cl_2_ (Φ = 0.04 in aerated water) [24]; ^d^ Lanthanide fluorescence lifetime in water; ^e^ Lanthanide fluorescence lifetime in D_2_O; ^f^ The number of coordinated water molecules were calculated using the formulas: q_Eu_ = 1.2 ∗ (1/τ_H2O_ − 1/τ_D2O_ − 0.25) [25]; q_Tb_ = 4.2 ∗ (1/τ_H2O_ − 1/τ_D2O_ − 0.06) [26].

## Data Availability

The data are available on request from the corresponding authors.

## Supplementary Materials

The following supporting information can be downloaded at: https://www.mdpi.com/article/10.3390/molecules28020724/s1, Figures S1–S12: ^1^H and ^19^F NMR spectra of compounds **1c**, **3c-i**, **9b-c** and **8**; Figures S13–S27: mass-spectra of complexes **Ln•4** and **Ln•5**; Figures S28–S35: absorption spectra of complexes **Ln•4** and **Ln•5** in H_2_O; Figures S36–S49: luminescence spectrum and decay curves of complexes **Ln•4** and **Ln•5**.

## Sample Availability

Samples of the compounds **4** and **5** are available from the authors.
