# Supplementary material for: Comparison of Photophysical Properties of Lanthanide(III) Complexes of DTTA- or DO3A-Appended Aryl-2,2′-Bipyridines
Source: Molecules. 2023 Jan 11;28(2):724. doi: 10.3390/molecules28020724 (PMC9864760; doi:10.3390/molecules28020724)
Supplement: Supplementary file 1 [file molecules-28-00724-s001.zip › molecules-2101245-supplementary.pdf]

Comparison of photophysical properties of lanthanide(III) complexes of DTTA- or DO3A-appended aryl-2,2′-bipyridines

Alexey P. Krinochkin [1,2], Maria I. Valieva [1,2], Ekaterina S. Starnovskaya [1,2], Yaroslav K. Shtaitz [1], Dmitry S. Kopchuk [1,2], Olga S. Taniya [1,2], Grigory A. Kim [1,2], Vadim A. Shevyrin [1], Grigory V. Zyryanov [1,2]*, Oleg N. Chupakhin [1,2]

[1] Chemical Engineering Institute, Ural Federal University, 19 Mira St., Yekaterinburg 620002, Russia

[2] I. Ya. Postovsky Institute of Organic Synthesis of RAS, Ural Division, 22/20 S. Kovalevskoy/Akademicheskaya St., Yekaterinburg 620137, Russia

SUPPLEMENTARY MATERIALS

Contents

1.	Experimental procedures	S4
2.	Figure S1. ^{1}H NMR (400 MHz, DMSO-d_6) of **8**	S11
3.	Figure S2. ^{1}H NMR (400 MHz, DMSO-d_6) of **1c**	S12
4.	Figure S3. ^{1}H NMR (500 MHz, $CDCl_3$) of **3c**	S13
5.	Figure S4. ^{1}H NMR (500 MHz, $CDCl_3$) of **3d**	S14
6.	Figure S5. ^{1}H NMR (500 MHz, $CDCl_3$) of **3e**	S15
7.	Figure S6. ^{1}H NMR (500 MHz, $CDCl_3$) of **3f**	S16
8.	Figure S7. ^{1}H NMR (500 MHz, $CDCl_3$) of **3g**	S17
9.	Figure S8. ^{1}H NMR (500 MHz, $CDCl_3$) of **3h**	S18
10.	Figure S9. ^{19}F NMR (400 MHz, $CDCl_3$) of **3h**	S19
11.	Figure S10. ^{1}H NMR (500 MHz, $CDCl_3$) of **3i**	S20
12.	Figure S11. ^{1}H NMR (400 MHz, DMSO-d_6) of **9b**	S21
13.	Figure S12. ^{1}H NMR (400 MHz, DMSO-d_6) of **9c**	S22
14.	Figure S13. Mass-spectrum of **Eu•4c**	S23
15	Figure S14. Mass-spectrum of **Eu•4d**	S24
16	Figure S15. Mass-spectrum of **Eu•4e**	S25
17	Figure S16. Mass-spectrum of **Eu•4f**	S26
18	Figure S17. Mass-spectrum of **Tb•4f**	S27
19	Figure S18. Mass-spectrum of **Eu•4g**	S28
20	Figure S19. Mass-spectrum of **Tb•4g**	S29
21	Figure S20. Mass-spectrum of **Eu•4h**	S30
22	Figure S21. Mass-spectrum of **Tb•4h**	S31
23	Figure S22. Mass-spectrum of **Eu•4i**	S32
24	Figure S23. Mass-spectrum of **Tb•4i**	S33
25	Figure S24. Mass-spectrum of **Eu•5b**	S34
26	Figure S25. Mass-spectrum of **Eu•5c**	S35
27	Figure S26. Mass-spectrum of **Eu•5d**	S36
28	Figure S27. Mass-spectrum of **Eu•5e**	S37
29	Figure S28. Absorption spectra of **Eu•4c** and **Eu•4d** in H_2O	S38
30	Figure S29. Absorption spectra of **Eu•4e** and **Eu•4f** in H_2O	S39
31	Figure S30. Absorption spectra of **Tb•4f** and **Eu•4g** in H_2O	S40
32	Figure S31. Absorption spectra of **Tb•4g a**nd **Eu•4h** in H_2O	S41
33	Figure S32. Absorption spectra of **Tb•4h** and **Eu•4i** in H_2O	S42

34 Figure S33. Absorption spectra of **Tb•4i** and **Eu•5b** in H_2O S43
35 Figure S34. Absorption spectra of **Eu•5c** and **Eu•5d** in H_2O S44
36 Figure S35. Absorption spectrum of **Eu•5e** in H_2O S45
37 Figure S36. Luminescence spectrum and decay curves of **Eu•5b** S46
38 Figure S37. Luminescence spectrum and decay curves of **Eu•5c** S47
39 Figure S38. Luminescence spectrum and decay curves of **Eu•4c** S48
40 Figure S39. Luminescence spectrum and decay curves of **Eu•5d** S49
41 Figure S40. Luminescence spectrum and decay curves of **Eu•4d** S50
42 Figure S41. Luminescence spectrum and decay curves of **Eu•5e** S51
43 Figure S42. Luminescence spectrum and decay curves of **Eu•4e** S52
44 Figure S43. Luminescence spectrum and decay curves of **Eu•4f** S53
45 Figure S44. Luminescence spectrum and decay curves of **Tb•4f** S54
46 Figure S45. Luminescence spectrum and decay curves of **Eu•4g** S55
47 Figure S46. Luminescence spectrum and decay curve of **Tb•4g** S56
48 Figure S47. Luminescence spectrum and decay curves of **Eu•4h** S57
49 Figure S48. Luminescence spectrum and decay curves of **Tb•4h** S58
50 Figure S49. Luminescence spectrum and decay curves of **Eu•4i** S59

1. Experimental procedures

(5′-*p*-Tolyl-2,2′-bipyridin-6-yl)methanol (8). The 6′-methoxycarbonyl-5-tolyl-2,2′-bipyridine **7** (365 mg, 1.20 mmol) was suspended in ethanol (100 ml), $NaBH_4$ (136 mg, 3.60 mmol) and the mixture was refluxed during 2 h. An additional portion of $NaBH_4$ (68 mg, 1.80 mmol) was added and the mixture was refluxed for another 2 h. Water (200 ml) was added and the product was extracted with methylene chloride (3×50 ml). The extract was dried on anhydrous sodium sulfate, and the solvent was removed in vacuum to give a product, which was used for next step without further purification. Yield 182 mg (0.66 mmol, 55%). **NMR ^{1}H** (DMSO-d_6, δ, ppm): 2.41 (s, 3H, CH_3), 4.66 (d, 2H, ^3J 4.8 Hz, CH_2), 5.17 (t, 1H, ^3J 4.8 Hz, OH), 7.28 (m, 2H, $C_6\underline{H}_4CH_3$), 7.50 (m, 1H, H-5), 7.60 (m, 2H, $C_6\underline{H}_4CH_3$), 7.86 (dd, 1H, ^3J 7.6, 7.6 Hz, H-4), 8.05 (dd, 1H, ^3J 8.2 Hz, ^4J 2.4 Hz, H-4′), 8.27 (m, 1H, H-3), 8.47 (d, 1H, ^3J 8.2 Hz, H-3′), 8.85 (d, 1H, ^4J 2.4 Hz, H-6′). **ESI-MS**, *m/z*: 277.13 $(M+H)^+$.

6′-(Bromomethyl)-5-*p*-tolyl-2,2′-bipyridine (1c). Hydroxymethylbipyridine **8** (152 mg, 0.55 mmol) was dissolved in 1,2-dichloroethane (35 ml). Then PBr_3 (0.10 ml, 1.1 mmol) was added to that solution and the mixture was stirred at 50 °C for 2 h. The resulting mixture was washed with aqueous solution of Na_2CO_3. The organic layer was dried over anhydrous sodium sulfate. The solvent was removed under reduced pressure. The analytical sample was obtained by recrystallization (ethanol). Yield 152 mg (0.45 mmol, 81%). **NMR ^{1}H** (DMSO-d_6, δ, ppm): 2.42 (s, 3H, CH_3), 4.69 (s, 2H, CH_2), 7.29 (m, 2H, $C_6\underline{H}_4CH_3$), 7.54 (m, 1H, H-5′), 7.59 (m, 2H, $C_6\underline{H}_4CH_3$), 7.89 (dd, 1H, ^3J 7.8, 7.8 Hz, H-4′), 8.06 (dd, 1H, ^3J 8.2 Hz, ^4J 2.4 Hz, H-4), 8.37 (m, 1H, H-3′), 8.50 (d, 1H, ^3J 8.2 Hz, H-3), 8.85 (d, 1H, ^4J 2.4 Hz, H-6). **ESI-MS**, *m/z*: 339.05 $(M+H)^+$. Found, %: C 63.55, H 4.30, N 8.38. $\mathbf{C_{18}H_{15}BrN_2}$. Calculated, %: C 63.73, H 4.46, N 8.26.

*General method for the obtaining of esters **3c-i***

The tri-*tert*-butyl ester **2** (DO3A) (206 mg, 0.40 mmol) and corresponding bromomethylbipyridine **1** (0.40 mmol) were dissolved in dry acetonitrile (40 ml), then potassium carbonate (276 mg, 2.00 mmol) was added and the resulting mixture was stirred under reflux for 2 days in argon atmosphere. Then solid was filtered off and washed with MeCN. Combined filtrates were evaporated under reduced pressure to dryness to give brown oil of crude product. It was dissolved in boiling hexane (40 mL), insoluble impurities were filtered off. Hexane solution was evaporated to dryness to give pure product as yellow oil.

***tert*-Butyl 2,2′,2″-(10-((5′-*p*-tolyl-2,2′-bipyridin-6-yl)methyl)-1,4,7,10-tetraaza-cyclododecane-1,4,7-triyl)triacetate (3c).** Yield 201 mg (0.26 mmol, 65%). **NMR ^{1}H** ($CDCl_3$, δ, ppm): 1.42 (s, 18H, ^{t}Bu), 1.46 (s, 9H, ^{t}Bu), 2.42 (s, 3H, $C_6H_4\underline{Me}$), 2.70-2.74 (m,

4H, NCH_2), 2.75-2.89 (m, 12H, NCH_2), 3.23 (s, 4H, $C\underline{H}_2COO^tBu$), 3.34 (s, 2H, $C\underline{H}_2COO^tBu$), 3.83 (s, 2H, $PyC\underline{H}_2$), 7.29-7.33 (m, 2H, $C_6\underline{H}_4Me$), 7.53-7.56 (m, 2H, $C_6\underline{H}_4Me$), 7.68-7.71 and 8.24-8.26 (both m, H-3′ and H-5′(Py)), 7.78 (dd, 1H, ^3J 7.6, 7.6 Hz, H-4′(Py)), 7.98 (dd, 1H, ^3J 8.2 Hz, ^4J 2.4 Hz, H-4(Py)), 8.46 (d, 1H, ^3J 8.2 Hz, H-3(Py)), 8.89 (d, 1H, ^4J 2.4 Hz, H-6(Py)). **ESI-MS**, *m/z*: 773.50 $(M+H)^+$.

***tert*-Butyl 2,2′,2″-(10-((5′-(4-chlorophenyl)-2,2′-bipyridin-6-yl)methyl)-1,4,7,10-tetraazacyclododecane-1,4,7-triyl)triacetate (3d).** Yield 198 mg (0.25 mmol, 62%). **NMR ^{1}H** ($CDCl_3$, δ, ppm): 1.42 (s, 18H, ^{t}Bu), 1.46 (s, 9H, ^{t}Bu), 2.70-2.74 (m, 4H, NCH_2), 2.75-2.89 (m, 12H, NCH_2), 3.23 (s, 4H, $C\underline{H}_2COO^tBu$), 3.34 (s, 2H, $C\underline{H}_2COO^tBu$), 3.83 (s, 2H, $PyC\underline{H}_2$), 7.45-7.49 (m, 2H, C_6H_4Cl), 7.56-7.60 (m, 2H, C_6H_4Cl), 7.70-7.73 and 8.24-8.27 (both m, H-3′ and H-5′(Py)), 7.79 (dd, 1H, ^3J 7.6, 7.6 Hz, H-4′(Py)), 7.97 (dd, 1H, ^3J 8.2 Hz, ^4J 2.4 Hz, H-4(Py)), 8.49 (d, 1H, ^3J 8.2 Hz, H-3(Py)), 8.87 (d, 1H, ^4J 2.4 Hz, H-6(Py)). **ESI-MS**, *m/z*: 793.44 $(M+H)^+$.

***tert*-Butyl 2,2′,2″-(10-((5′-(3-chlorophenyl)-2,2′-bipyridin-6-yl)methyl)-1,4,7,10-tetraazacyclododecane-1,4,7-triyl)triacetate (3e).** Yield 238 mg (0.30 mmol, 75%). **NMR ^{1}H** ($CDCl_3$, δ, ppm): 1.41 (s, 18H, ^{t}Bu), 1.46 (s, 9H, ^{t}Bu), 2.70-2.74 (m, 4H, NCH_2), 2.85-2.89 (m, 12H, NCH_2), 3.23 (s, 4H, $C\underline{H}_2COO^tBu$), 3.34 (s, 2H, $C\underline{H}_2COO^tBu$), 3.83 (s, 2H, $PyC\underline{H}_2$), 7.37-7.40 and 7.51-7.54 (both m, 1H, H-4 and H-6(C_6H_3Cl)), 7.43 (dd, 1H, ^3J 7.6, 7.6 Hz, H-5(C_6H_3Cl)), 7.62 (dd, 1H, ^4J 1.6, 1.6 Hz, H-2(C_6H_3Cl)), 7.71-7.74 and 8.25-8.28 (both m, H-3′ and H-5′(Py)), 7.79 (dd, 1H, ^3J 7.6, 7.6 Hz, H-4′(Py)), 7.97 (dd, 1H, ^3J 8.2 Hz, ^4J 2.4 Hz, H-4(Py)), 8.50 (d, 1H, ^3J 8.2 Hz, H-3(Py)), 8.87 (d, 1H, ^4J 2.4 Hz, H-6(Py)). **ESI-MS**, *m/z*: 793.44 $(M+H)^+$.

***tert*-Butyl 2,2′,2″-(10-((4-(4-methoxyphenyl)-2,2′-bipyridin-6-yl)methyl)-1,4,7,10-tetraazacyclododecane-1,4,7-triyl)triacetate (3f).** Yield 182 mg (0.23 mmol, 58%). **NMR ^{1}H** ($CDCl_3$, δ, ppm): 1.38 (s, 18H, ^{t}Bu), 1.45 (s, 9H, ^{t}Bu), 2.74-2.77 (m, 4H, NCH_2), 2.88-2.92 (m, 12H, NCH_2), 3.21 (s, 4H, $C\underline{H}_2COO^tBu$), 3.34 (s, 2H, $C\underline{H}_2COO^tBu$), 3.87 (s, 2H, $PyC\underline{H}_2$), 3.88 (s, 3H, OMe), 6.99-7.03 (m, 2H, $C_6\underline{H}_4OMe$), 7.28-7.32 (m, 1H, H-5′(Py)), 7.78-7.83 (m, 3H, $C_6\underline{H}_4OMe$, H-4′(Py)), 7.92 and 8.48 (both d, 1H, ^4J 1.6 Hz, H-3,5(Py)), 8.43-8.46 (m, 1H, H-3′(Py)), 8.68-8.71 (m, 1H, H-6′(Py)). **ESI-MS**, *m/z*: 789.49 $(M+H)^+$.

***tert*-Butyl 2,2′,2″-(10-((4-(3-chlorophenyl)-2,2′-bipyridin-6-yl)methyl)-1,4,7,10-tetraazacyclododecane-1,4,7-triyl)triacetate (3g).** Yield 222 mg (0.28 mmol, 69%). **NMR ^{1}H** ($CDCl_3$, δ, ppm): 1.38 (s, 18H, ^{t}Bu), 1.45 (s, 9H, ^{t}Bu), 2.73-2.77 (m, 4H, NCH_2), 2.86-2.94 (m, 12H, NCH_2), 3.21 (s, 4H, $C\underline{H}_2COO^tBu$), 3.31 (s, 2H, $C\underline{H}_2COO^tBu$), 3.88 (s, 2H, $PyC\underline{H}_2$), 7.30-7.33 (m, 1H, H-5′(Py)), 7.38-7.42 and 7.71-7.74 (both m, H-4 and H-6(C_6H_3Cl)), 7.43 (dd, 1H, ^3J 8.2, 8.2 Hz, H-5(C_6H_3Cl)), 7.80-8.85 (m, 2H, H-2(C_6H_3Cl), H-4′(Py)), 8.00 and

8.49 (both d, 1H, ^4J 1.6 Hz, H-3,5(Py)), 8.45-8.48 (m, 1H, H-3′(Py)), 8.69-8.71 (m, 1H, H-6′(Py)). **ESI-MS**, *m/z*: 793.44 $(M+H)^+$.

***tert*-Butyl 2,2′,2″-(10-((4-(2-fluorophenyl)-2,2′-bipyridin-6-yl)methyl)-1,4,7,10-tetraazacyclododecane-1,4,7-triyl)triacetate (3h).** Yield 140 mg (0.18 mmol, 45%). **NMR ^{1}H** ($CDCl_3$, δ, ppm): 1.39 (s, 18H, ^{t}Bu), 1.45 (s, 9H, ^{t}Bu), 2.76-2.79 (m, 4H, NCH_2), 2.84-2.87 (m, 8H, NCH_2), 2.88-2.92 (m, 4H, NCH_2), 3.22 (s, 4H, C<u>H</u>$_2$COO^{t}Bu), 3.30 (s, 2H, C<u>H</u>$_2$COO^{t}Bu), 3.89 (s, 2H, PyC<u>H</u>$_2$), 7.16-7.21 (m, 1H, C_6H_3F), 7.24-7.28 (m, 1H, C_6H_3F), 7.28-7.32 (m, 1H, H-5′(Py)), 7.36-7.42 (m, 1H, C_6H_3F), 7.63 (ddd, 1H, ^3J 7.6, 7.6 Hz, ^4J 1.6 Hz, C_6H_3F), 7.79-7.84 (m, 2H, H-4′(Py), H-3(Py)), 8.44-8.47 (m, 2H, H-3′(Py), H-5(Py)), 8.67-8.70 (m, 1H, H-6′(Py)). **NMR ^{19}F** ($CDCl_3$, δ, ppm): -116.43. **ESI-MS**, *m/z*: 777.47 $(M+H)^+$.

***tert*-Butyl 2,2′,2″-(10-((5-phenyl-2,2′-bipyridin-6-yl)methyl)-1,4,7,10-tetraaza-cyclododecane-1,4,7-triyl)triacetate (3i).** Yield 213 mg (0.28 mmol, 70%). **NMR ^{1}H** ($CDCl_3$, δ, ppm): 1.39 (s, 18H, ^{t}Bu), 1.44 (s, 9H, ^{t}Bu), 2.54-2.80 (m, 16H, NCH_2), 3.12 (s, 4H, C<u>H</u>$_2$COO^{t}Bu), 3.28 (s, 2H, C<u>H</u>$_2$COO^{t}Bu), 3.84 (s, 2H, PyC<u>H</u>$_2$), 7.28-7.32 (m, 1H, H-5′(Py)), 7.35-7.39 (m, 1H, Ph), 7.41-7.45 (m, 2H, Ph), 7.47-7.50 (m, 2H, Ph), 7.69 and 8.35 (both d, 1H, ^3J 7.6 Hz, H-3 and H-4(Py)), 7.82 (ddd, 1H, ^3J 7.6, 7.6 Hz, ^4J 1.6 Hz, H-4′(Py)), 8.50-8.53 (m, 1H, H-3′(Py)), 8.66-8.69 (m, 1H, H-6′(Py)). **ESI-MS**, *m/z*: 759.48 $(M+H)^+$.

*General method for the obtaining of esters **9b,c***

The ester of DTTA **6** (218 mg, 0.39 mmol) was dissolved in dry acetonitrile (40 ml), the corresponding bromomethylbipyridine **1b-c** (0.35 mmol) and potassium carbonate (244 mg, 1.77 mmol) were added and the resulting mixture was stirred under reflux for 8 h in argon atmosphere. Then solvent was removed under reduced pressure, water (30 ml) was added to the residue, the product was extracted with DCM (3×20 ml). Extract was washed with anhydrous sodium sulfate. Solvent was removed under reduced pressure. The residue was separated by column chromatography (eluent: DCM, then ethylacetate, $R_f = 0.2$).

***tert*-Butyl 2,2′,2″,2‴-(2,2′-((5′-(4-methoxyphenyl)-2,2′-bipyridin-6-yl)methyl-azane-diyl)bis(ethane-2,1-diyl))bis(azanetriyl)tetraacetate (9b).** Dark yellow oil. Yield 175 mg (0.21 mmol, 60%). **NMR ^{1}H** (DMSO-d_6, δ, ppm): 1.40 (s, 36H, Bu^t), 2.66 (t, 4H, ^3J 6.0 Hz, NCH_2), 2.83 (t, 4H, ^3J 6.0 Hz, NCH_2), 3.41 (s, 8H, C<u>H</u>$_2$COOBu^t), 3.87 (s, 5H, PyC<u>H</u>$_2$, OCH_3), 7.13 (m, 2H, C$_6$<u>H</u>$_4$OCH$_3$), 7.54 (m, 1H, H-5), 7.80 (m, 2H, C$_6$<u>H</u>$_4$OCH$_3$), 7.92 (dd, 1H, ^3J 7.6, 7.6 Hz, H-4), 8.20 (dd, 1H, ^3J 8.2 Hz, ^4J 2.4 Hz, H-4′), 8.31 (m, 1H, H-3), 8.47 (d, 1H, ^3J 8.2 Hz, H-3′), 9.00 (d, 1H, ^4J 2.4 Hz, H-6′). **ESI-MS**, *m/z*: 834.50 $(M+H)^+$.

***tert*-Butyl 2,2′,2″,2‴-(2,2′-((5′-*p*-tolyl-2,2′-bipyridin-6-yl)methylazanediyl)bis(ethane-2,1-diyl)bis(azanetriyl))tetraacetate (9c).** Dark yellow oil. Yield 190 mg (0.23 mmol, 66%). **NMR ^{1}H** (DMSO-d_6, δ, ppm): 1.40 (s, 36H, Bu^t), 2.42 (s, 3H, $C_6H_4C\underline{H}_3$), 2.66 (t, 4H, ^3J 6.0 Hz, NCH_2), 2.83 (t, 4H, ^3J 6.0 Hz, NCH_2), 3.41 (s, 8H, $C\underline{H}_2COOBu^t$), 3.88 (s, 2H, $PyC\underline{H}_2$), 7.38 (m, 2H, $C_6\underline{H}_4CH_3$), 7.54 (m, 1H, H-5′), 7.74 (m, 2H, $C_6\underline{H}_4CH_3$), 7.92 (dd, 1H, ^3J 7.8, 7.8 Hz, H-4′), 8.23 (dd, 1H, ^3J 8.2 Hz, ^4J 2.4 Hz, H-4), 8.32 (m, 1H, H-3′), 8.49 (d, 1H, ^3J 8.2 Hz, H-3), 9.02 (d, 1H, ^4J 2.4 Hz, H-6). **ESI-MS**, *m/z*: 818.51 $(M+H)^+$.

*General method for the obtaining of ligands **4***

The corresponding ester **3** (0.20 mmol) was dissolved in hydrochloric acid (5 N, 10 ml). The resulting mixture was stirred at room temperature for overnight. Then solvent was removed under reduced pressure. The hydrochloric acid (11 N, 7 ml) was added to the residue and the resulting mixture was stirred at room temperature for 2 h. Undissolved part was filtered off, solvent from the filtrate was removed under reduced pressure. Dry acetonitrile (15 ml) was added to the residue, the resulting mixture was stirred at room temperature for overnight. The precipitate formed was filtered off, washed with dry acetonitrile and dried under vacuum.

Ligand 4c. Yield 116 mg (0.15 mmol, 75%). Found, %: C 49.41, H 6.34, N 10.87, Cl 14.25. Calculated for $C_{32}H_{40}N_6O_6$•3.1HCl•3.1H_2O: C 49.68, H 6.42, N 10.86, Cl 14.21.

Ligand 4d. Yield 128 mg (0.16 mmol, 80%). Found, %: C 46.20, H 5.44, N 10.77, Cl 21.24. Calculated for $C_{31}H_{37}ClN_6O_6$•3.8HCl•2.2H_2O: C 46.35, H 5.67, N 10.46, Cl 21.18.

Ligand 4e. Yield 100 mg (0.12 mmol, 60%). Found, %: C 44.01, H 6.30, N 10.95, Cl 18.39. Calculated for $C_{31}H_{37}ClN_6O_6$•3.5HCl•4.5H_2O: C 44.65, H 5.98, N 10.08, Cl 19.13.

Ligand 4f. Yield 108 mg (0.13 mmol, 65%). Found, %: C 45.45, H 6.37, N 10.90, Cl 14.97. Calculated for $C_{32}H_{40}N_6O_7$•3.75HCl•4H_2O: C 46.34, H 6.29, N 10.13, Cl 16.03.

Ligand 4g. Yield 112 mg (0.14 mmol, 70%). Found, %: C 45.86, H 5.49, N 10.94, Cl 18.84. Calculated for $C_{31}H_{37}ClN_6O_6$•3.25HCl•3.25H_2O: C 46.42, H 5.87, N 10.48, Cl 18.78.

Ligand 4h. Yield 123 mg (0.15 mmol, 75%). Found, %: C 45.00, H 5.82, N 10.21, Cl 17.68. Calculated for $C_{31}H_{37}FN_6O_6$•4HCl•11/3H_2O: C 45.38, H 5.94, N 10.24, Cl 17.28.

Ligand 4i. Yield 118 mg (0.15 mmol, 75%). Found, %: C 47.31, H 6.09, N 10.76, Cl 13.39. Calculated for $C_{31}H_{38}N_6O_6$•3HCl•4.5H_2O: C 47.67, H 6.45, N 10.76, Cl 13.62.

*General method for the obtaining of ligands **5***

The corresponding ester **9** (0.1 mmol) was dissolved in hydrochloric acid (5 N, 10 ml). The resulting mixture was stirred at room temperature for overnight. Then solvent was

removed under reduced pressure. The hydrochloric acid (11 N, 7 ml) was added to the residue and the resulting mixture was stirred at room temperature for 2 h. Undissolved part was filtered off, solvent from the filtrate was removed under reduced pressure. Dry acetonitrile (15 ml) was added to the residue, the resulting mixture was stirred at room temperature for overnight. The precipitate formed was filtered off, washed with dry acetonitrile and dried under vacuum.

Ligand 5b. Yield 65 mg (0.08 mmol, 80%). Found, %: C 44.59, H 5.49, N 8.51, Cl 15.92. Calculated for $C_{30}H_{35}N_5O_9$•11/3HCl•11/3H_2O: C 44.52, H 5.73, N 8.65, Cl 16.06.

Ligand 5c. Yield 59 mg (0.07 mmol, 73%). Found, %: C 44.13, H 5.62, N 8.40, Cl 16.36. Calculated for $C_{30}H_{35}N_5O_8$•11/3HCl•14/3H_2O: C 44.41, H 5.96, N 8.63, Cl 16.02.

*General method for the synthesis of lanthanide complexes of ligands **4***

The corresponding ligand **4** (0.06 mmol) was dissolved in water (10 ml). Sodium hydroxide (the number of equivalents determined by elemental analysis and additional 3 equivalents (7.2 mg, 0.18 mmol)). Then the salt of corresponding lanthanide $LnCl_3$•6H_2O (0.06 mmol) was added, the resulting mixture was stirred at room temperature for 2 h. Solvent was removed under reduced pressure. The product was extracted from the residue by hot methanol (3×20 ml). Undissolved part was filtered off, methanol from the filtrate was removed under reduced pressure, product was dried under vacuum.

Eu•4c. Yield 30 mg (0.036 mmol, 61%). Found, %: C 46.42, H 5.27, N 10.33. Calculated for $C_{32}H_{37}EuN_6O_6$•4H_2O: C 46.55, H 5.49, N 10.18. **ESI-MS**, *m/z*: 777.19 $[M+Na]^+$.

Eu•4d. Yield 33 mg (0.039 mmol, 65%). Found, %: C 44.12, H 5.14, N 10.11. Calculated for $C_{31}H_{34}ClEuN_6O_6$•4H_2O: C 44.00, H 5.00, N 9.93. **ESI-MS**, *m/z*: 775.15 $[M+H]^+$.

Eu•4e. Yield 33 mg (0.038 mmol, 64%). Found, %: C 43.22, H 5.02, N 9.58. Calculated for $C_{31}H_{34}ClEuN_6O_6$•5H_2O: C 43.09, H 5.13, N 9.73. **ESI-MS**, *m/z*: 797.13 $[M+Na]^+$.

Eu•4f. Yield 32 mg (0.038 mmol, 63%). Found, %: C 45.79, H 5.26, N 9.80. Calculated for $C_{32}H_{37}EuN_6O_7$•4H_2O: C 45.66, H 5.39, N 9.98. **ESI-MS**, *m/z*: 793.18 $[M+Na]^+$.

Tb•4f. Yield 36 mg (0.042 mmol, 70%). Found, %: C 45.16, H 5.22, N 9.72. Calculated for $C_{32}H_{37}TbN_6O_7$•4H_2O: C 45.29, H 5.34, N 9.90. **ESI-MS**, *m/z*: 799.19 $[M+Na]^+$.

Eu•4g. Yield 34 mg (0.039 mmol, 66%). Found, %: C 43.18, H 5.27, N 9.59. Calculated for $C_{31}H_{34}ClEuN_6O_6•5H_2O$: C 43.09, H 5.13, N 9.73. **ESI-MS**, *m/z*: 797.13 $[M+Na]^+$.

Tb•4g. Yield 36 mg (0.041 mmol, 69%). Found, %: C 42.59, H 5.24, N 9.81. Calculated for $C_{31}H_{34}ClTbN_6O_6•5H_2O$: C 42.72, H 5.09, N 9.65. **ESI-MS**, *m/z*: 803.13 $[M+Na]^+$.

Eu•4h. Yield 32 mg (0.039 mmol, 65%). Found, %: C 44.76, H 4.96, N 9.97. Calculated for $C_{31}H_{34}EuFN_6O_6•4H_2O$: C 44.88, H 5.10, N 10.13. **ESI-MS**, *m/z*: 781.16 $[M+Na]^+$.

Tb•4h. Yield 36 mg (0.043 mmol, 72%). Found, %: C 44.64, H 5.18, N 10.19. Calculated for $C_{31}H_{34}TbFN_6O_6•4H_2O$: C 44.50, H 5.06, N 10.05. **ESI-MS**, *m/z*: 787.17 $[M+Na]^+$.

Eu•4i. Yield 33 mg (0.041 mmol, 68%). Found, %: C 45.73, H 5.21, N 10.17. Calculated for $C_{31}H_{35}EuN_6O_6•4H_2O$: C 45.87, H 5.34, N 10.35. **ESI-MS**, *m/z*: 763.17 $[M+Na]^+$.

Tb•4i. Yield 33 mg (0.039 mmol, 66%). Found, %: C 44.34, H 5.53, N 9.87. Calculated for $C_{31}H_{35}TbN_6O_6•5H_2O$: C 44.50, H 5.42, N 10.04. **ESI-MS**, *m/z*: 769.18 $[M+Na]^+$.

General method for the synthesis of lanthanide complexes of ligands ***5***

The corresponding ligand **9** (0.06 mmol) was dissolved in water (10 ml). Sodium hydroxide (the number of equivalents determined by elemental analysis and additional 4 equivalents (10 mg, 0.24 mmol)). Then the salt of corresponding lanthanide $LnCl_3•6H_2O$ (0.06 mmol) was added, the resulting mixture was stirred at room temperature for 2 h. Solvent was removed under reduced pressure. The product was extracted from the residue by hot methanol (3×20 ml). Undissolved part was filtered off, methanol from the filtrate was removed under reduced pressure, product was dried under vacuum.

Eu•5b. Yield 37 mg (0.043 mmol, 73%). Found, %: C 41.37, H 4.53, N 8.08. Calculated for $C_{30}H_{31}EuN_5NaO_9•4H_2O$: C 42.26, H 4.61, N 8.21. **ESI-MS**, *m/z*: 782.13 $[M+H]^+$.

Eu•5c. Yield 40 mg (0.047 mmol, 77%). Found, %: C 42.03, H 4.72, N 8.05. Calculated for $C_{30}H_{31}EuN_5NaO_8•5H_2O$: C 42.16, H 4.84, N 8.19. **ESI-MS**, *m/z*: 766.14 $[M+H]^+$.

Eu•5d. Yield 36 mg (0.041 mmol, 68%). Found, %: C 39.92, H 4.24, N 8.11. Calculated for $C_{29}H_{28}ClEuN_5NaO_8$•$5H_2O$: C 39.81, H 4.38, N 8.00. **ESI-MS**, *m/z*: 762.08 [M-Na]$^-$.

Eu•5e. Yield 34 mg (0.040 mmol, 66%). Found, %: C 40.52, H 4.11, N 8.30. Calculated for $C_{29}H_{28}ClEuN_5NaO_8$•$4H_2O$: C 40.64, H 4.23, N 8.17. **ESI-MS**, *m/z*: 762.08 [M-Na]$^-$.

2. Figure S1. ^{1}H NMR (400 MHz, DMSO-d_6) of 8

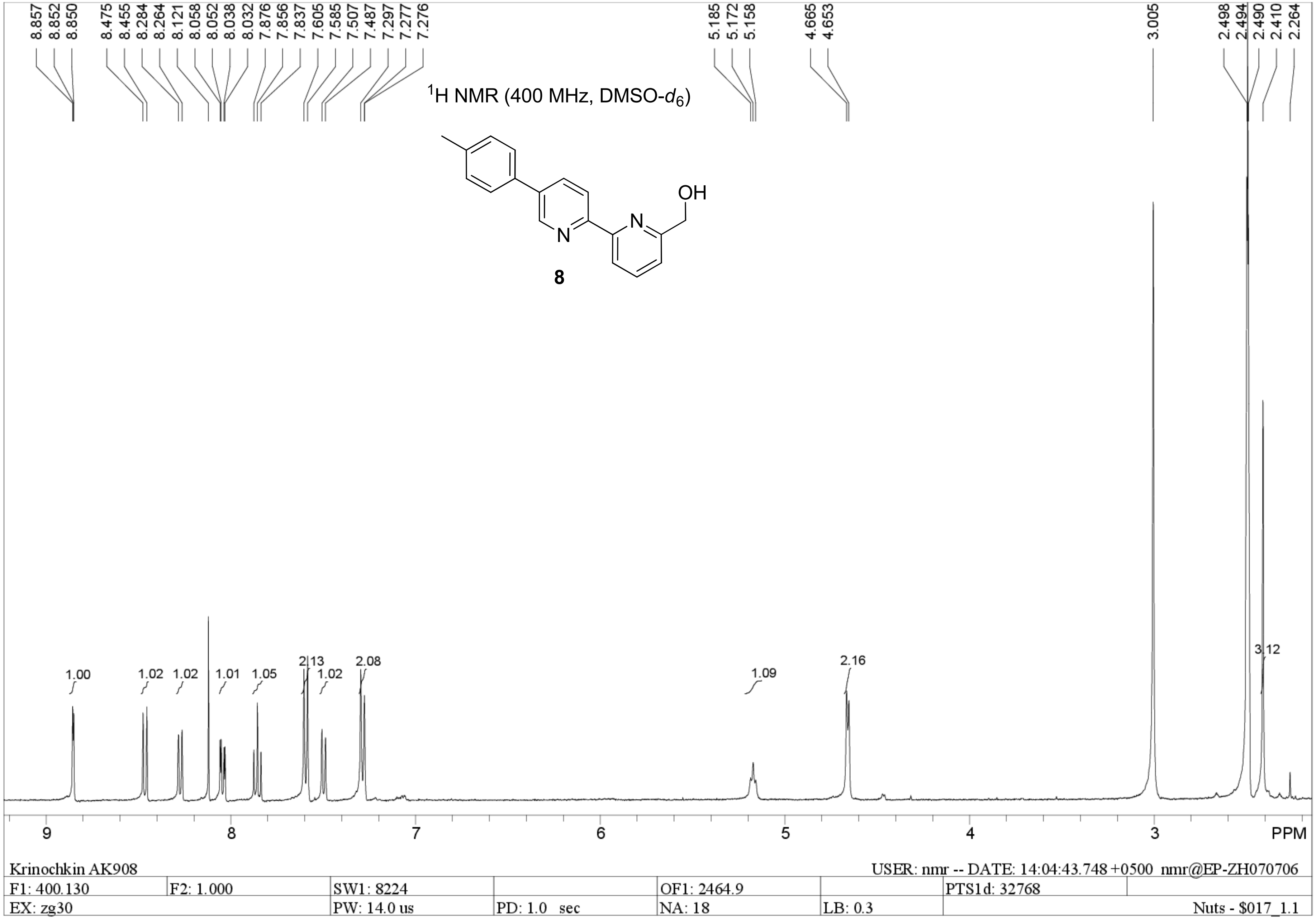

3. Figure S2. ^{1}H NMR (400 MHz, DMSO-d_6) of 1c

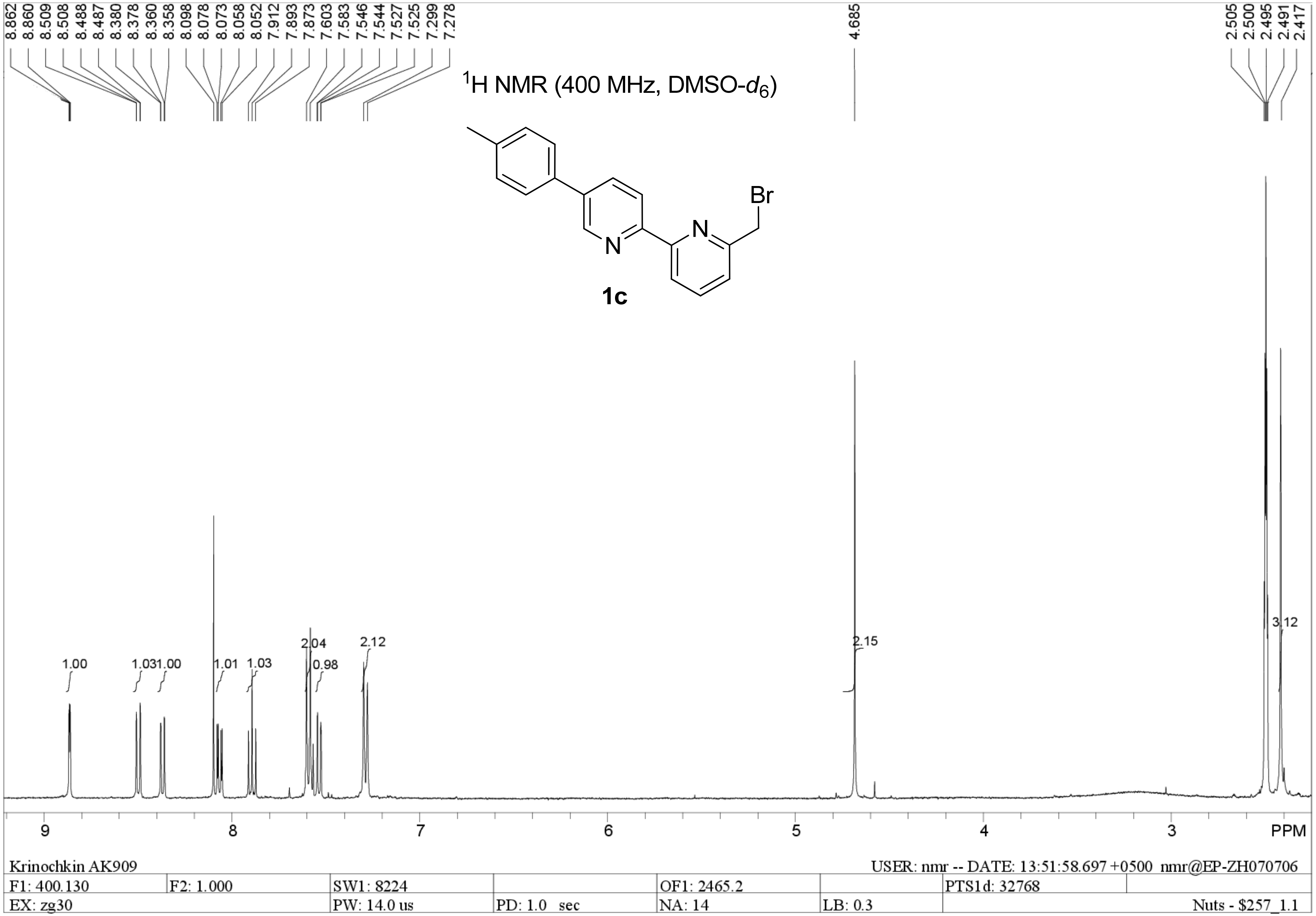

4. Figure S3. ^{1}H NMR (500 MHz, $CDCl_3$) of 3c

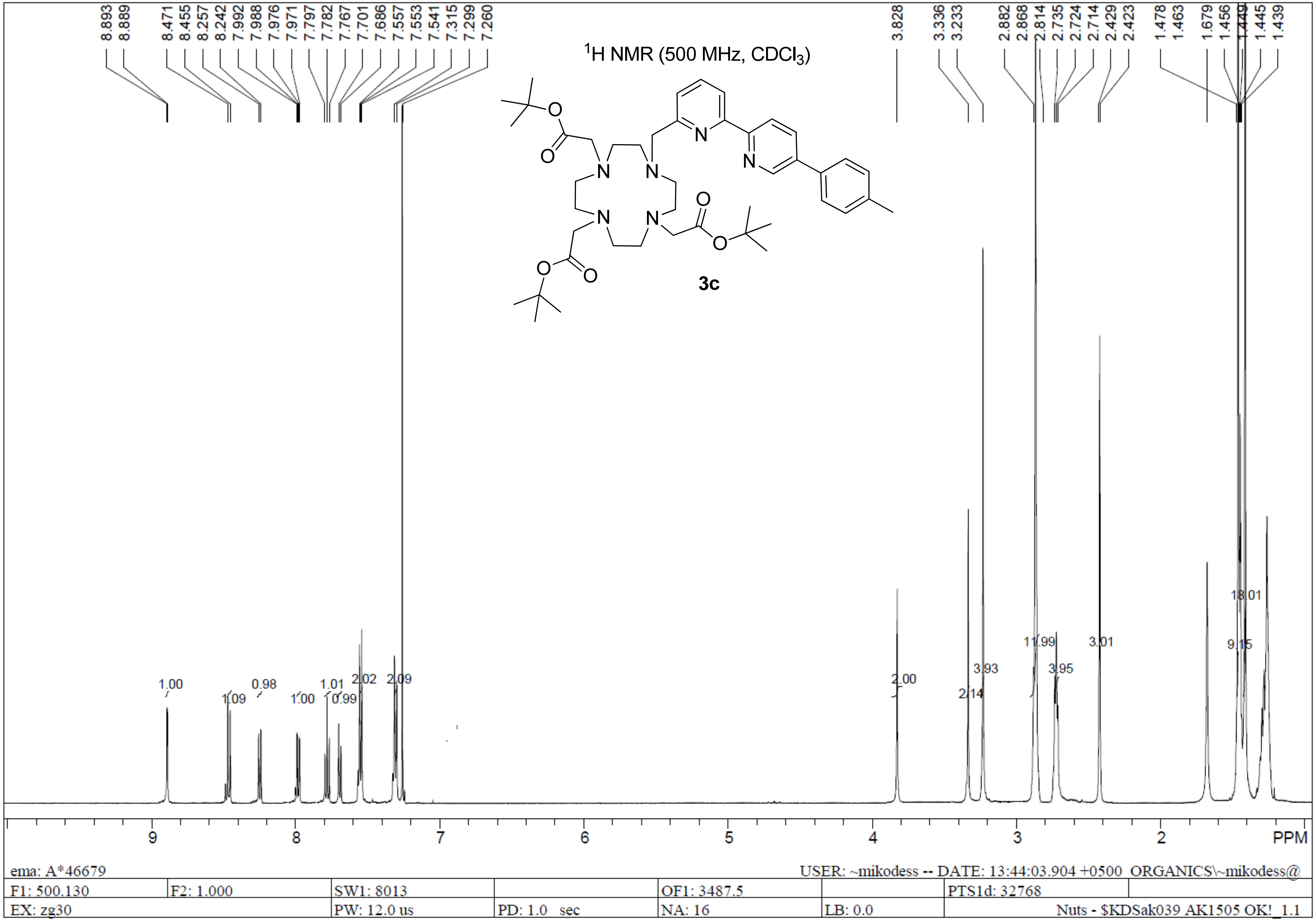

5. Figure S4. ^{1}H NMR (500 MHz, $CDCl_3$) of 3d

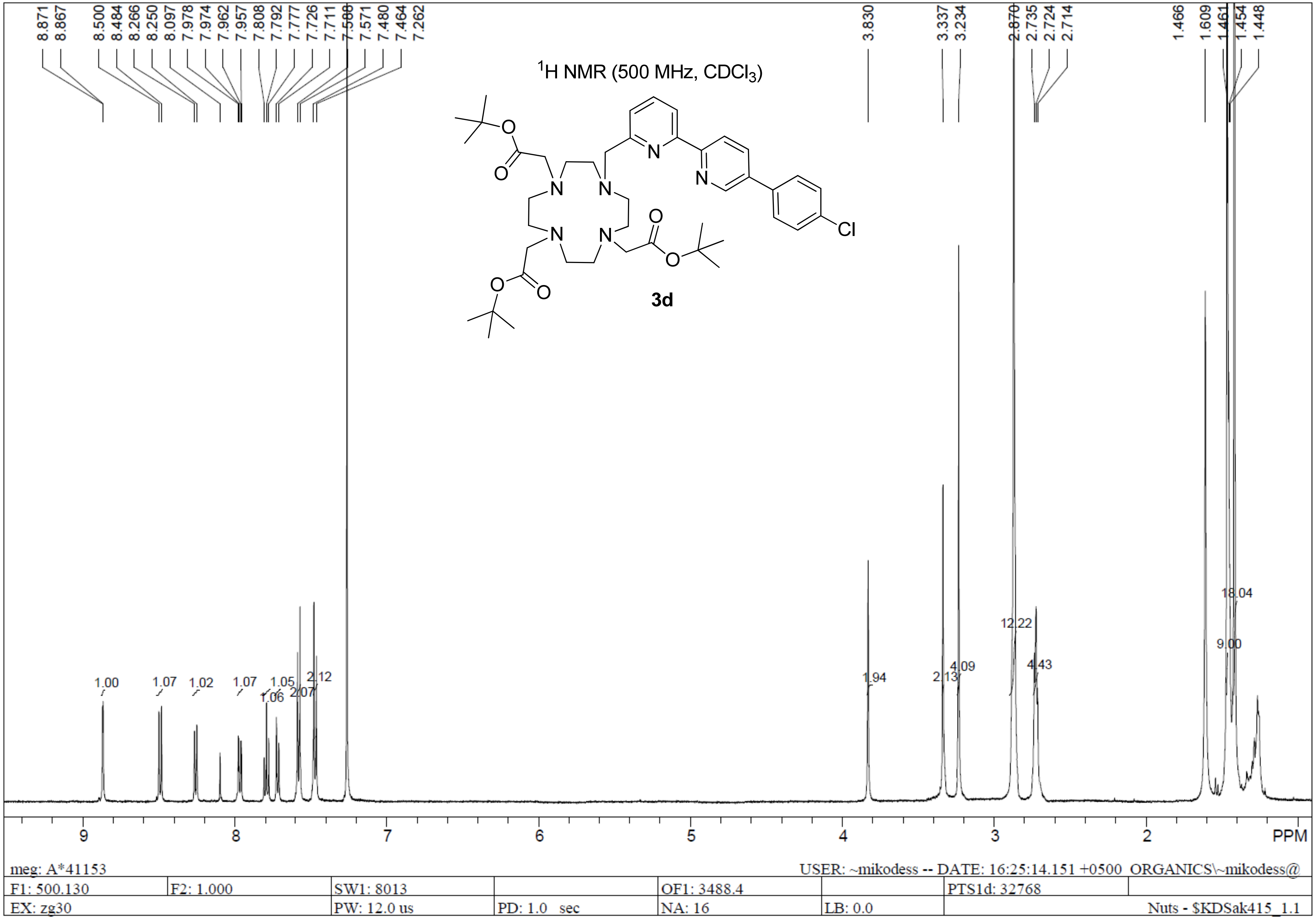

6. Figure S5. ^{1}H NMR (500 MHz, CDCl$_3$) of 3e

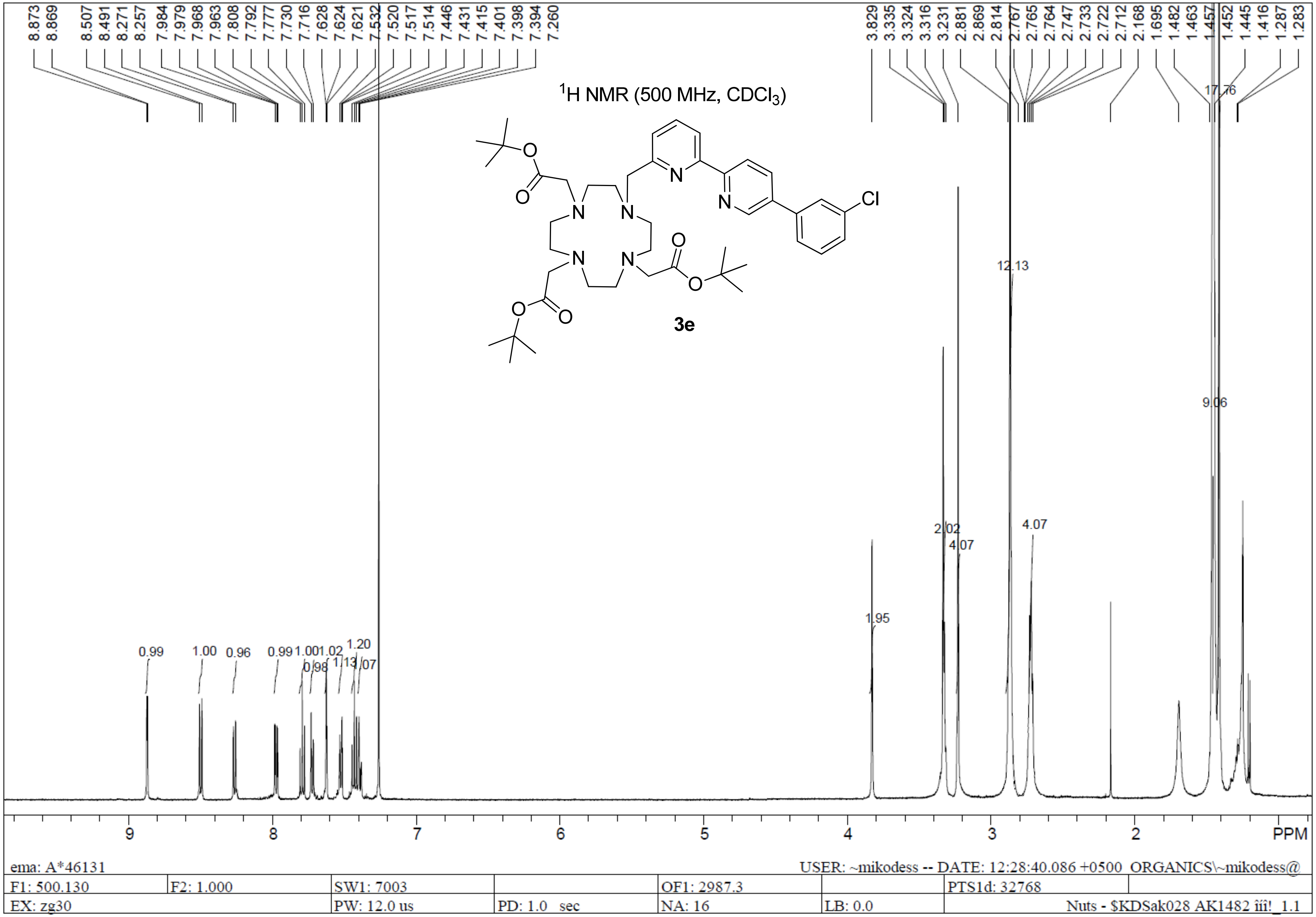

7. **Figure S6.** **^{1}H NMR (500 MHz, $CDCl_3$) of 3f**

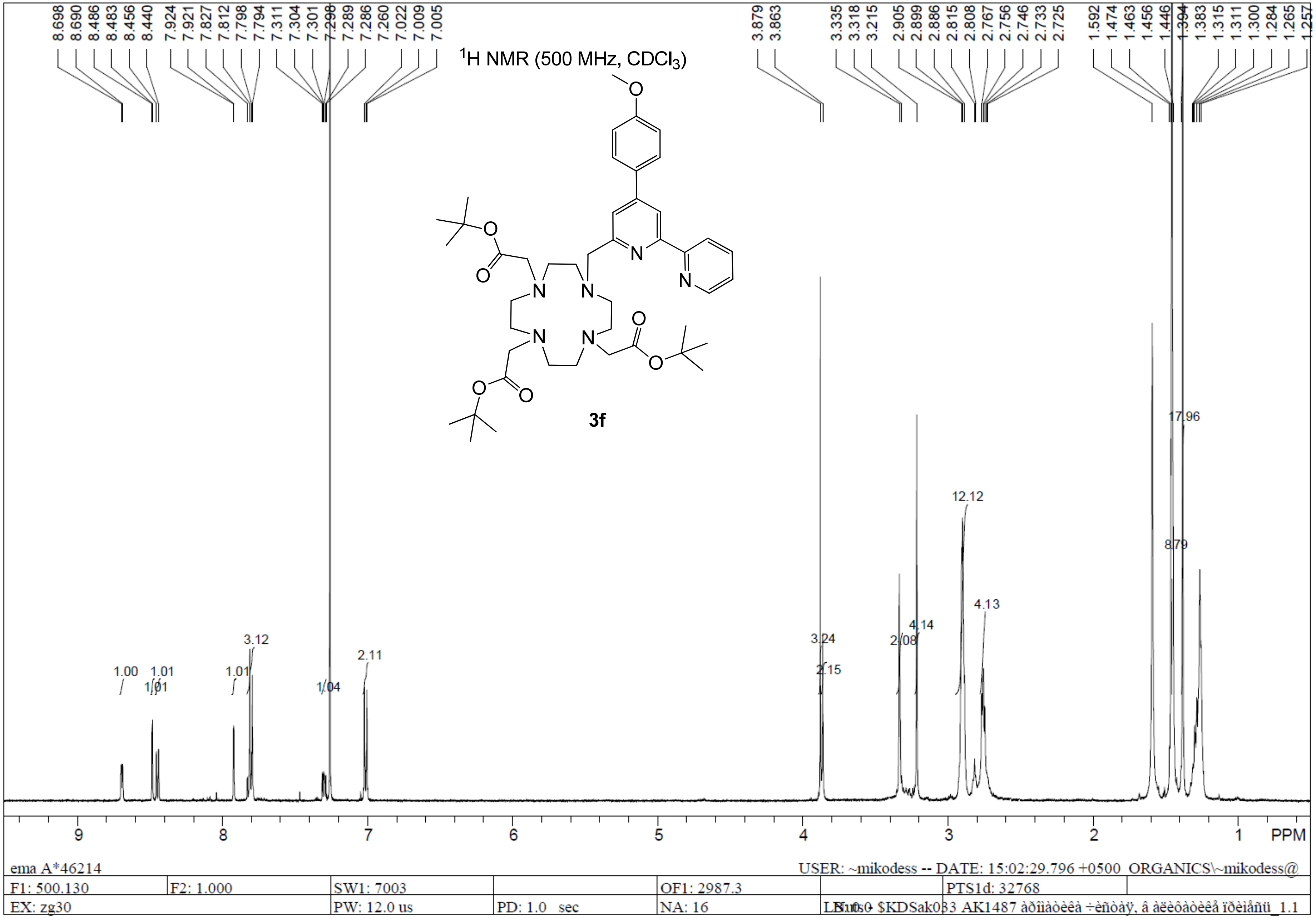

8. Figure S7. ^{1}H NMR (500 MHz, $CDCl_3$) of 3g

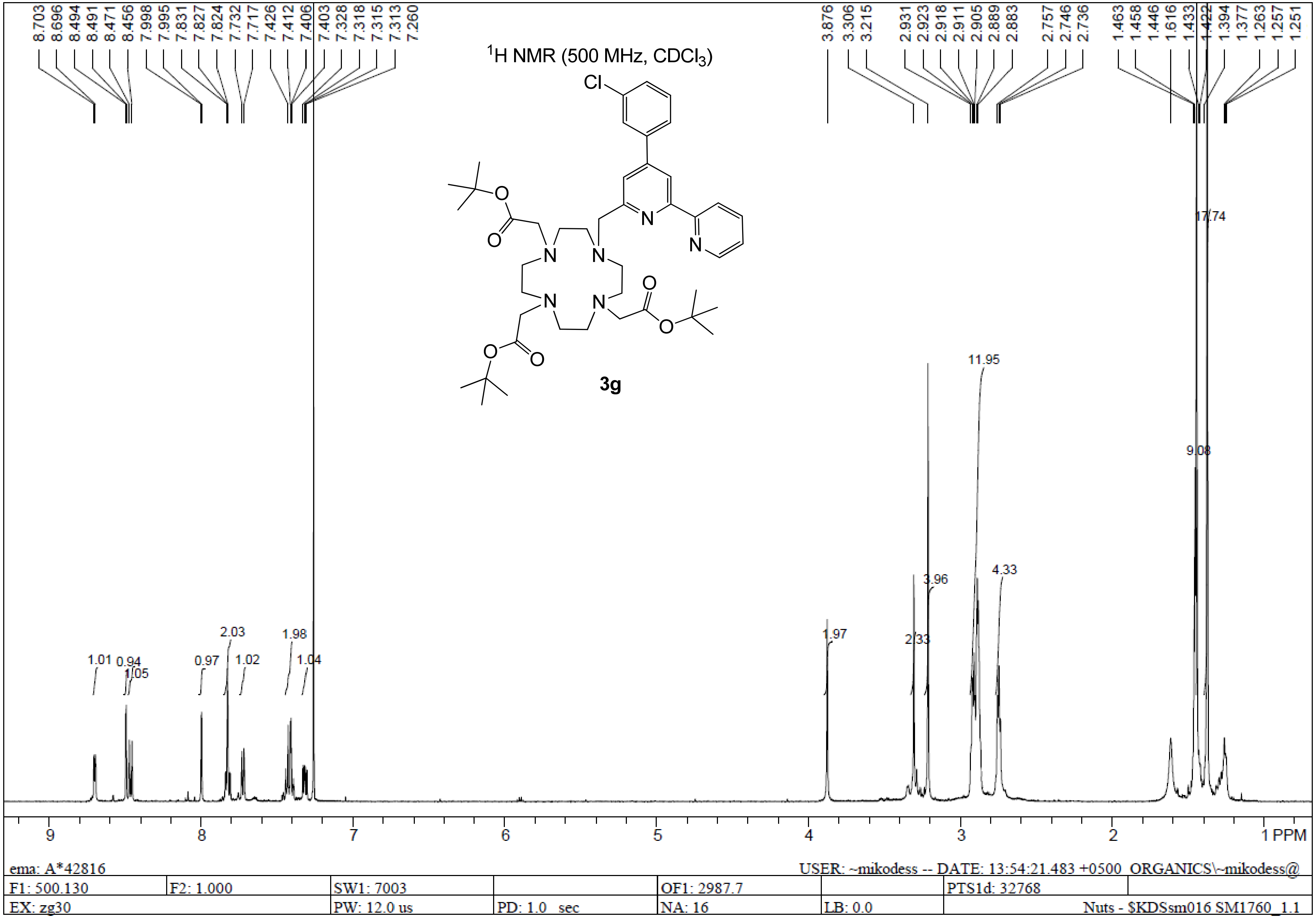

9. Figure S8. ^{1}H NMR (500 MHz, CDCl$_3$) of 3h

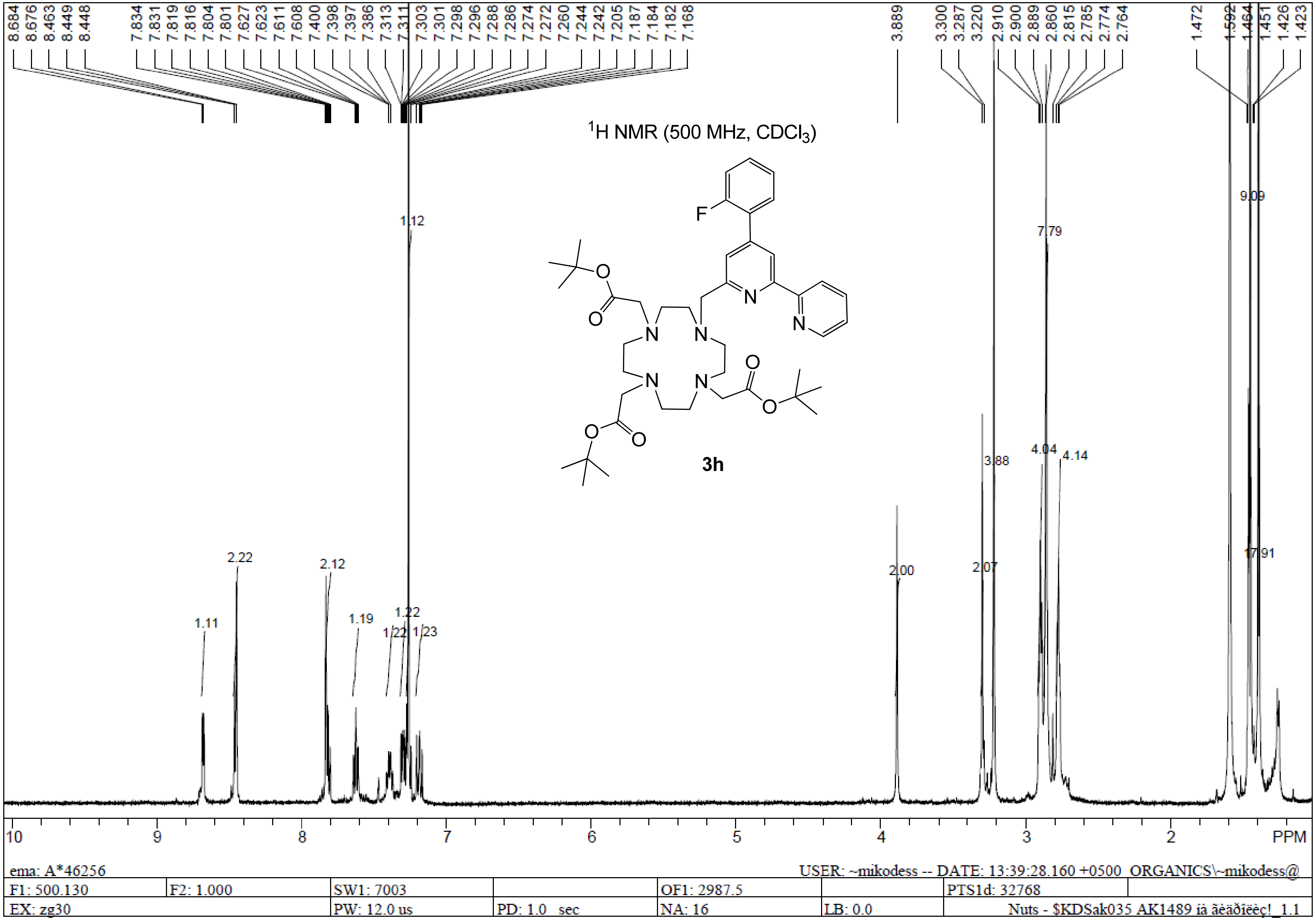

10. Figure S9. ^{19}F NMR (400 MHz, $CDCl_3$) of 3h

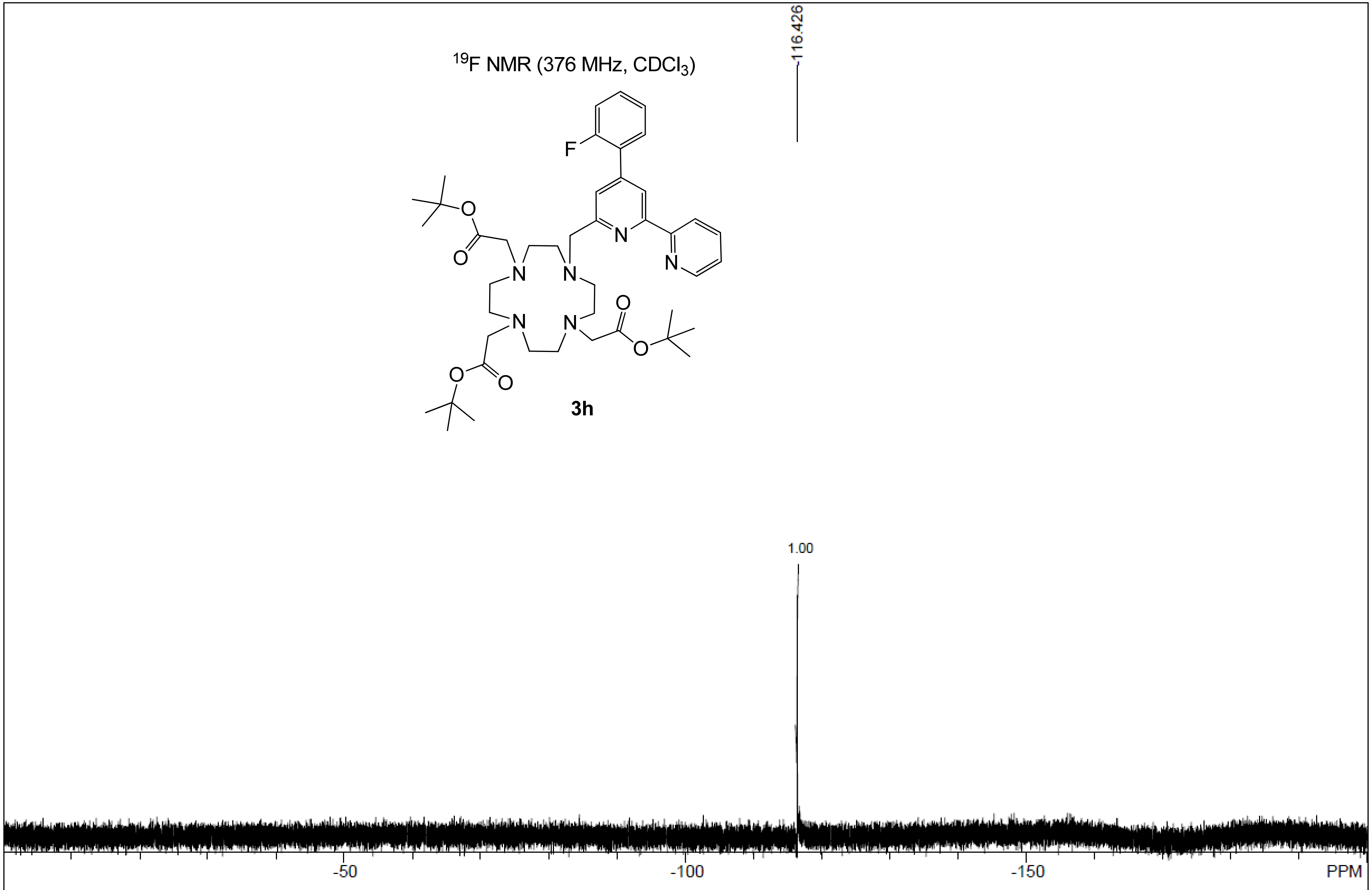

Krinochkin AK 1489 USER: NMR -- DATE: 12:13:54.598 +0300 NMR@NMR_400

F1: 376.498	F2: 1.000	SW1: 89286		OF1: -37649.8		PTS1d: 65536	
EX: zgfhigqn.2		PW: 15.0 us	PD: 1.0 sec	NA: 16	LB: 0.0		Nuts - $084 AK1489 ÎÊ!_19.1

11. Figure S10. ^{1}H NMR (500 MHz, $CDCl_3$) of 3i

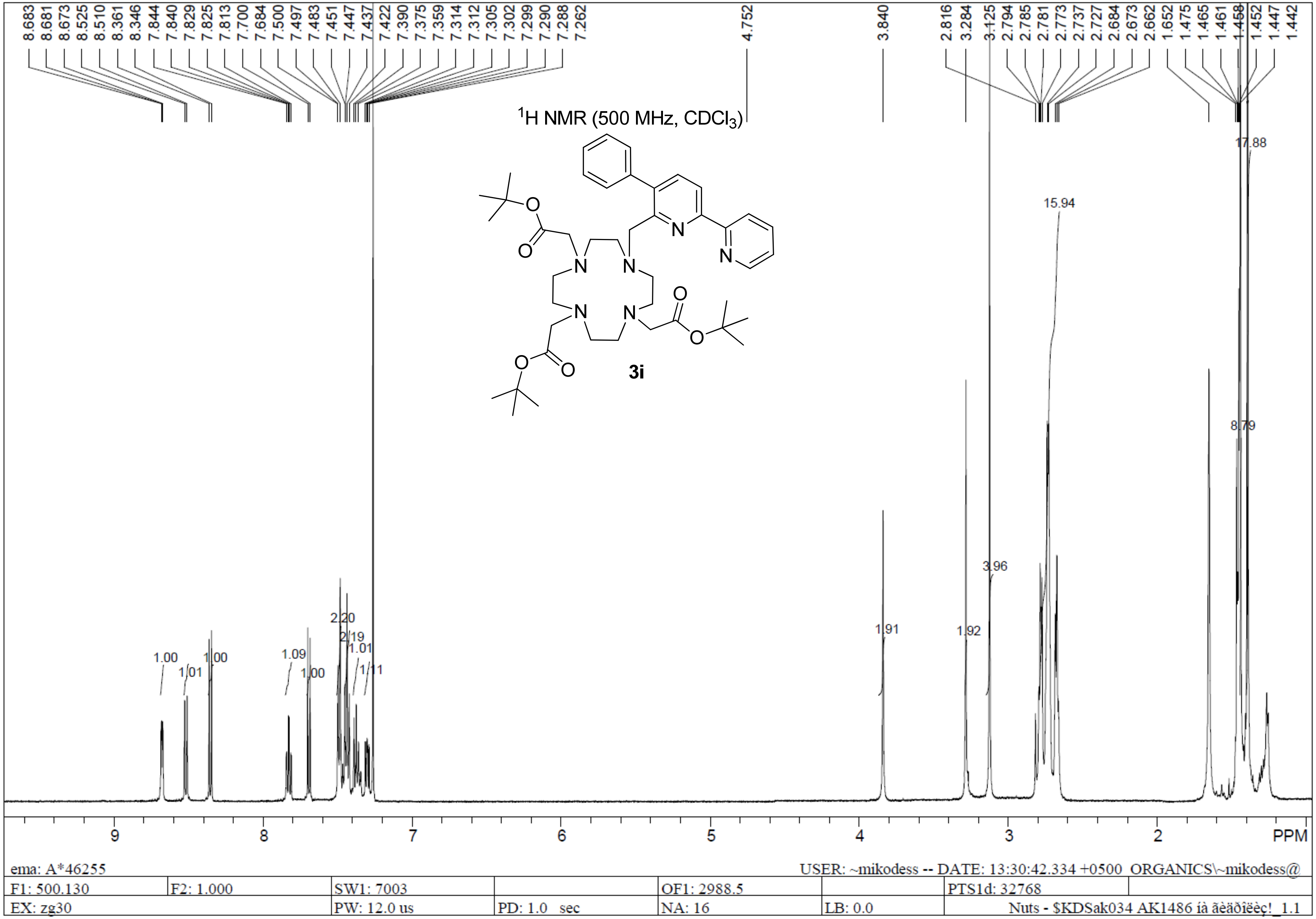

12. Figure S11. ^{1}H NMR (400 MHz, DMSO-d_6) of 9b

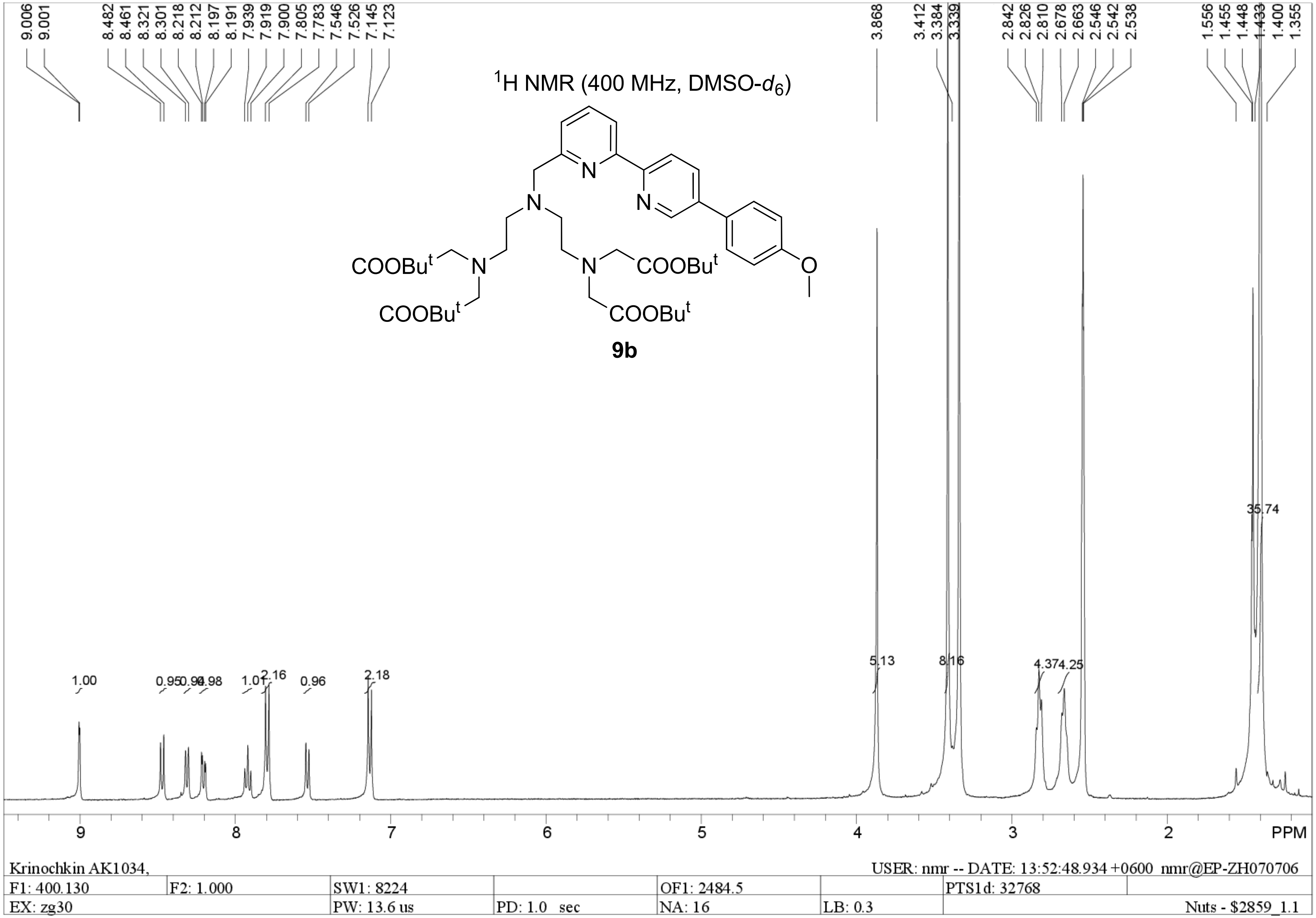

13. Figure S12. ^{1}H NMR (400 MHz, DMSO-d_6) of 9c

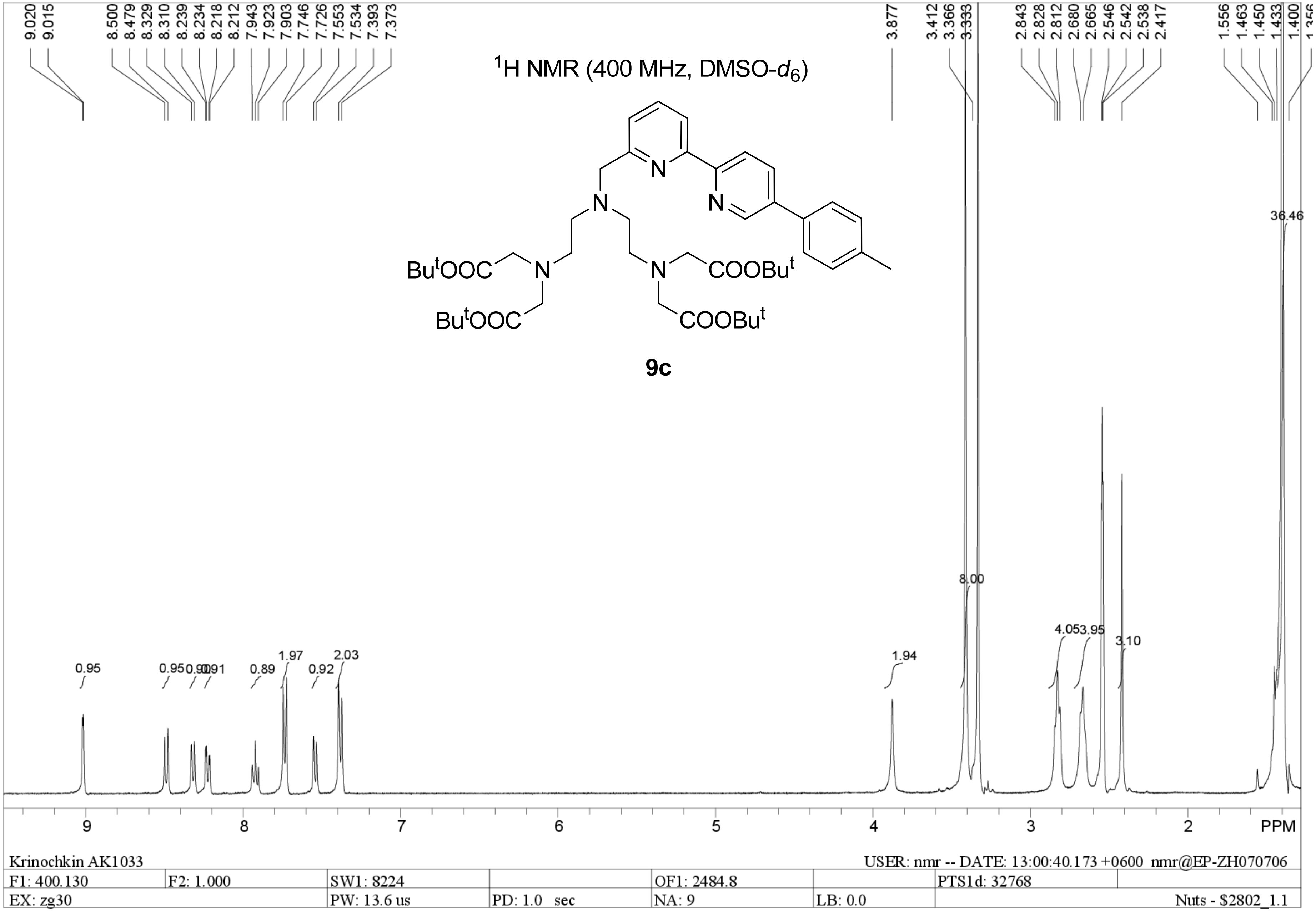

14. Figure S13. Mass-spectrum of Eu•4c

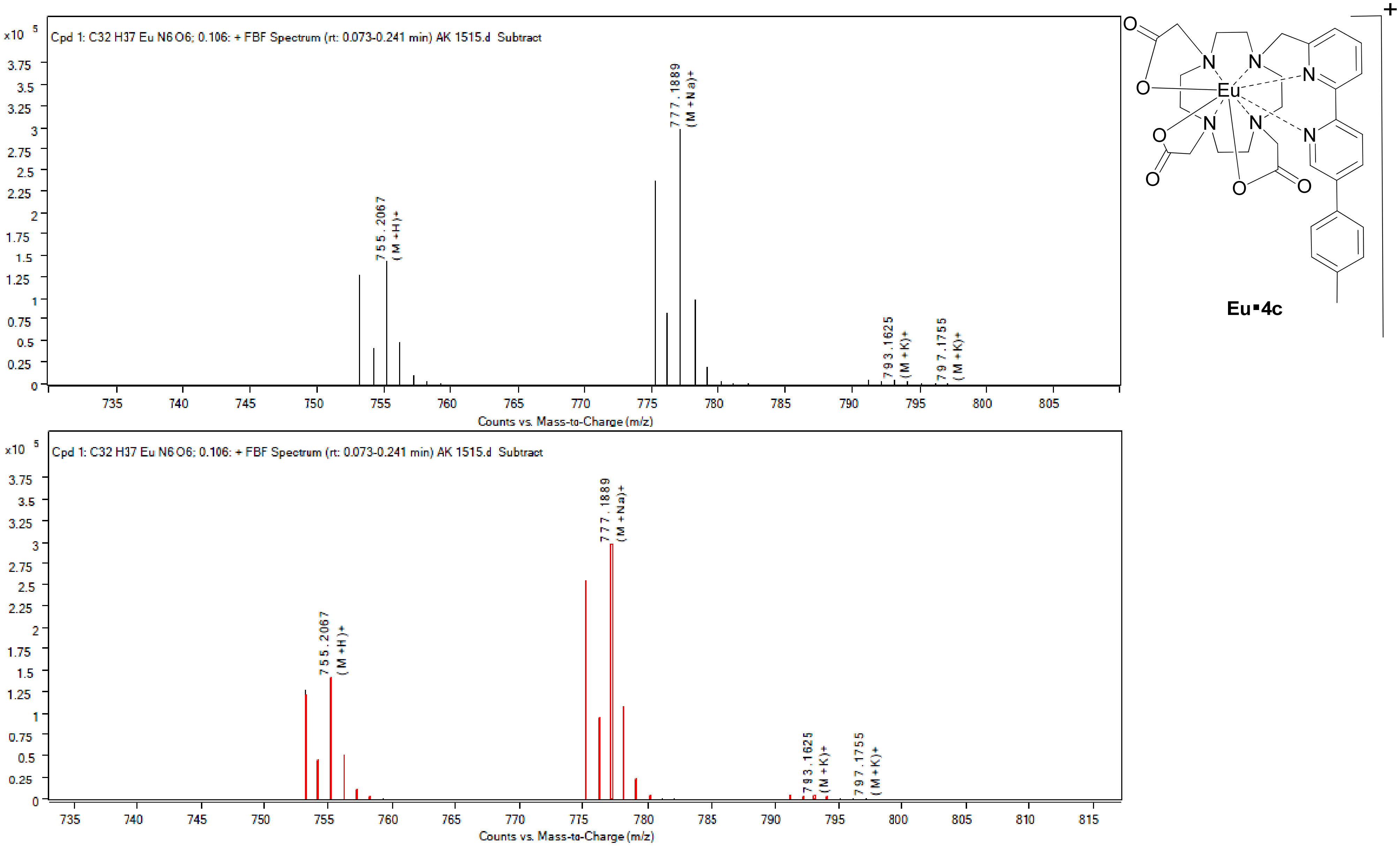

15. Figure S14. Mass-spectrum of Eu•4d

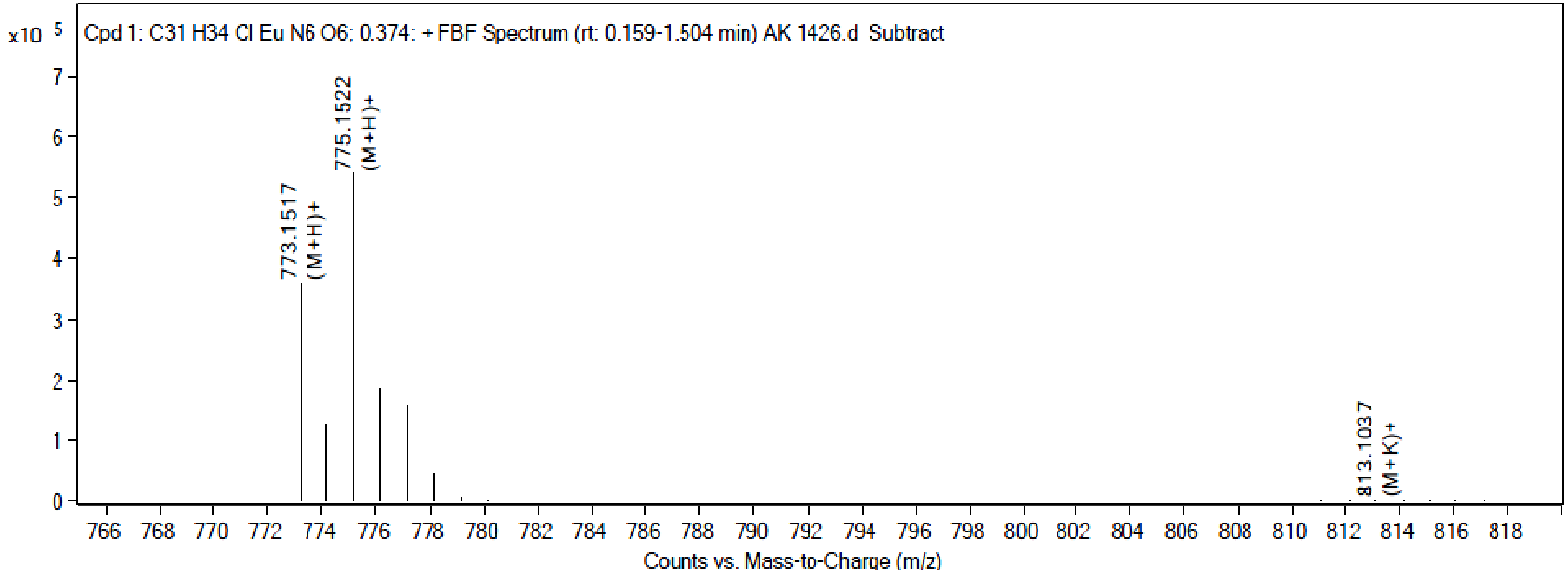

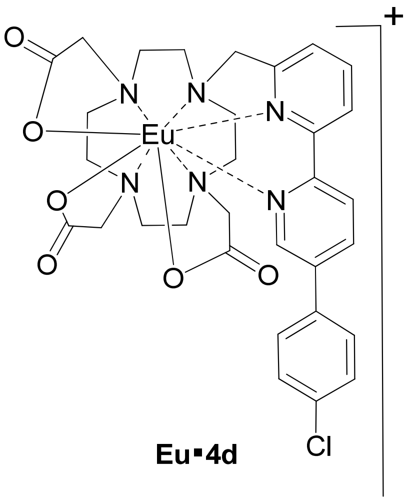

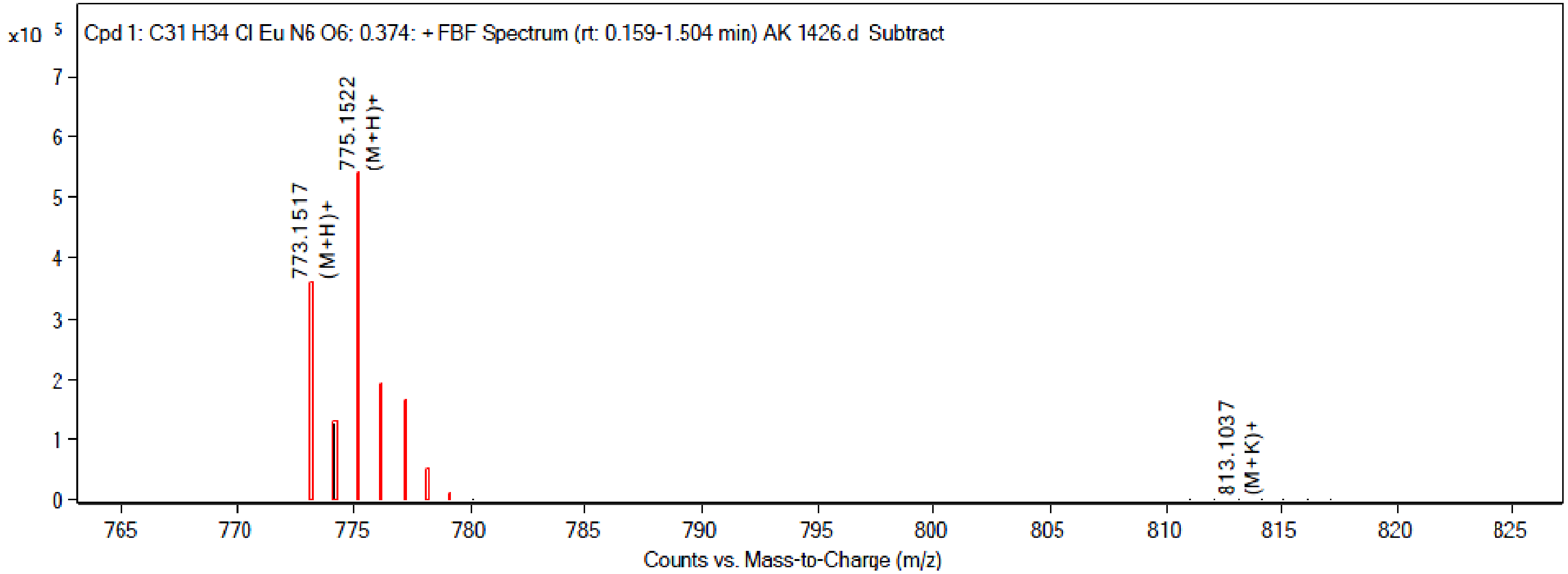

16. Figure S15. Mass-spectrum of Eu•4e

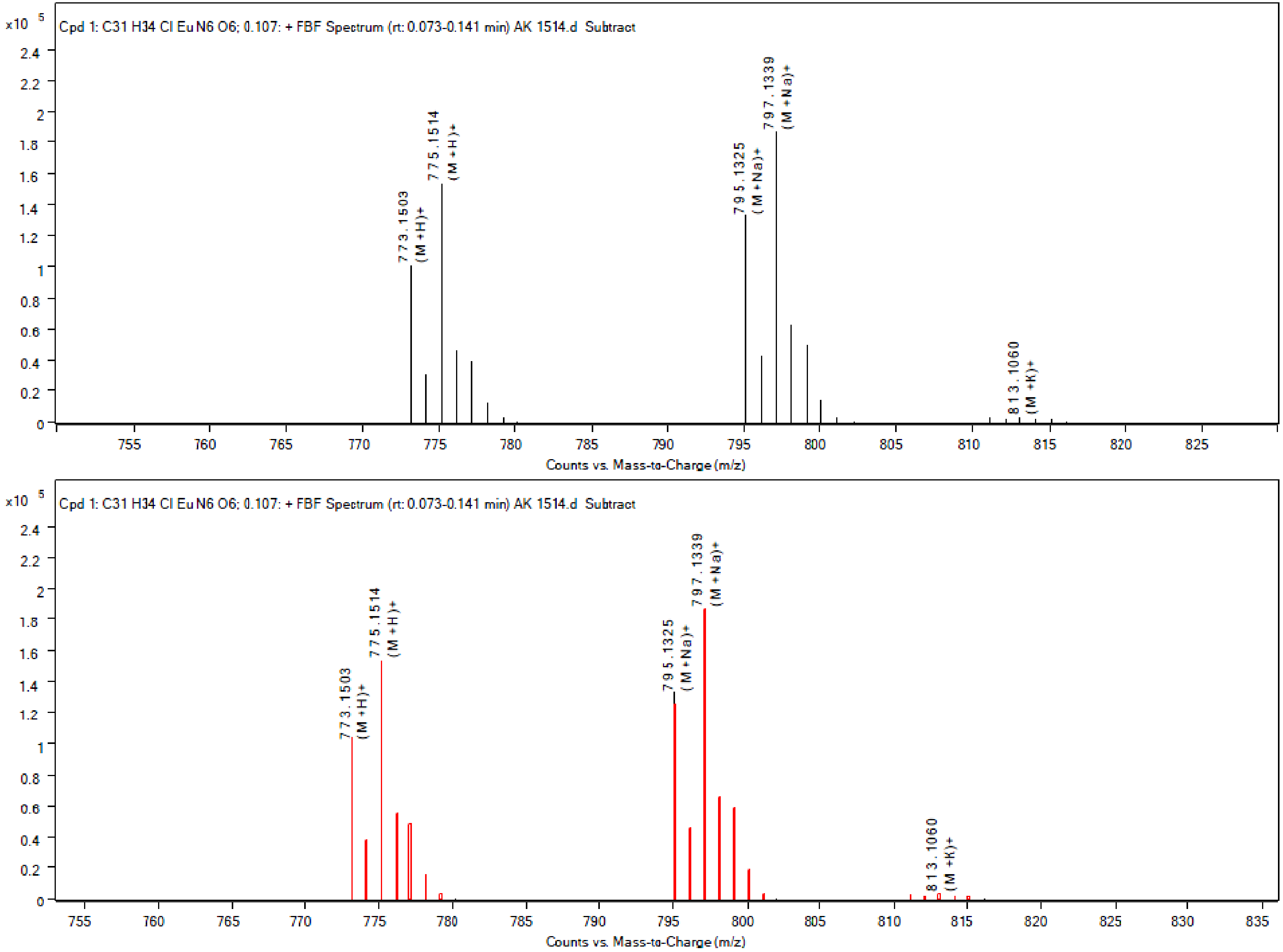

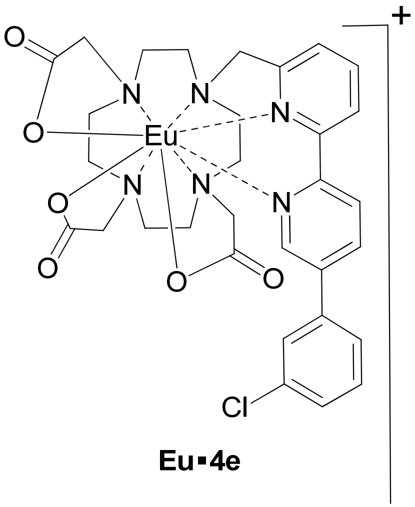

17. Figure S16. Mass-spectrum of Eu•4f

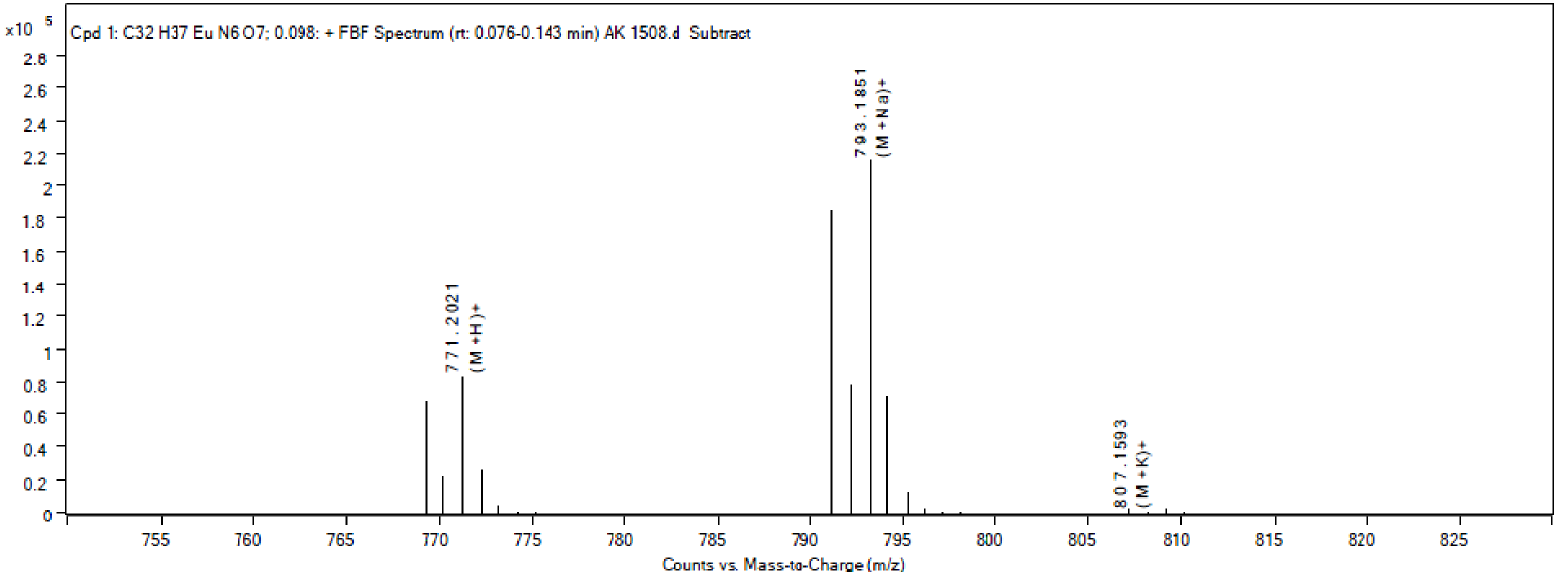

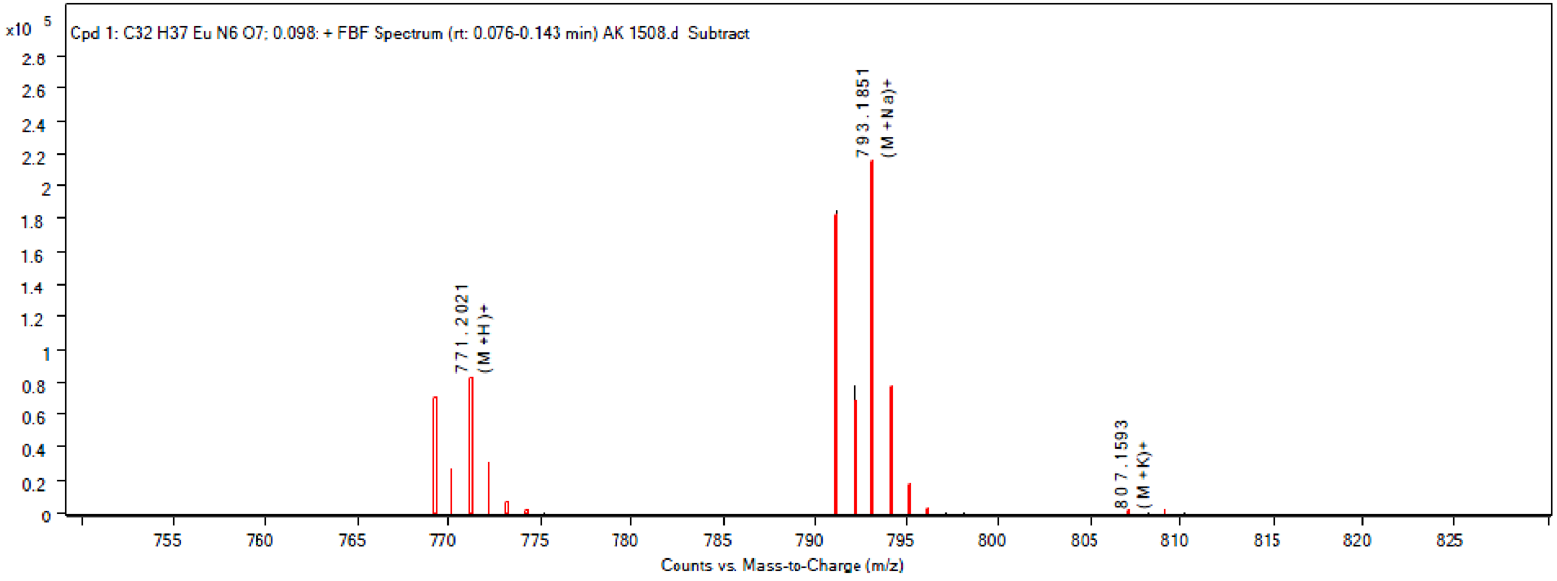

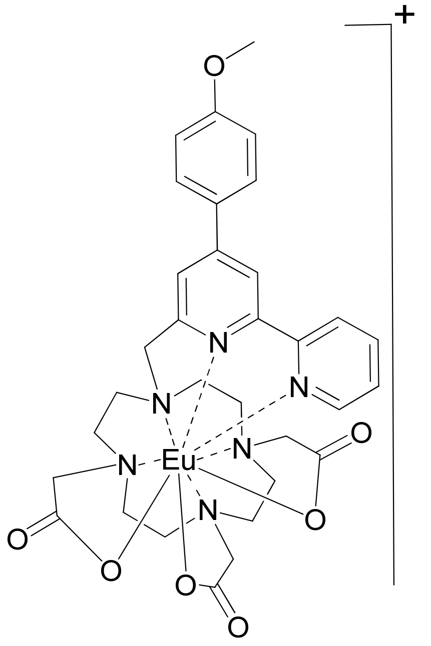

Eu▪4f

18. Figure S17. Mass-spectrum of Tb•4f

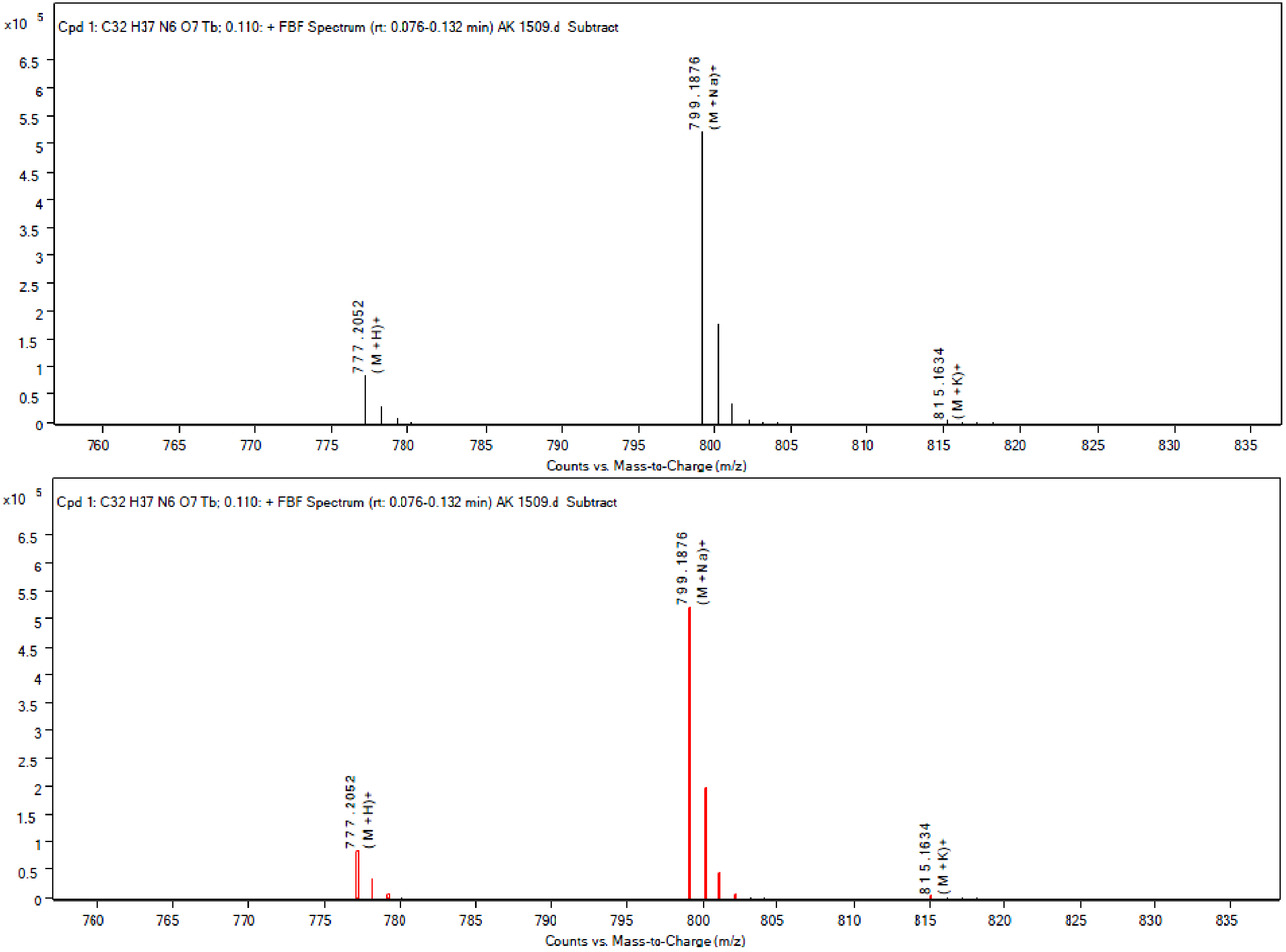

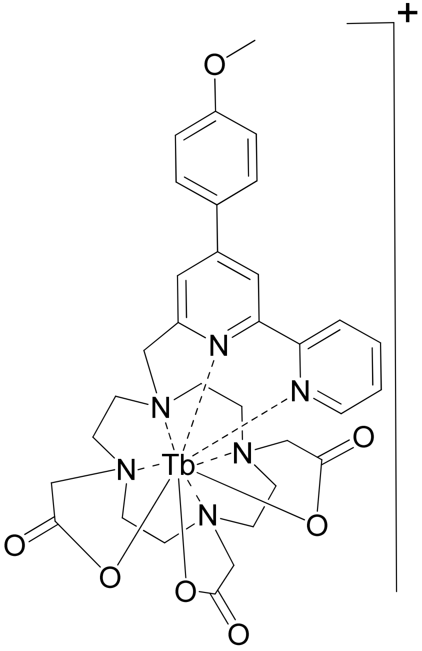

Tb•4f

19. Figure S18. Mass-spectrum of Eu•4g

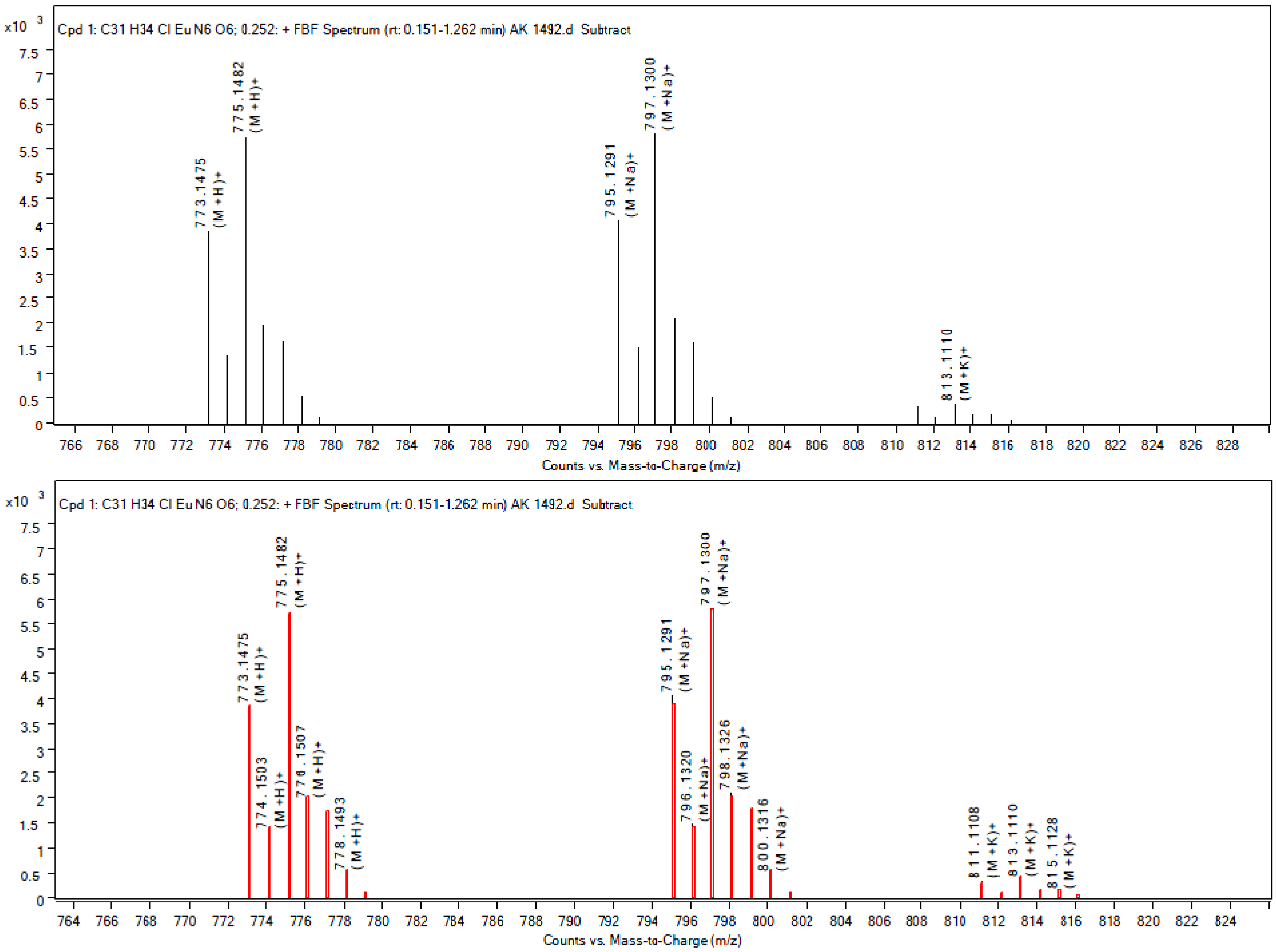

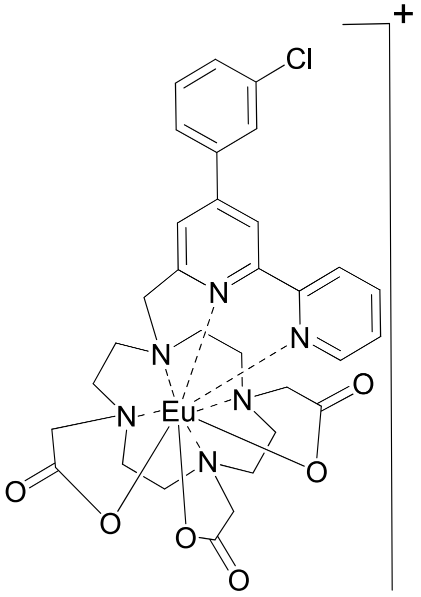

Eu▪4g

20. Figure S19. Mass-spectrum of Tb•4g

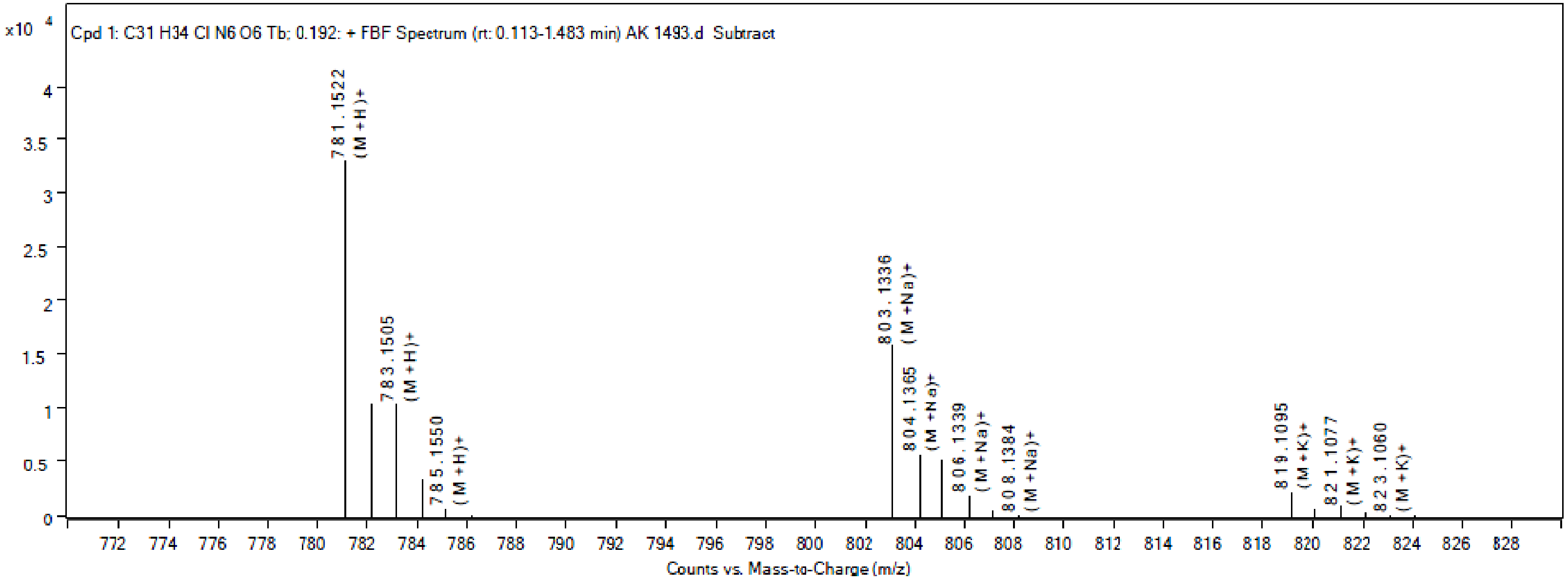

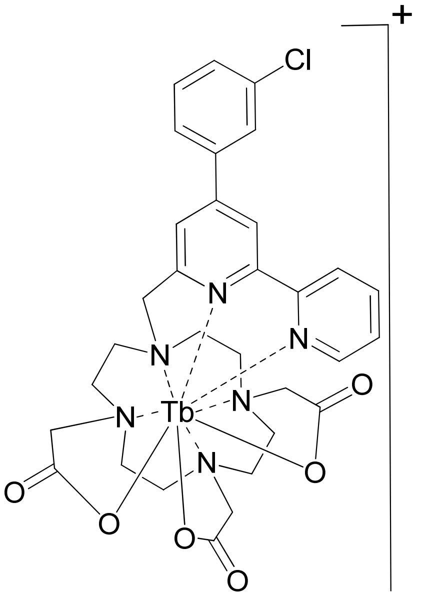

Tb▪4g

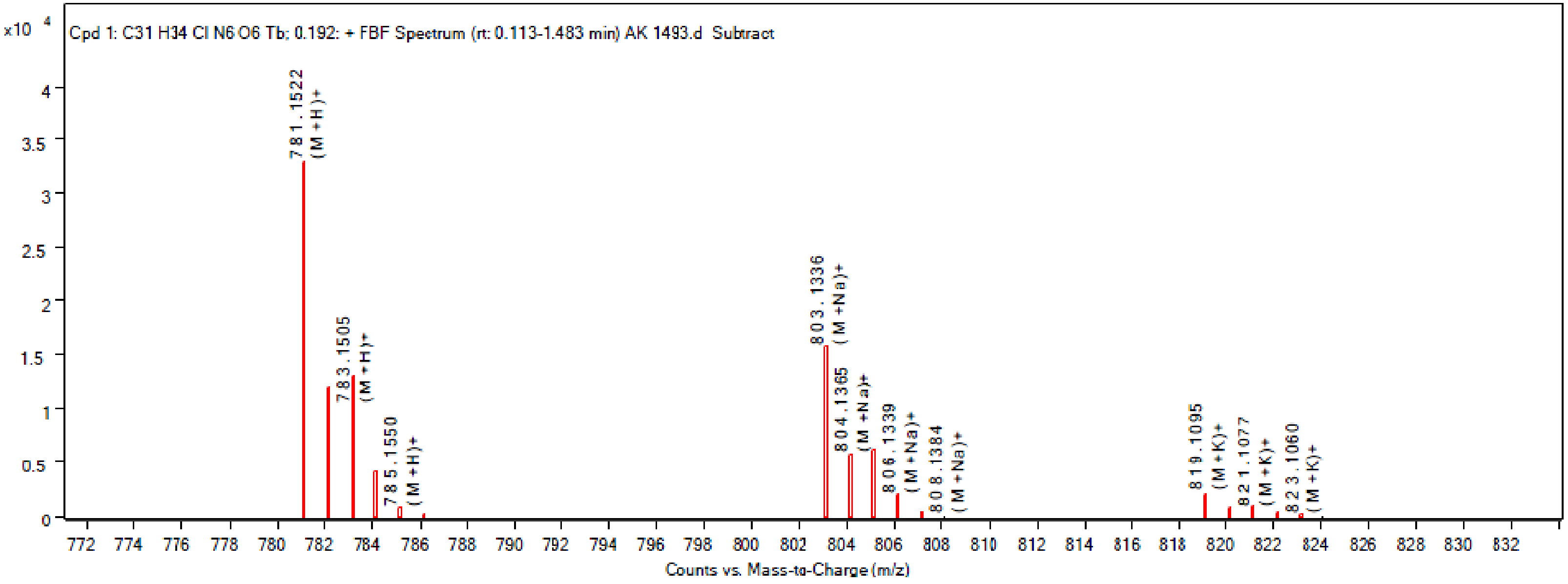

21. Figure S20. Mass-spectrum of Eu•4h

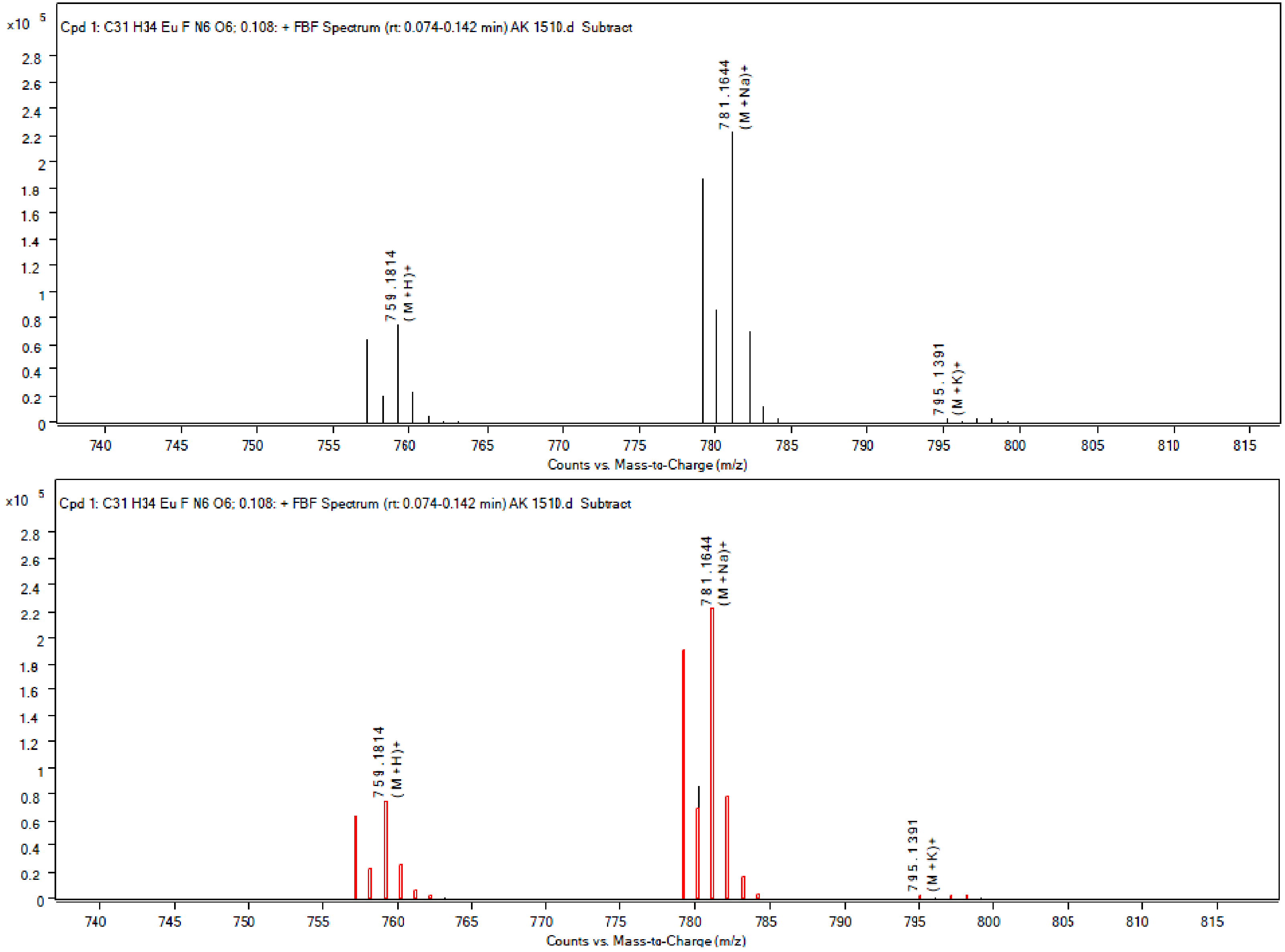

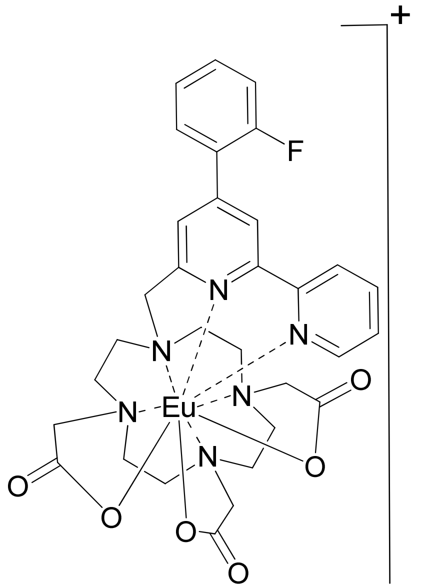

Eu▪4h

22. Figure S21. Mass-spectrum of Tb•4h

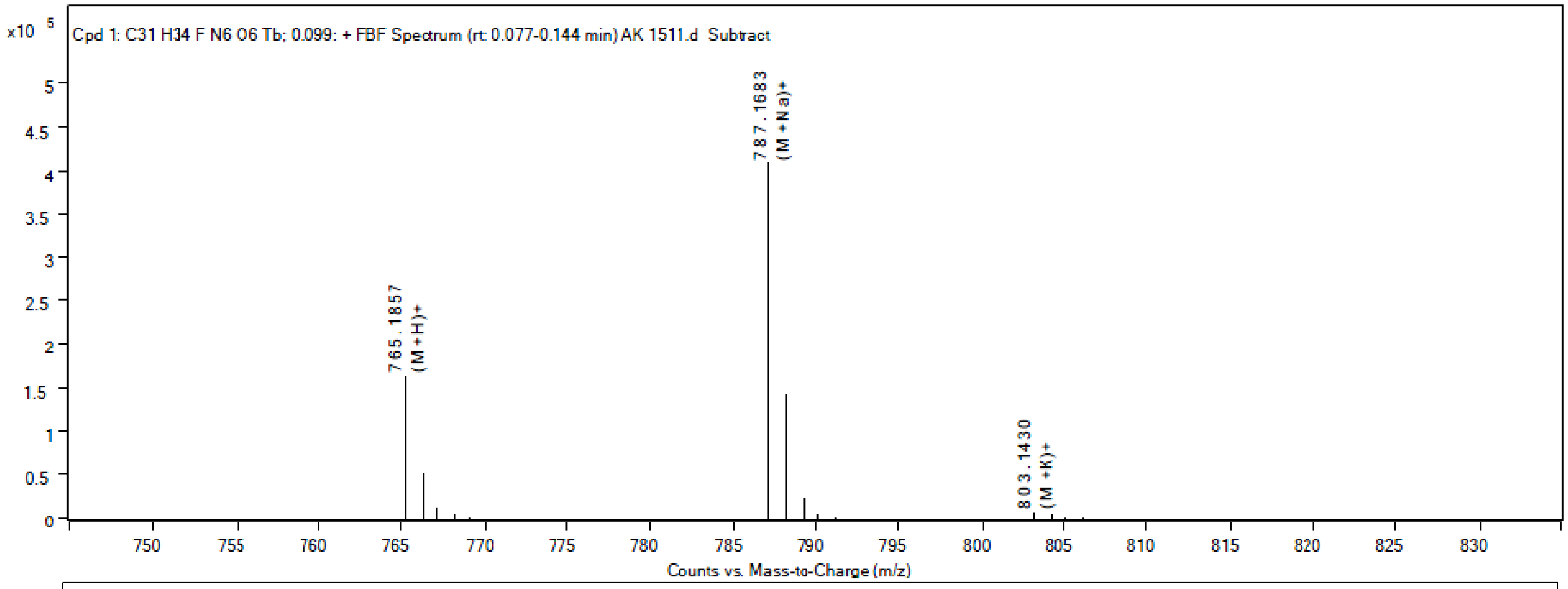

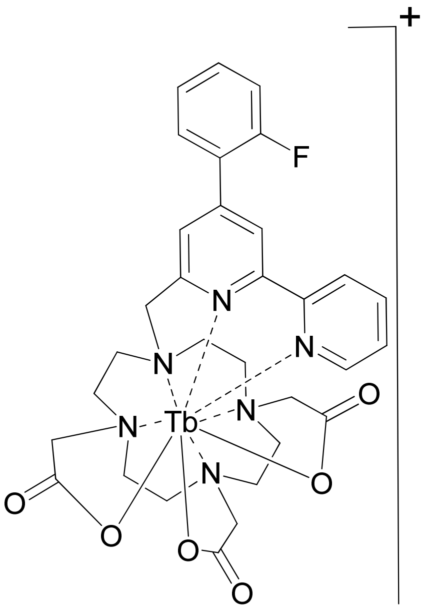

Tb•4h

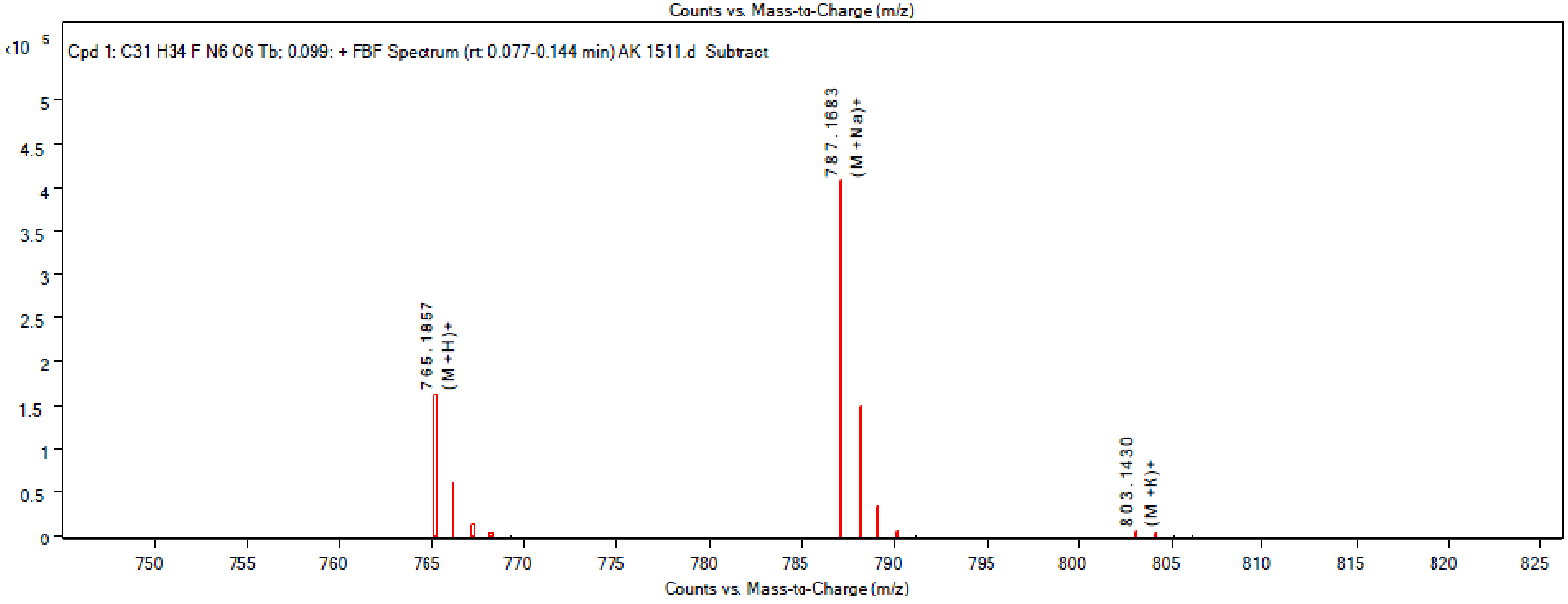

23. Figure S22. Mass-spectrum of Eu•4i

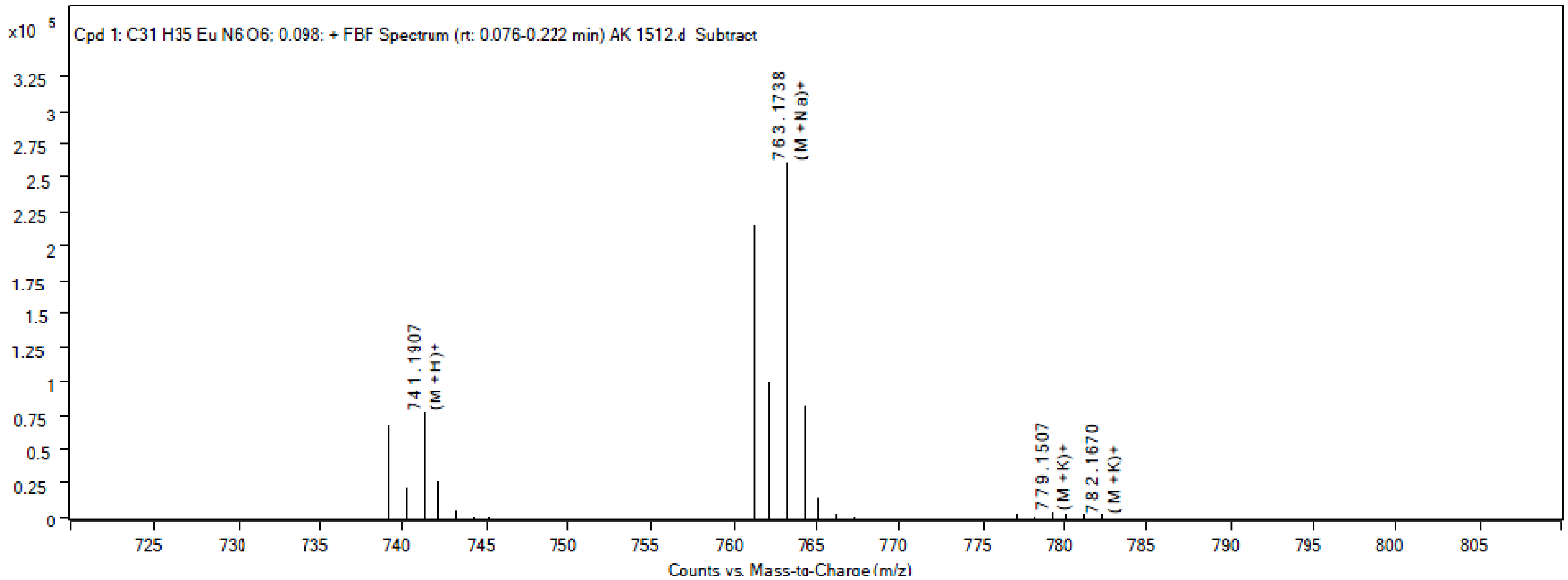

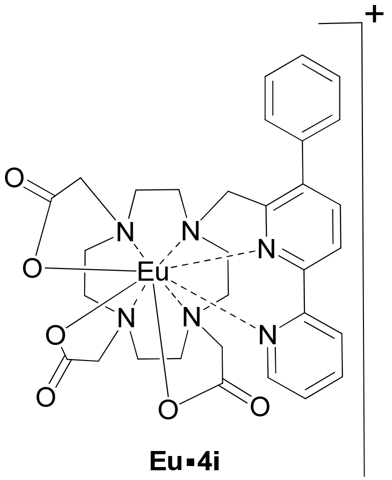

Eu•4i

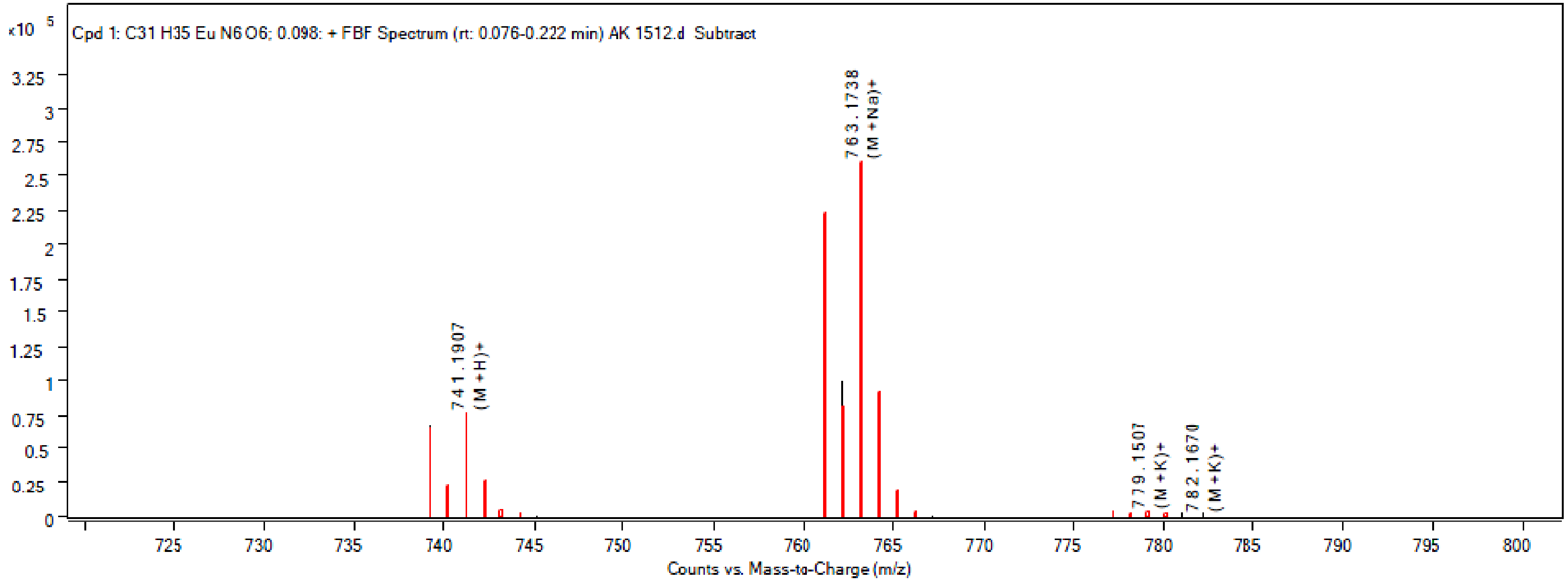

24. Figure S23. Mass-spectrum of Tb•4i

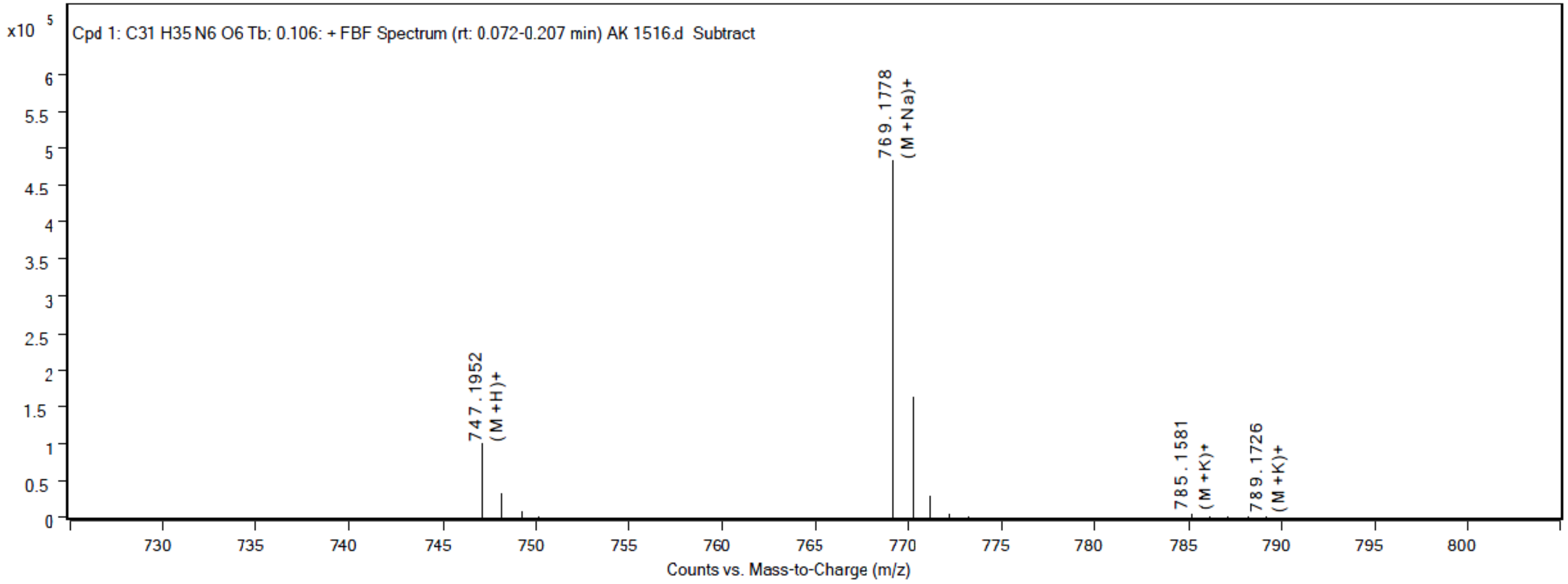

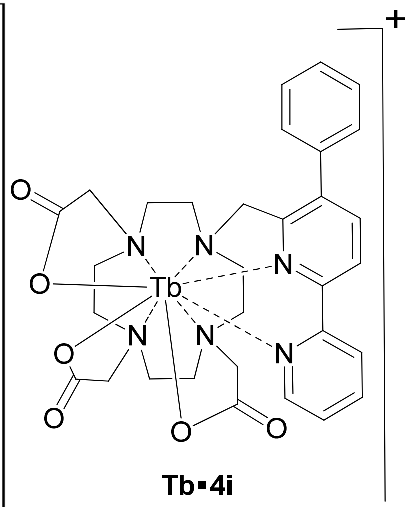

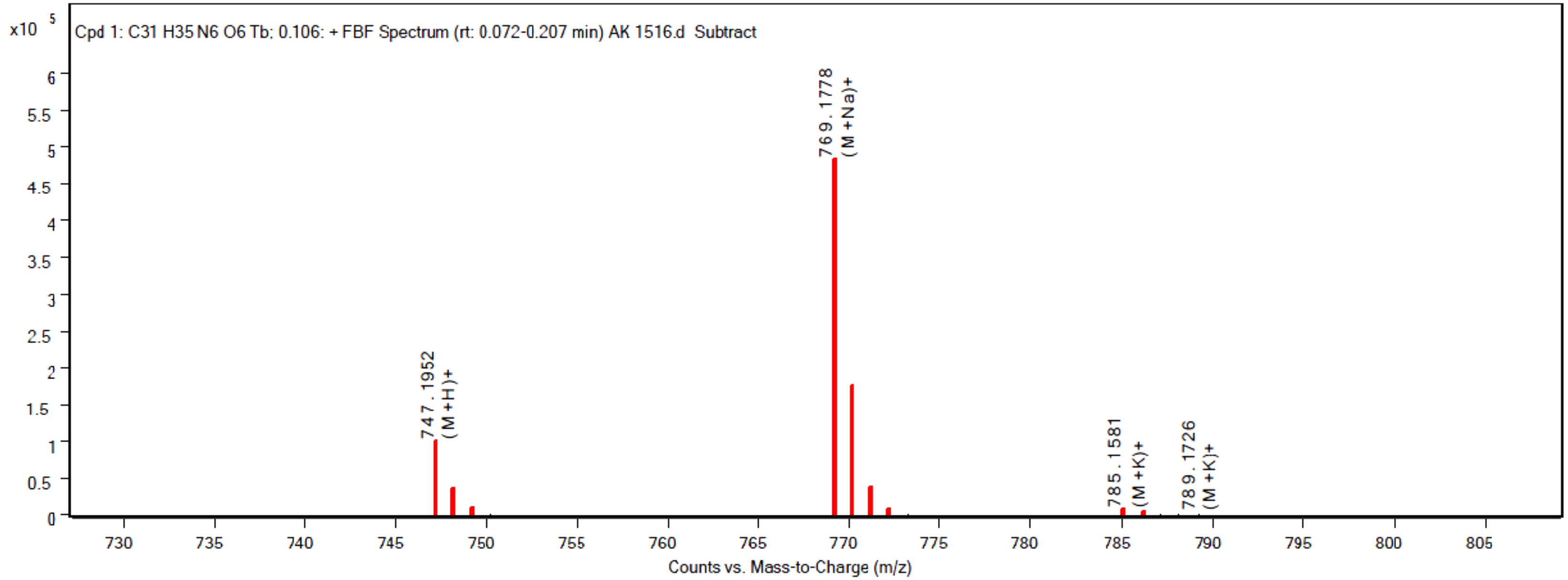

25. Figure S24. Mass-spectrum of Eu•5b

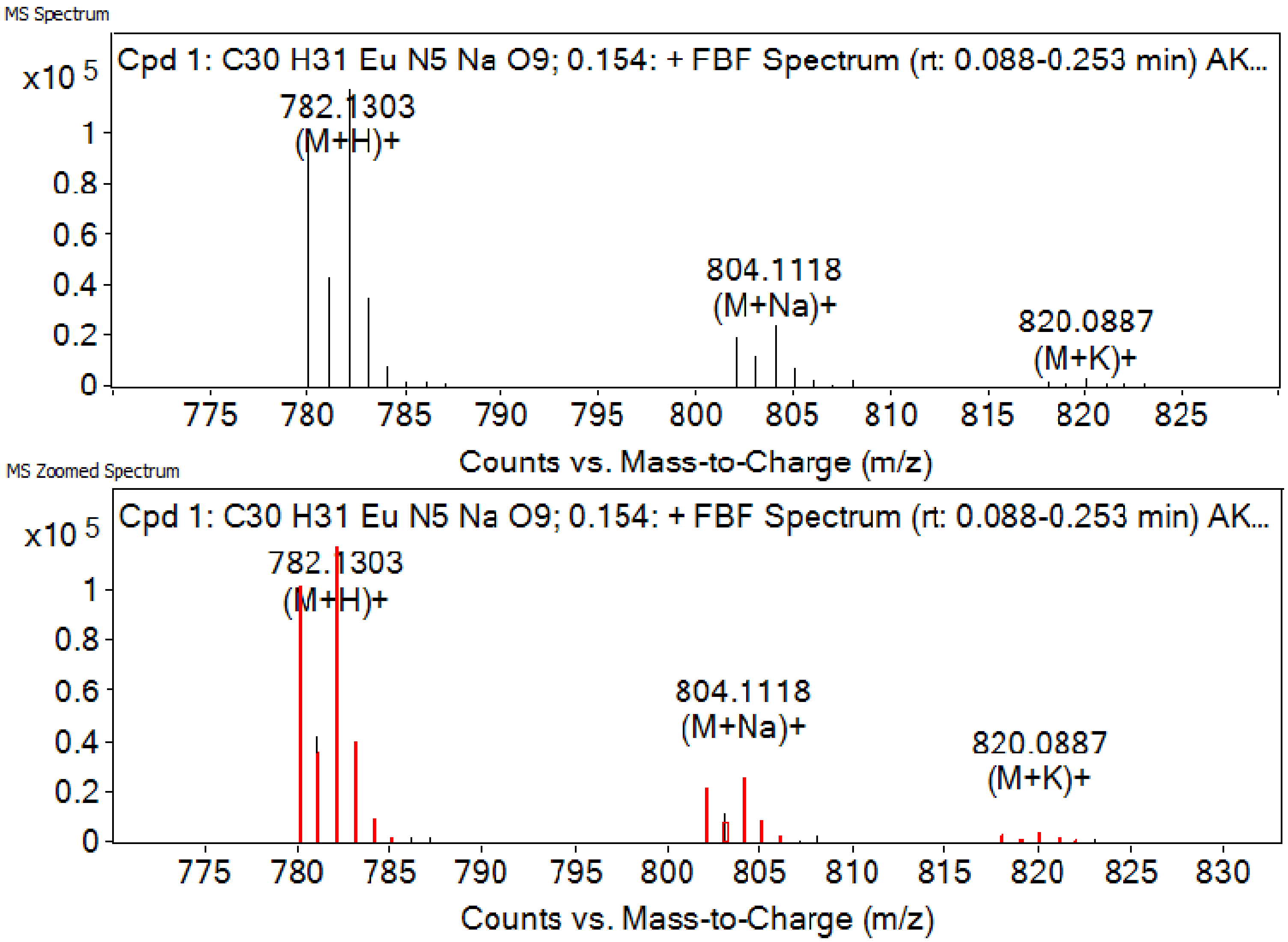

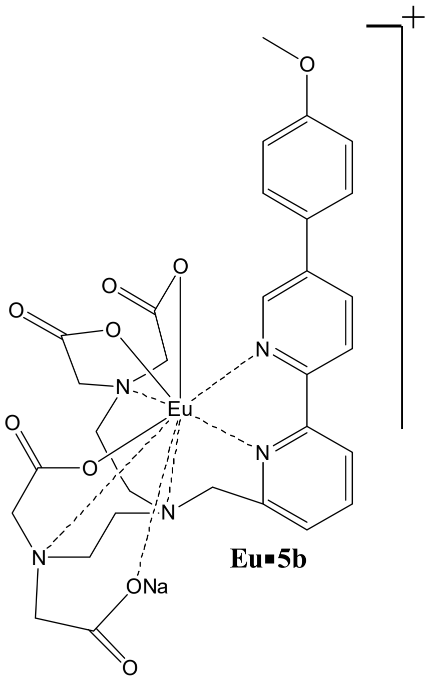

Eu▪5b

26. Figure S25. Mass-spectrum of Eu•5c

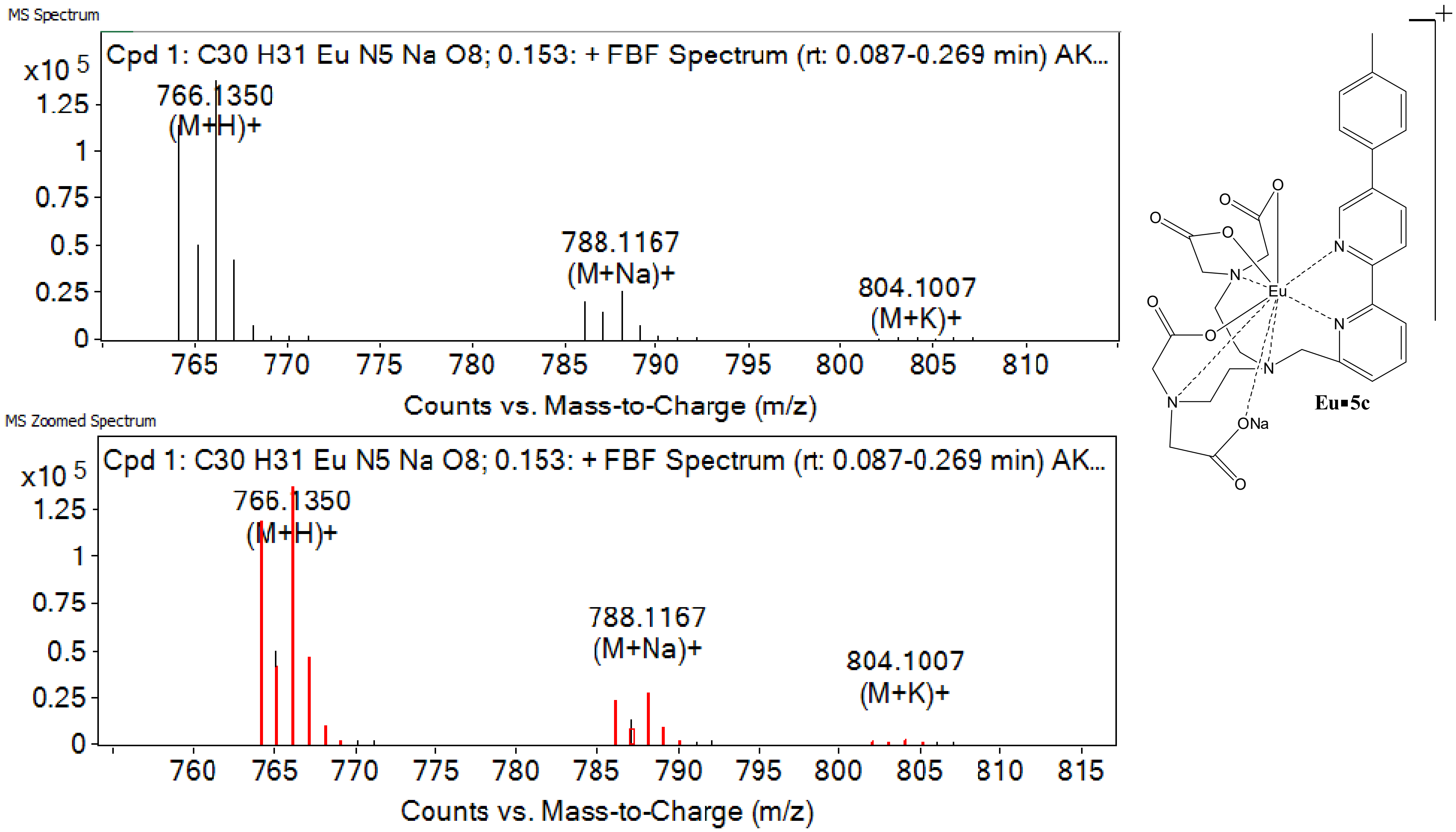

27. Figure S26. Mass-spectrum of Eu•5d

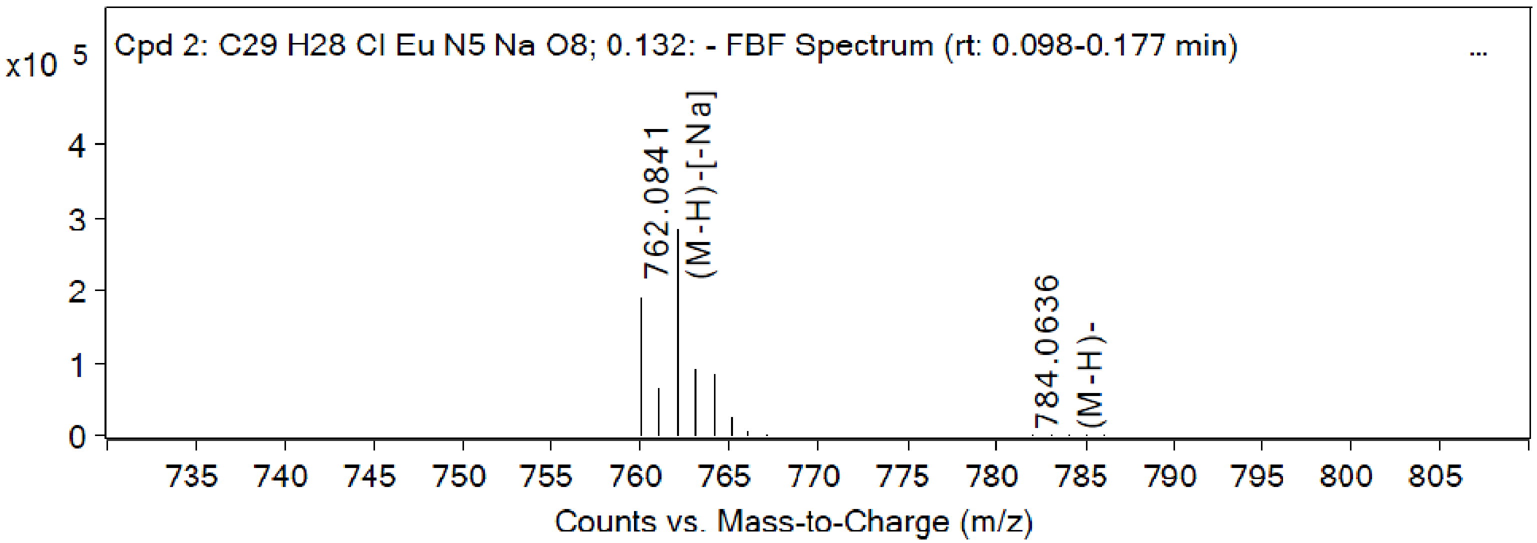

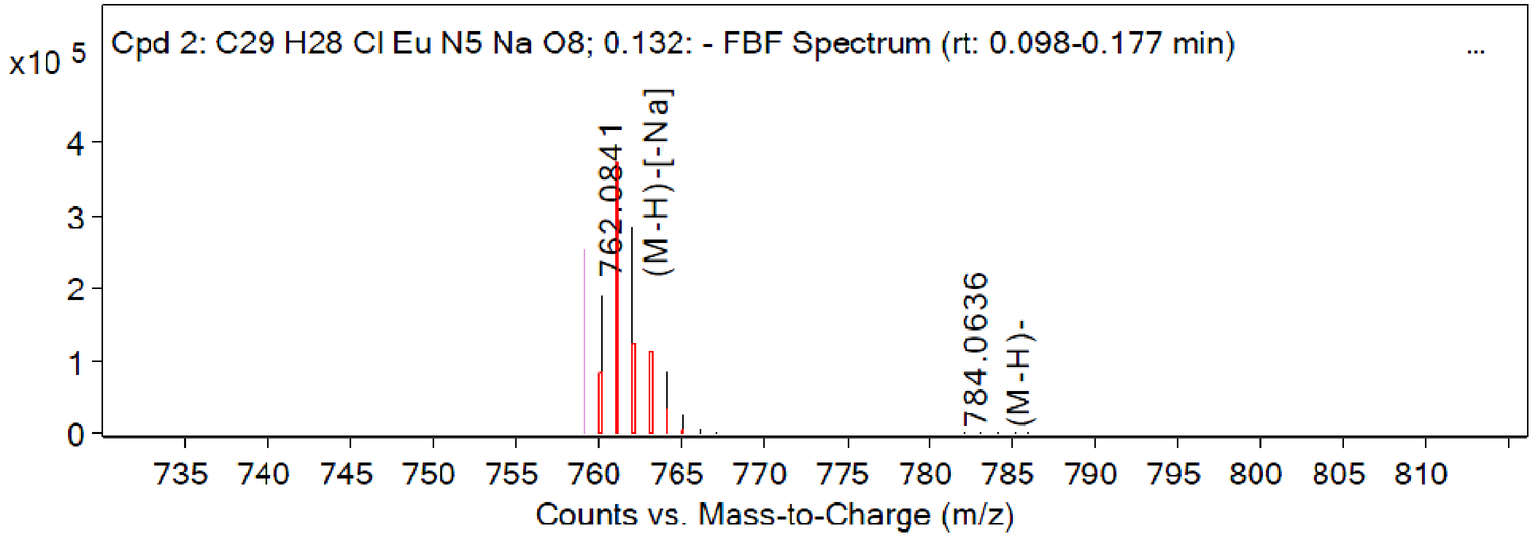

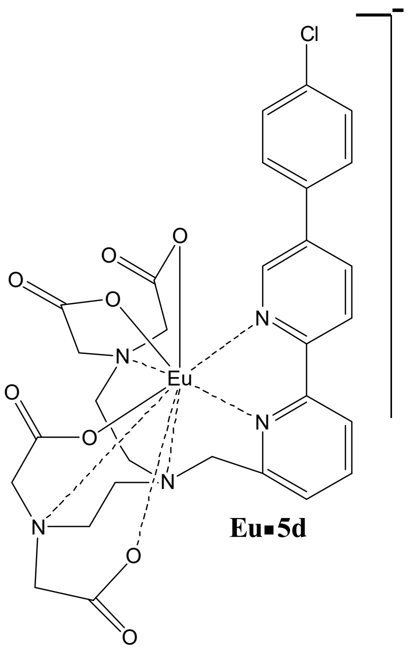

Eu▪5d

28. Figure S27. Mass-spectrum of Eu•5e

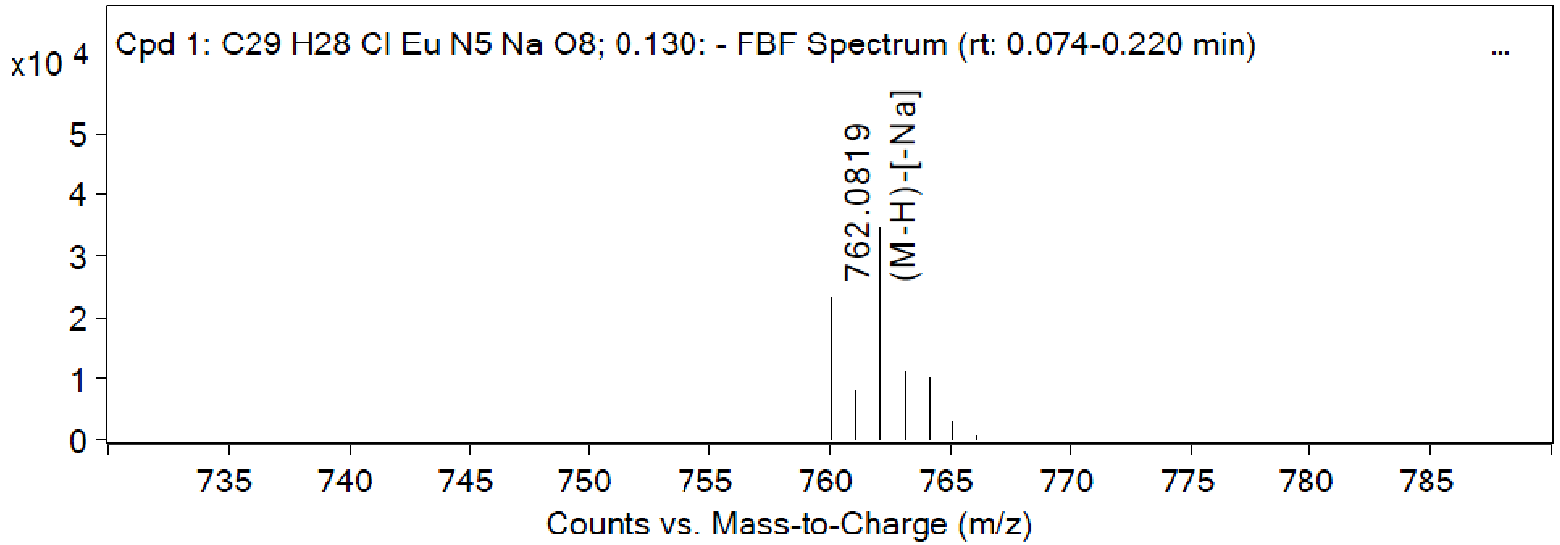

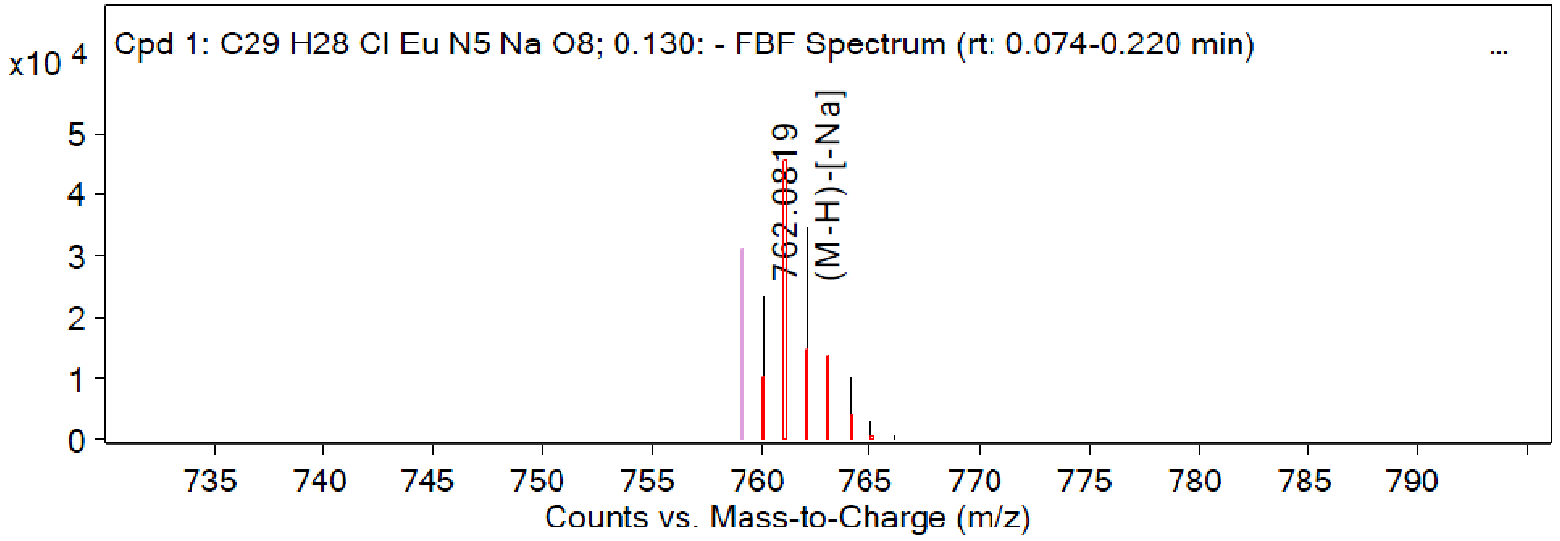

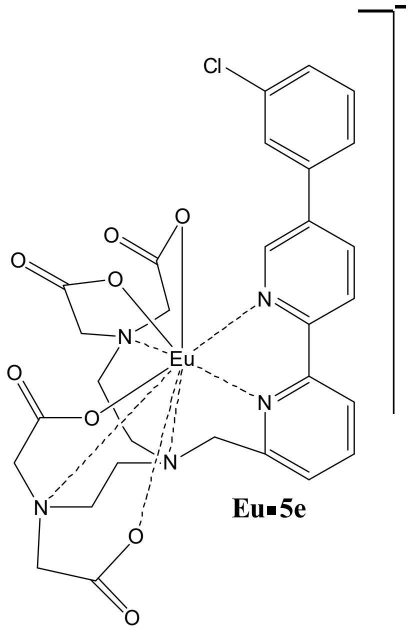

Eu•5e

29. Figure S28. Absorption spectra of Eu•4c and Eu•4d in H_2O

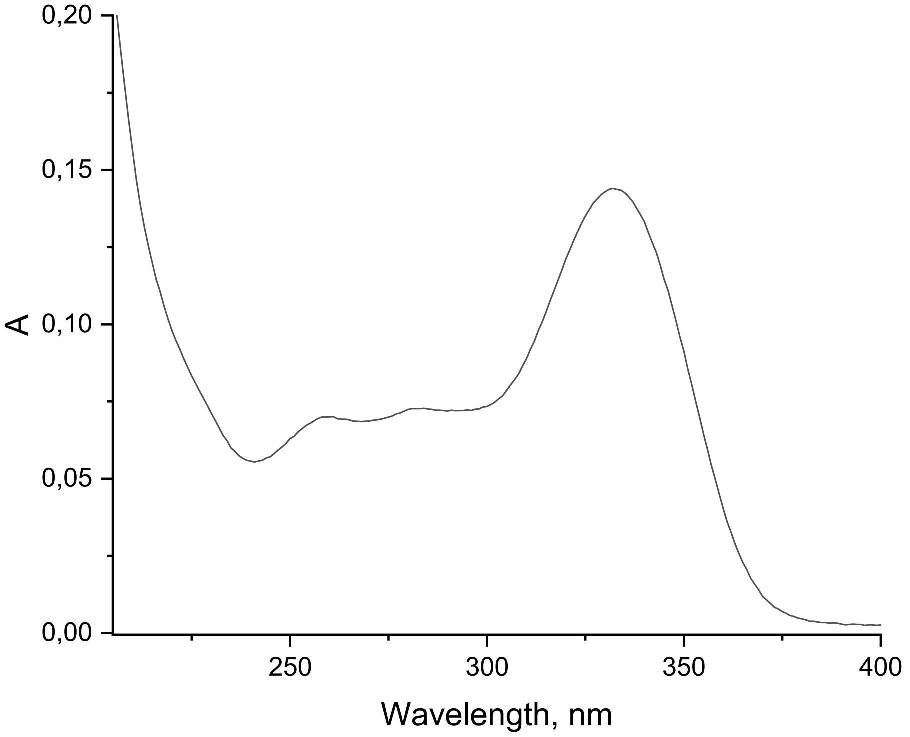

Figure S28a. Absorption spectrum of **Eu•4c** in water at room temperature.

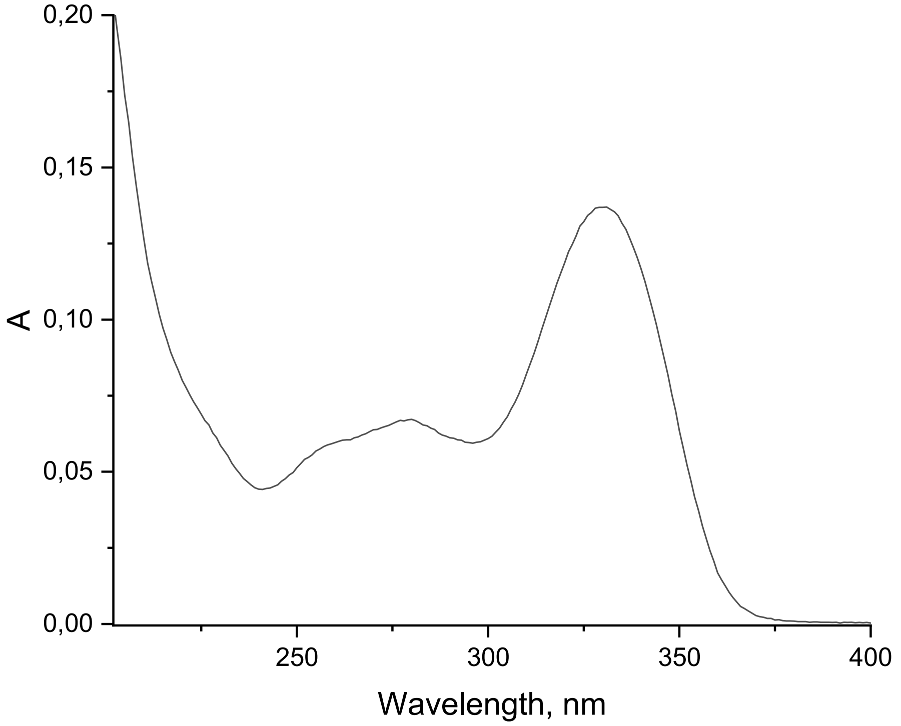

Figure S28b. Absorption spectrum of **Eu•4d** in water at room temperature.

30. Figure S29. Absorption spectra of Eu•4e and Eu•4f in H_2O

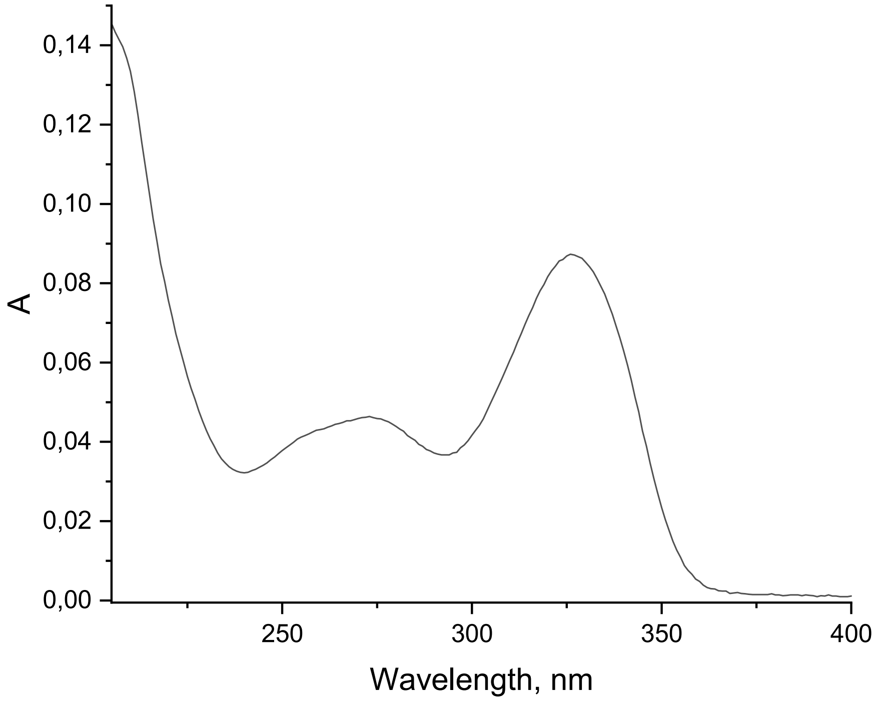

Figure S29a. Absorption spectrum of **Eu•4e** in water at room temperature.

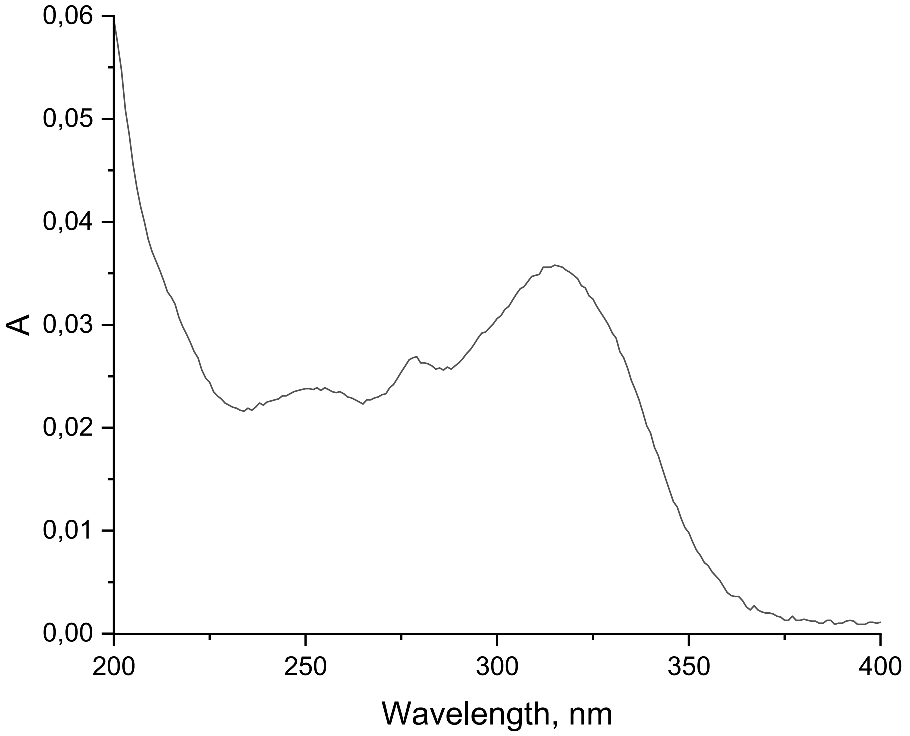

Figure S29b. Absorption spectrum of **Eu•4f** in water at room temperature.

31. Figure S30. Absorption spectra of Tb•4f and Eu•4g in H_2O

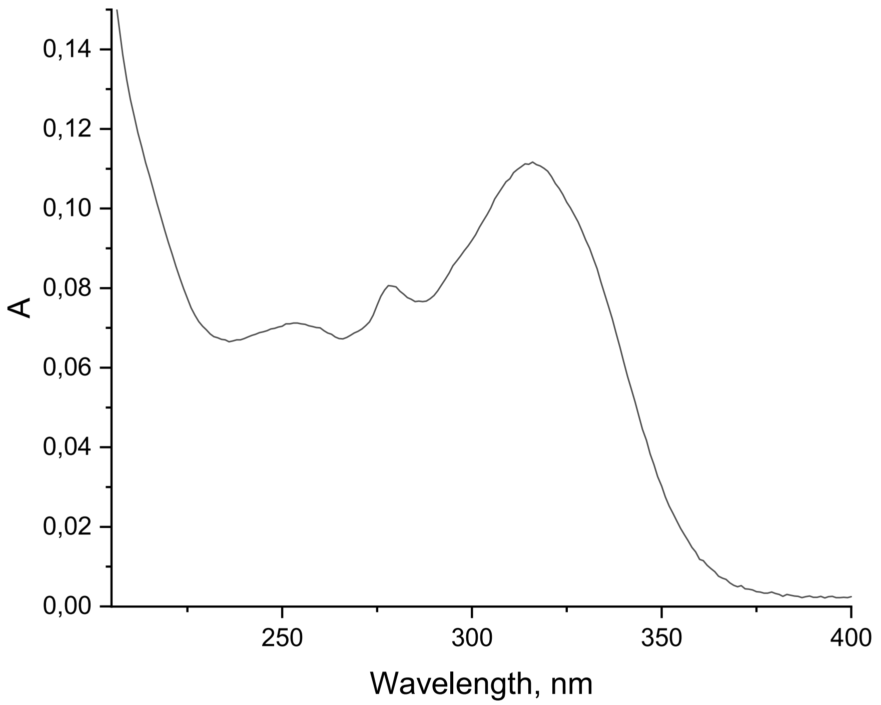

Figure S30a. Absorption spectrum of **Tb•4f** in water at room temperature.

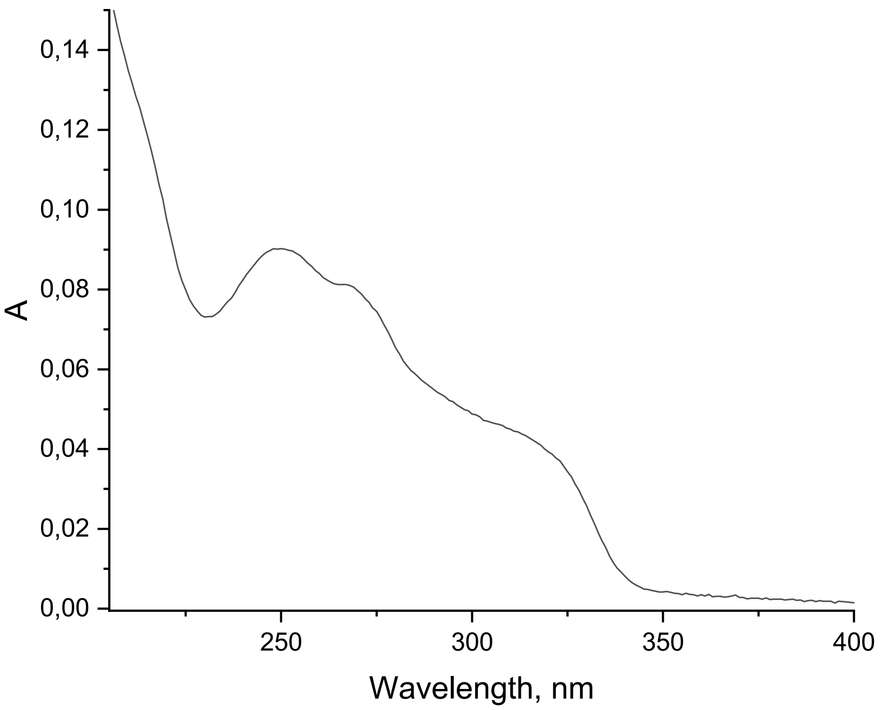

Figure S30b. Absorption spectrum of **Eu•4g** in water at room temperature.

32. Figure S31. Absorption spectra of Tb•4g and Eu•4h in H_2O

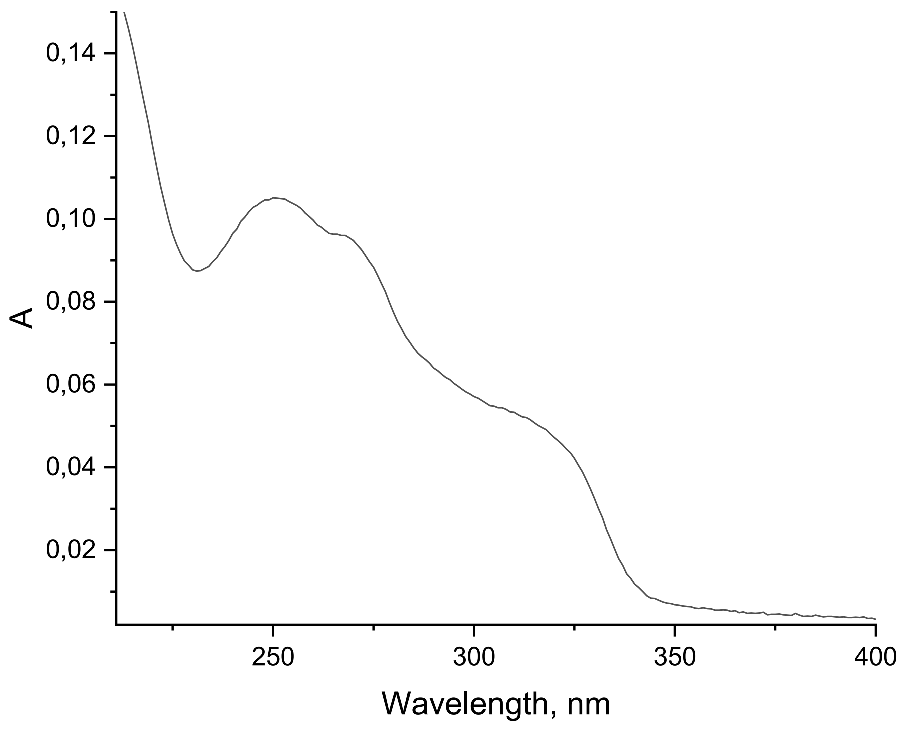

Figure S31a. Absorption spectrum of **Tb•4g** in water at room temperature.

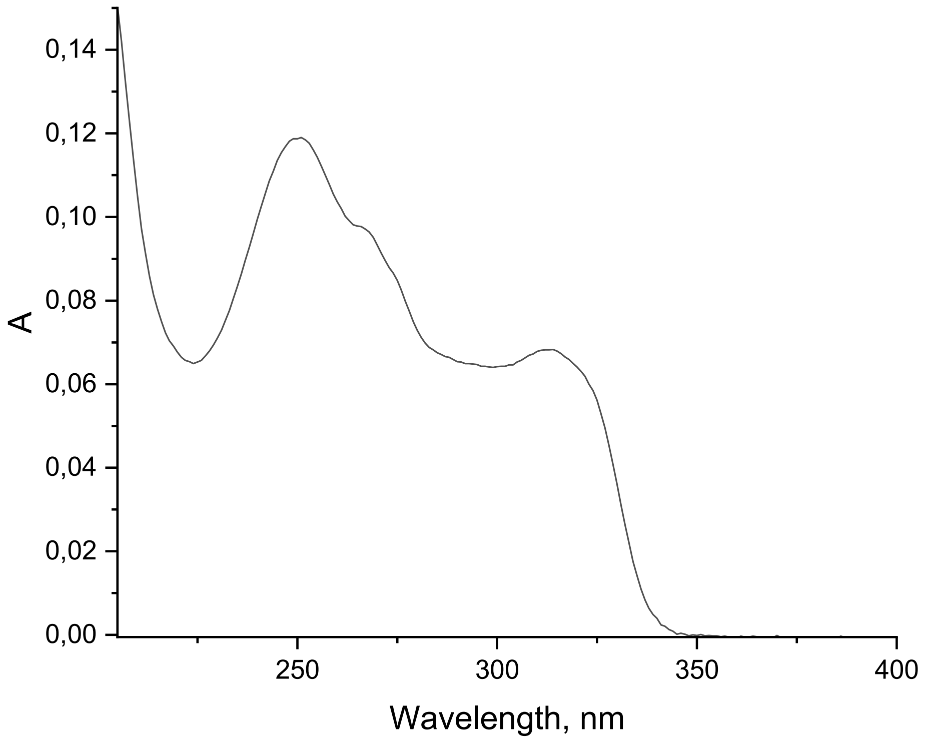

Figure S31b. Absorption spectrum of **Eu•4h** in water at room temperature.

33. Figure S32. Absorption spectra of Tb•4h and Eu•4i in H_2O

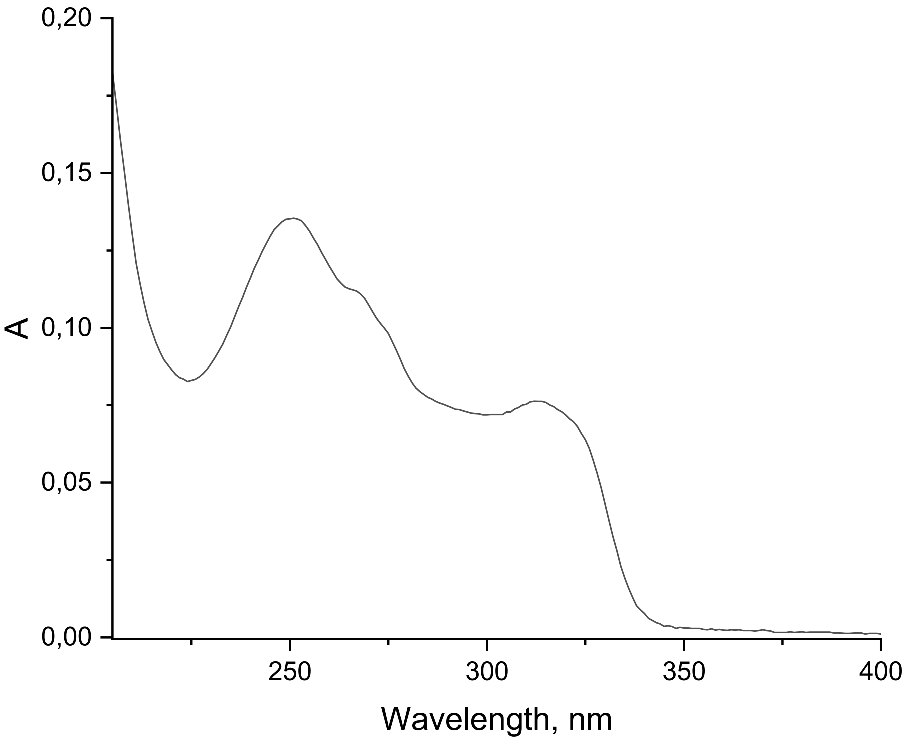

Figure S32a. Absorption spectrum of **Tb•4h** in water at room temperature.

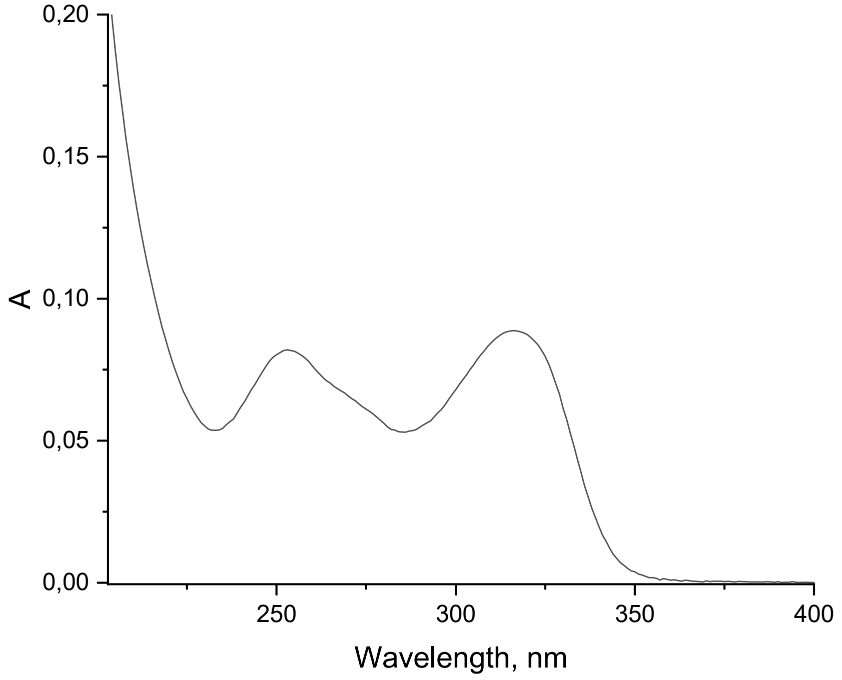

Figure S32b. Absorption spectrum of **Eu•4i** in water at room temperature.

34. Figure S33. Absorption spectra of Tb•4i and Eu•5b in H_2O

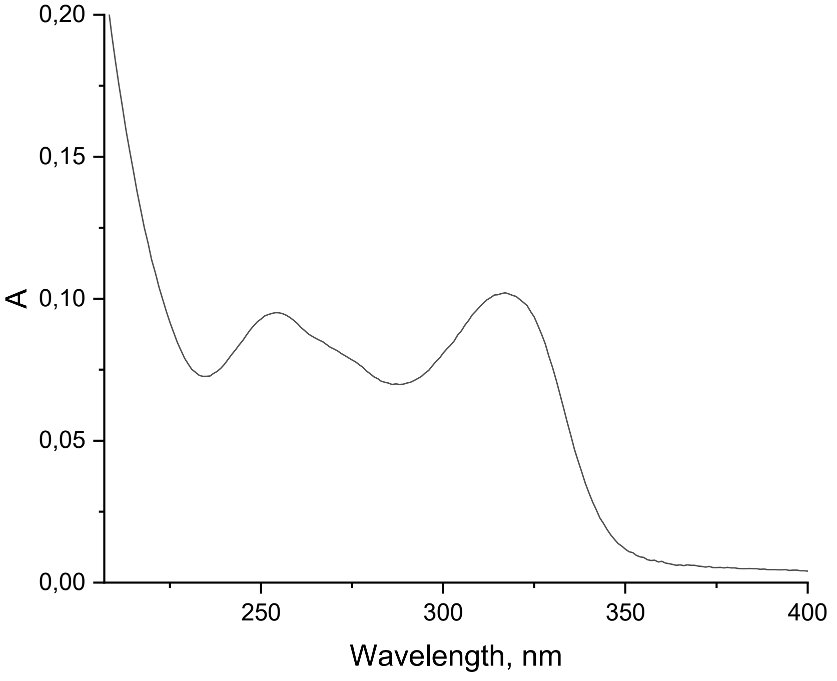

Figure S33a. Absorption spectrum of **Tb•4i** in water at room temperature.

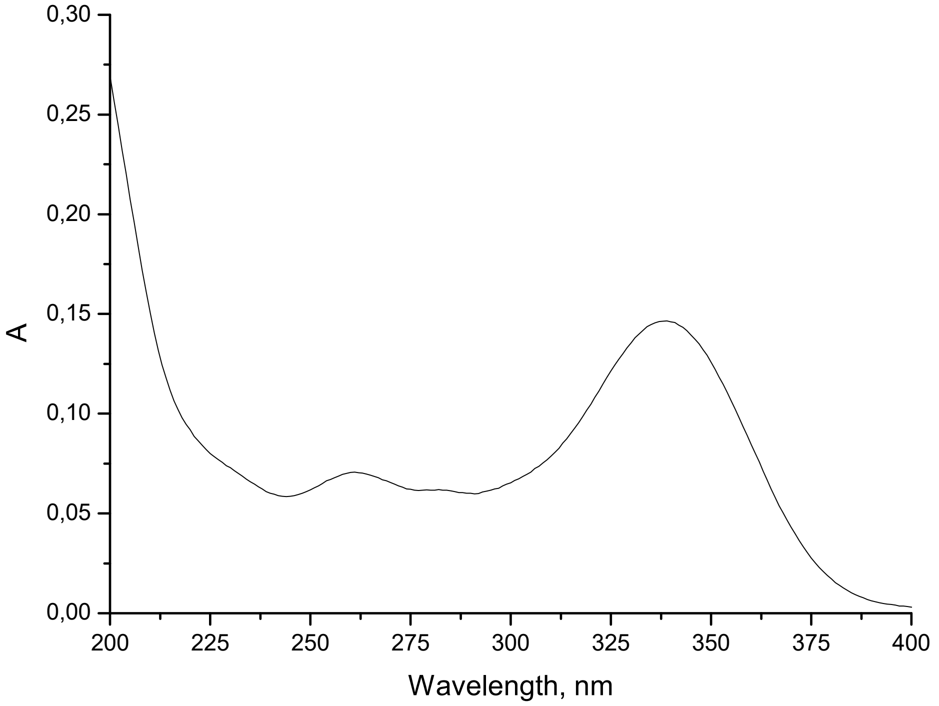

Figure S33b. Absorption spectrum of **Eu•5b** in water at room temperature.

35. Figure S34. Absorption spectra of Eu•5c and Eu•5d in H_2O

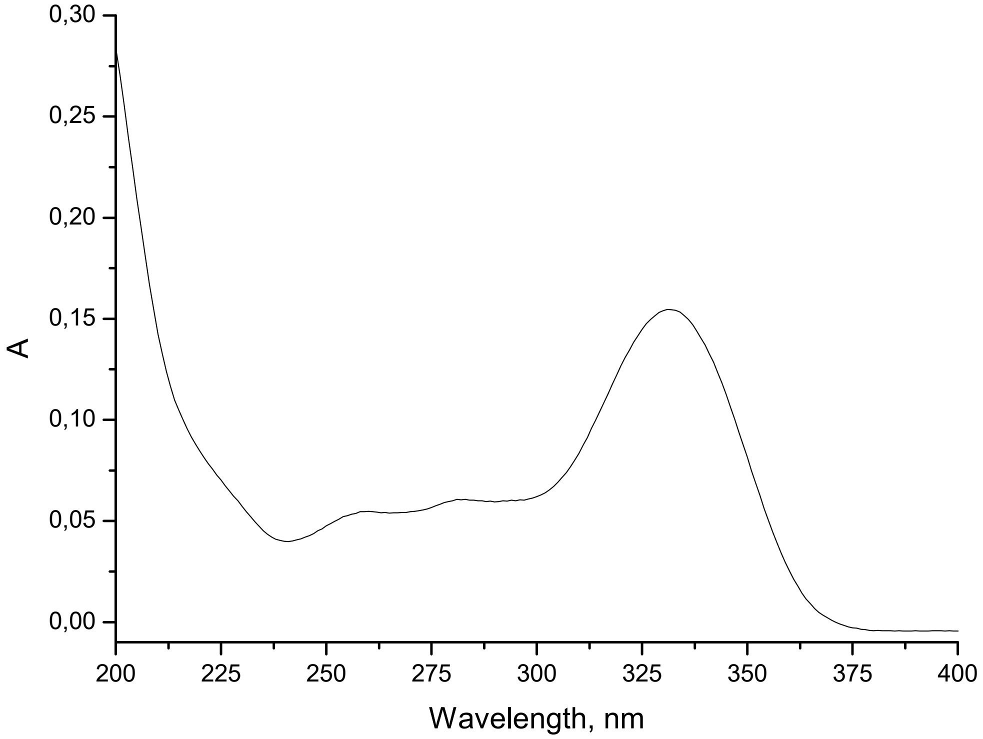

Figure S34a. Absorption spectrum of **Eu•5c** in water at room temperature.

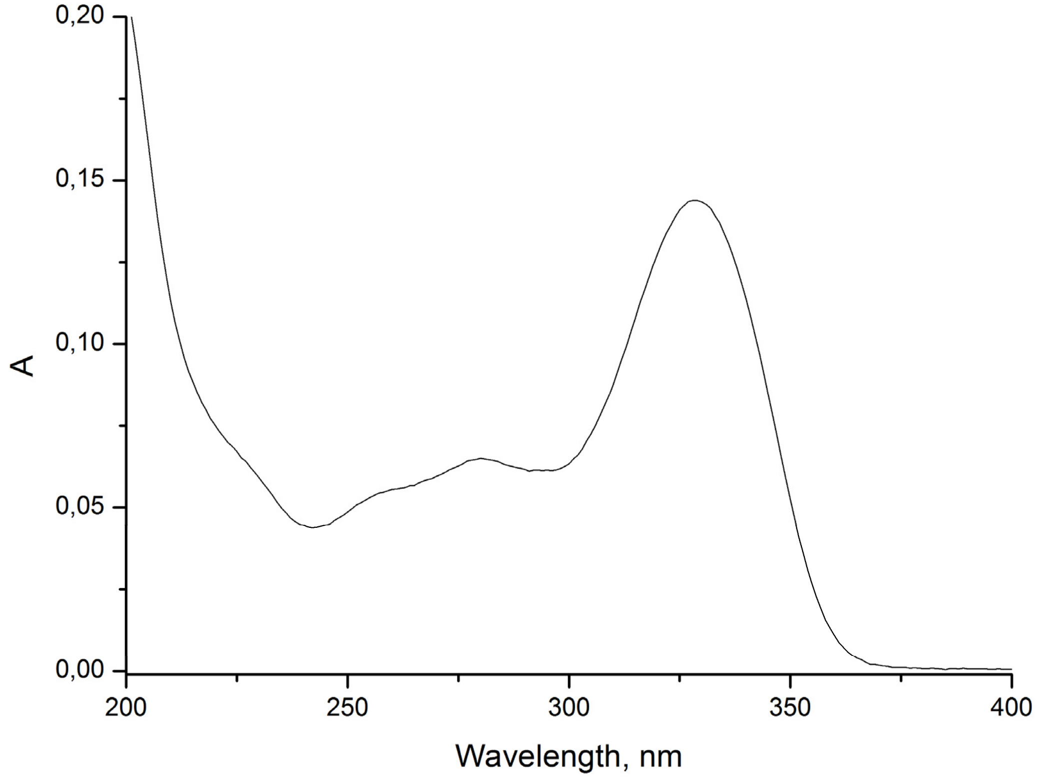

Figure S34b. Absorption spectrum of **Eu•5d** in water at room temperature.

36. Figure S35. Absorption spectrum of Eu•5e and in H_2O

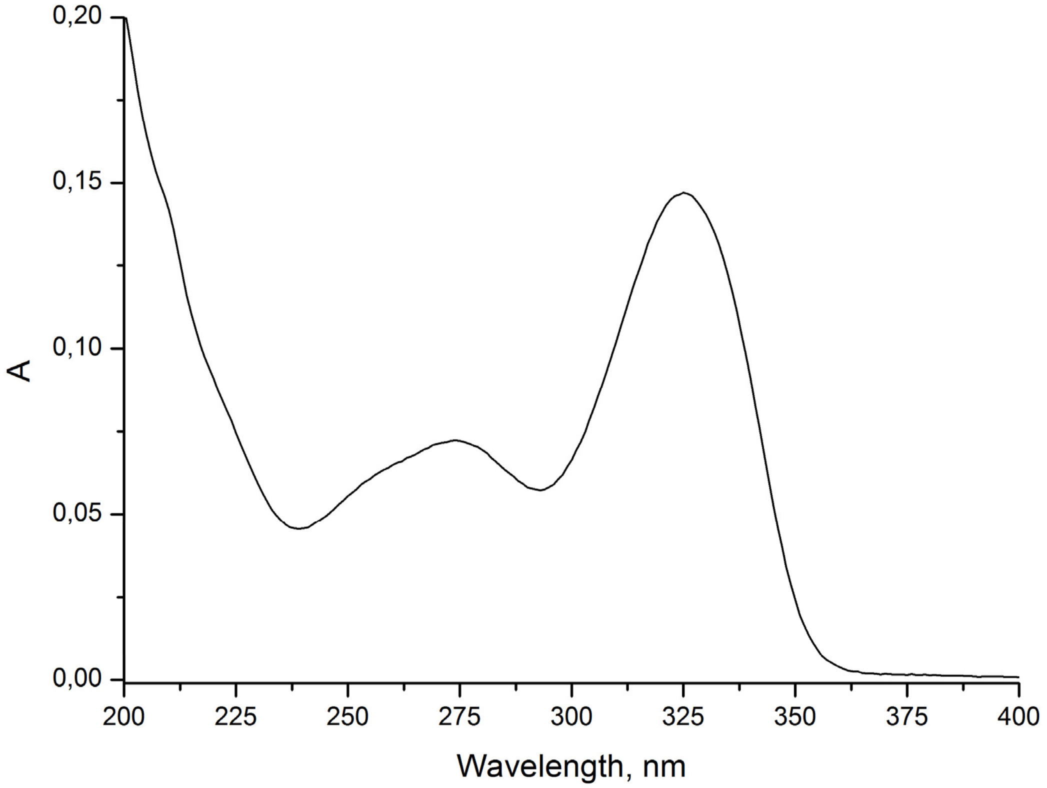

Figure S35a. Absorption spectrum of **Eu•5e** in water at room temperature.

37. Figure S36. Luminescence spectrum and decay curves of Eu•5b

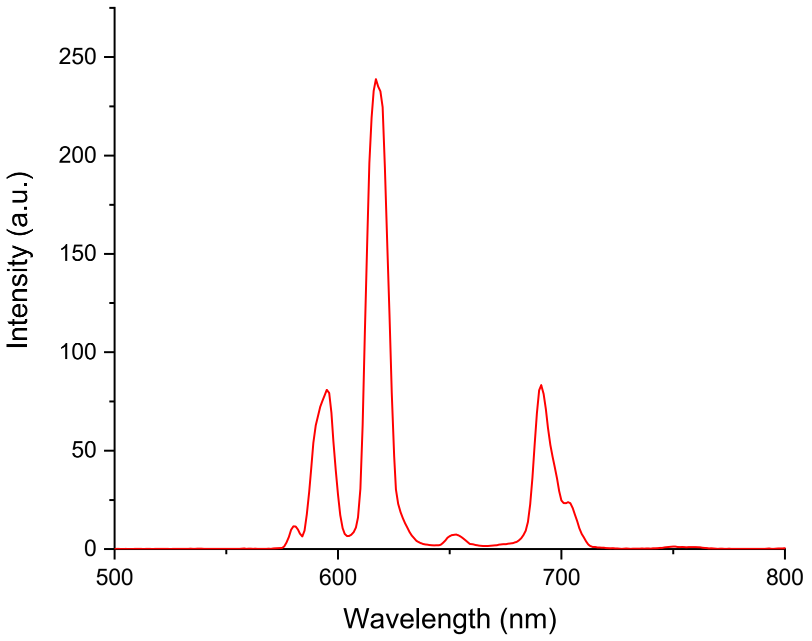

Figure S36a. Europium cation luminescence spectrum of **Eu•5b** in water at room temperature

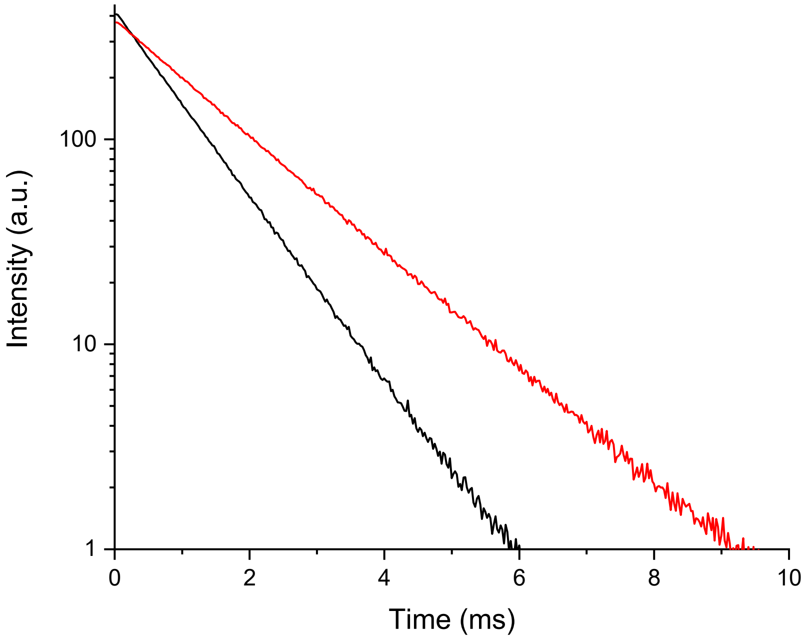

Figure S36b. Decay curves of luminescence of **Eu•5b** in water (black line) and D_2O (red line) at room temperature

38. Figure S37. Luminescence spectrum and decay curves of Eu•5c

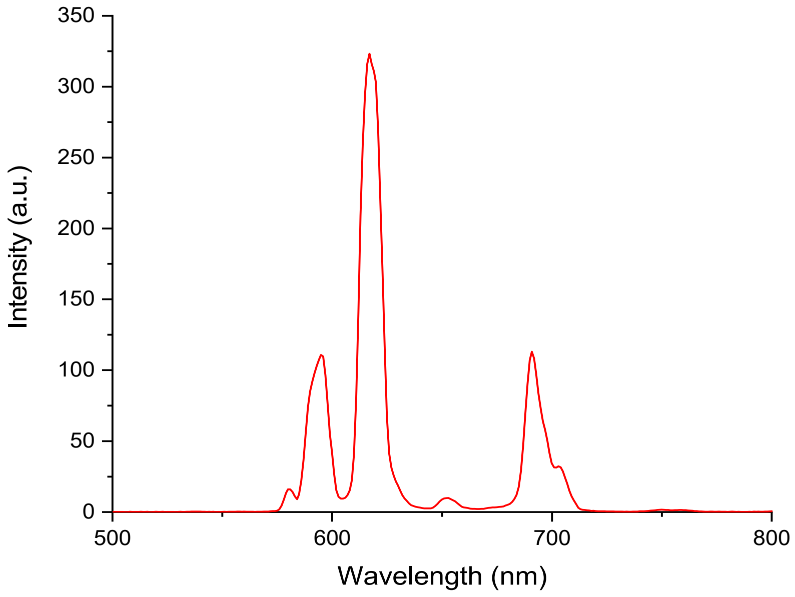

Figure S37a. Europium cation luminescence spectrum of **Eu•5c** in water at room temperature

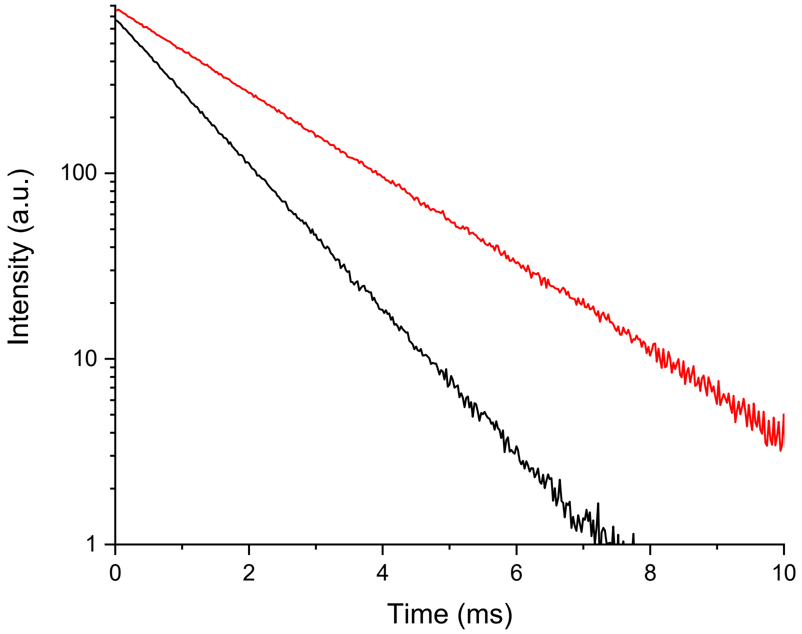

Figure S37b. Decay curves of luminescence of **Eu•5c** in water (black line) and D_2O (red line) at room temperature

39. Figure S38. Luminescence spectrum and decay curves of Eu•4c

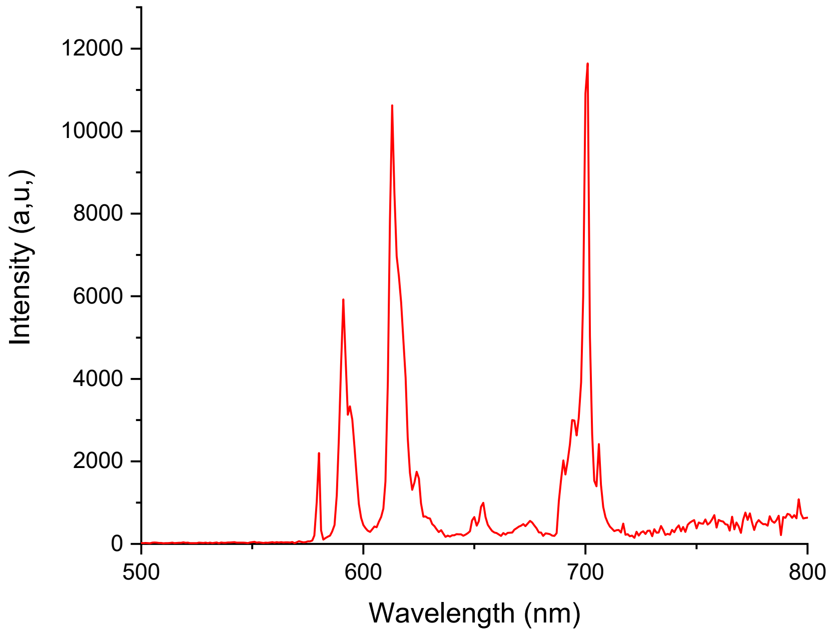

Figure S38a. Europium cation luminescence spectrum of **Eu•4c** in water at room temperature

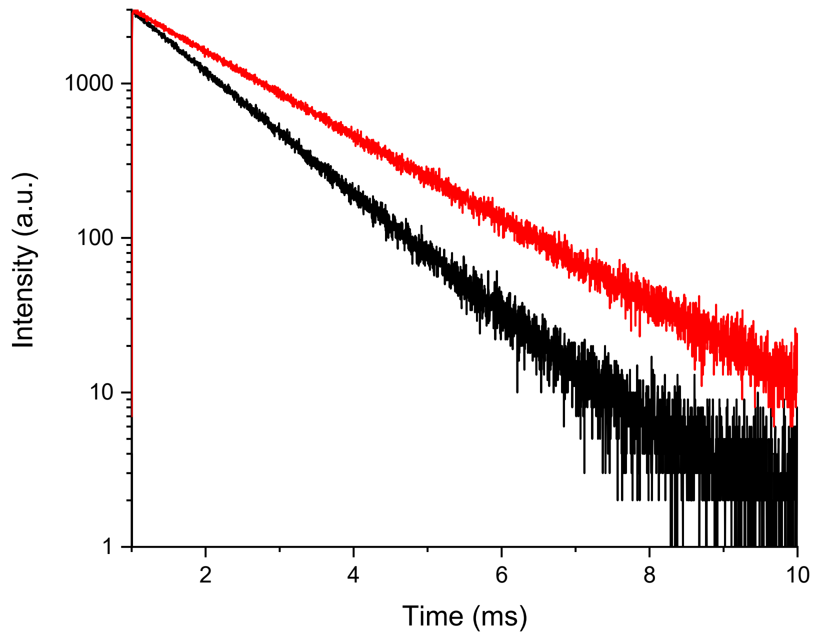

Figure S38b. Decay curves of luminescence of **Eu•4c** in water (black line) and D_2O (red line) at room temperature

40. Figure S39. Luminescence spectrum and decay curves of Eu•5d

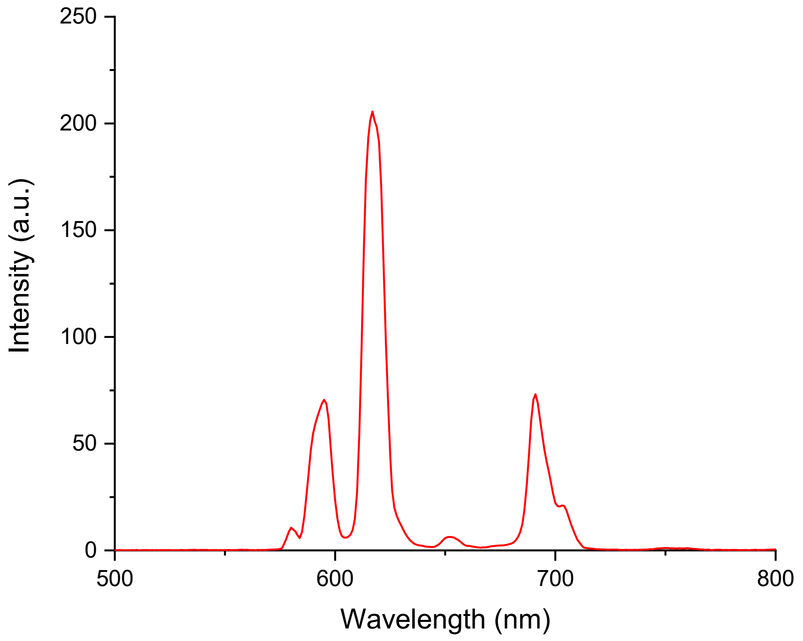

Figure S39a. Europium cation luminescence spectrum of **Eu•5d** in water at room temperature

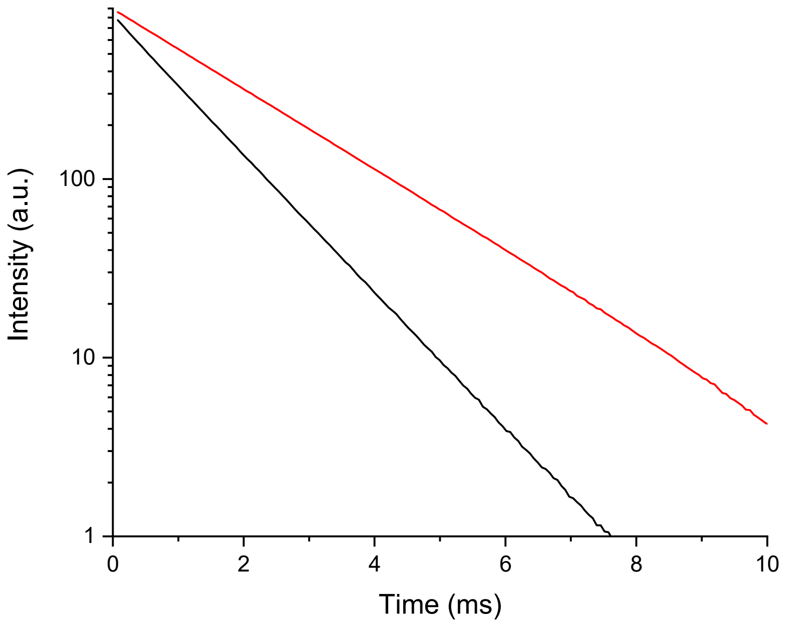

Figure S39b. Decay curves of luminescence of **Eu•5d** in water (black line) and D_2O (red line) at room temperature

41. Figure S40. Luminescence spectrum and decay curves of Eu•4d

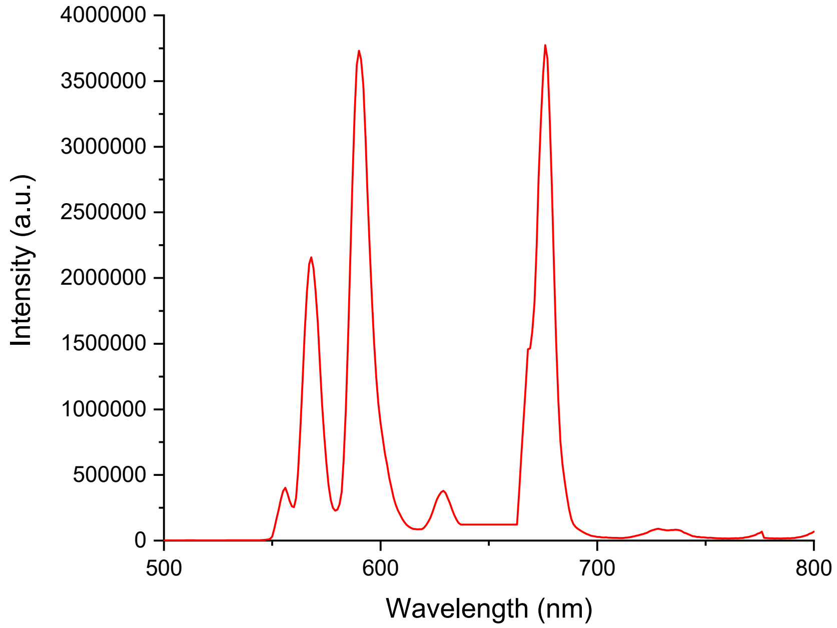

Figure S40a. Europium cation luminescence spectrum of **Eu•4d** in water at room temperature

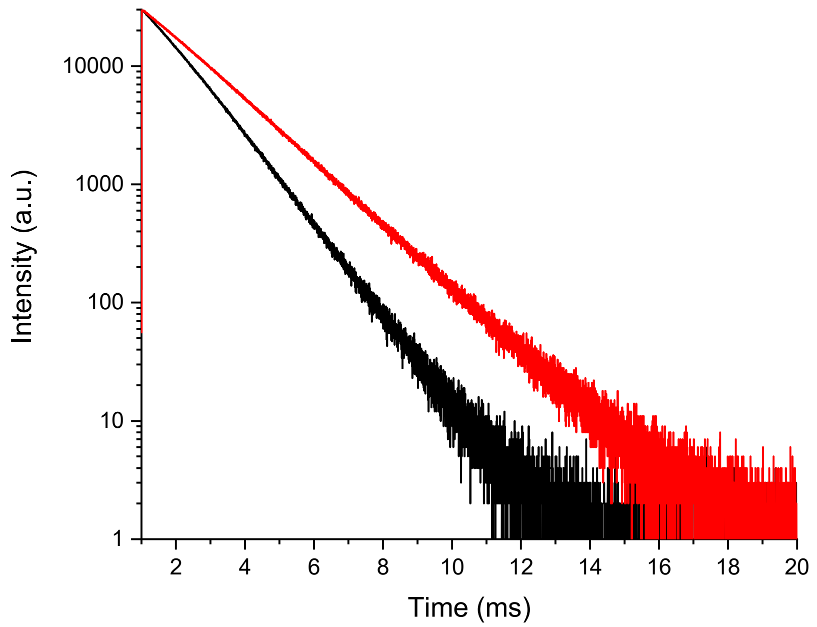

Figure S40b. Decay curves of luminescence of **Eu•4d** in water (black line) and D_2O (red line) at room temperature

42. Figure S41. Luminescence spectrum and decay curves of Eu•5e

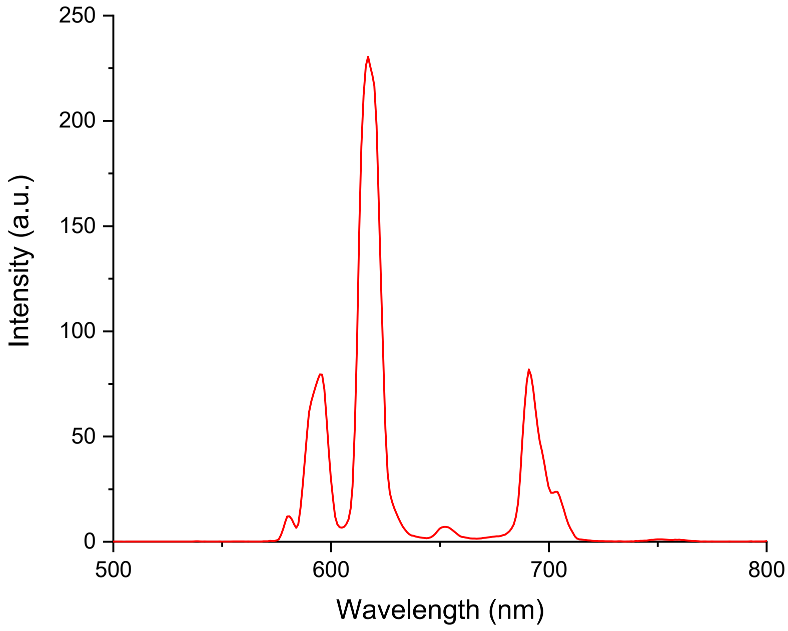

Figure S41a. Europium cation luminescence spectrum of **Eu•5e** in water at room temperature

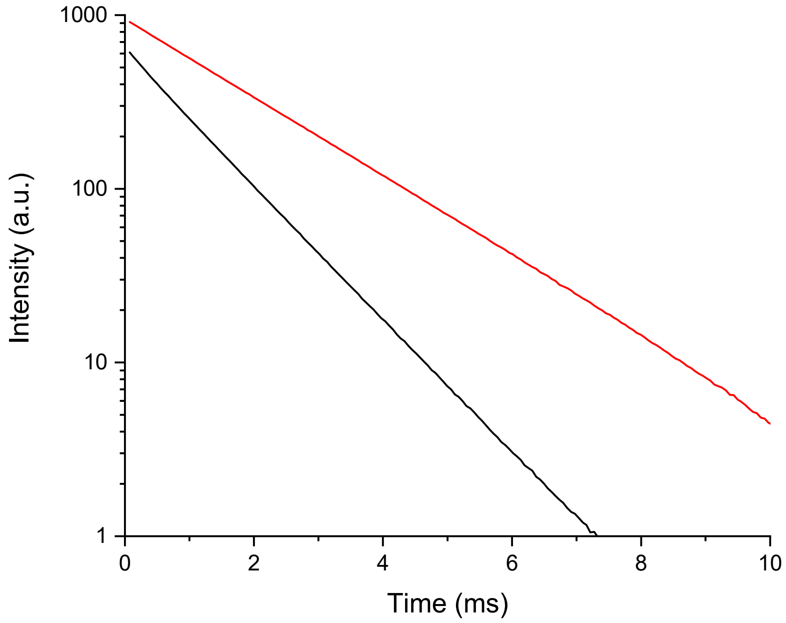

Figure S41b. Decay curves of luminescence of **Eu•5e** in water (black line) and D_2O (red line) at room temperature

43. Figure S42. Luminescence spectrum and decay curves of Eu•4e

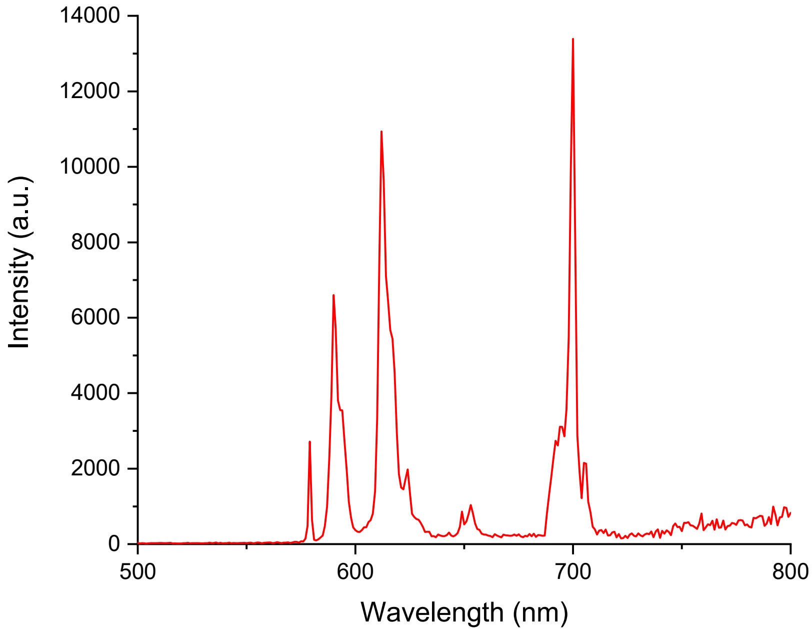

Figure S42a. Europium cation luminescence spectrum of **Eu•4e** in water at room temperature

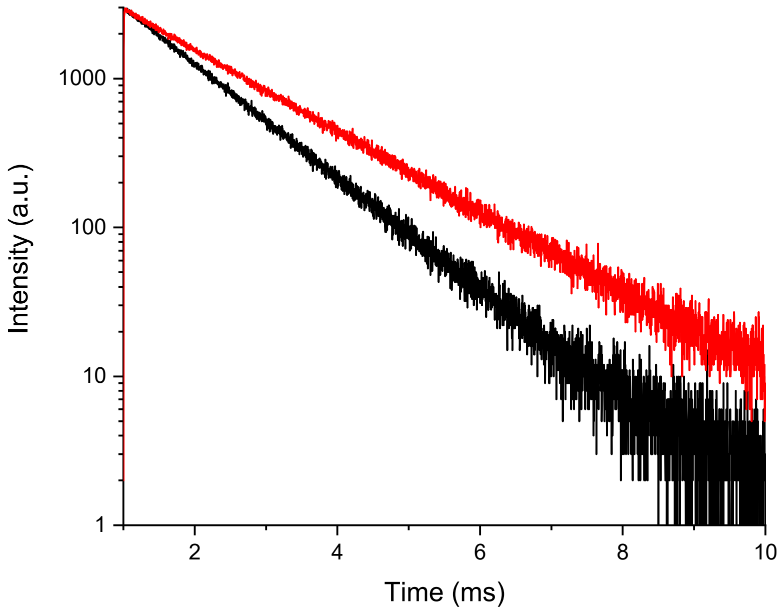

Figure S42b. Decay curves of luminescence of **Eu•4e** in water (black line) and D_2O (red line) at room temperature

44. Figure S43. Luminescence spectrum and decay curves of Eu•4f

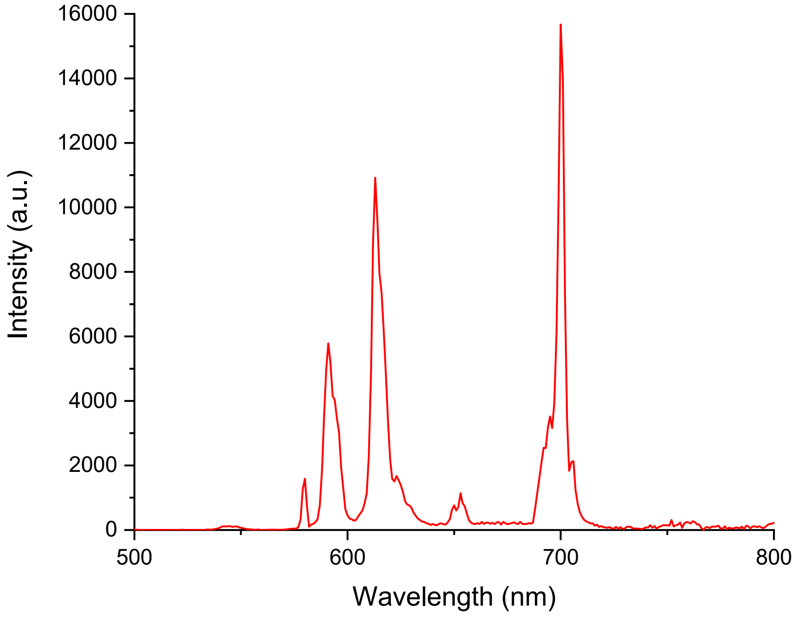

Figure S43a. Europium cation luminescence spectrum of **Eu•4f** in water at room temperature

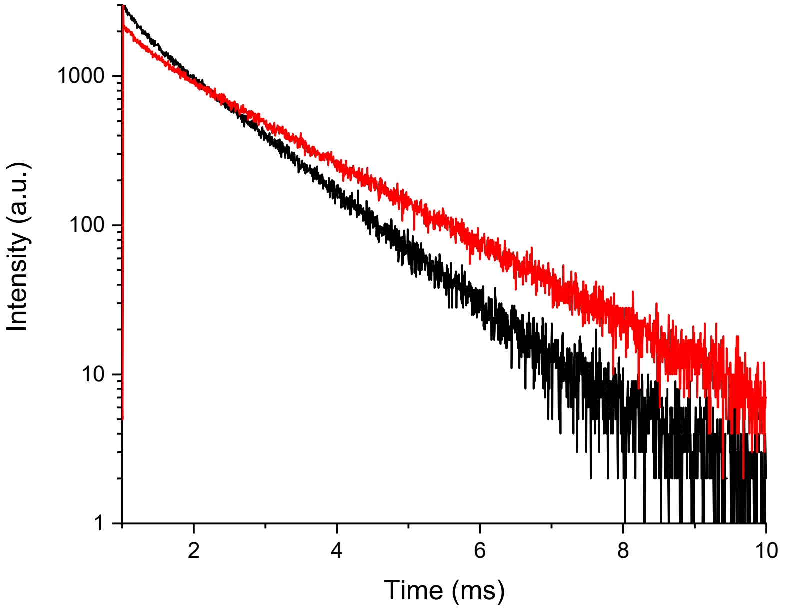

Figure S43b. Decay curves of luminescence of **Eu•4f** in water (black line) and D_2O (red line) at room temperature

45. Figure S44. Luminescence spectrum and decay curves of Tb•4f

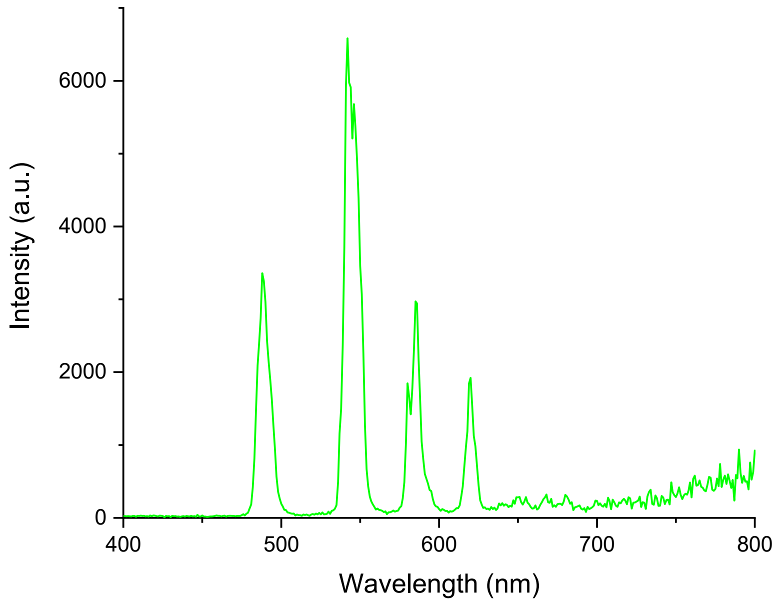

Figure S44a. Terbium cation luminescence spectrum of **Tb•4f** in water at room temperature

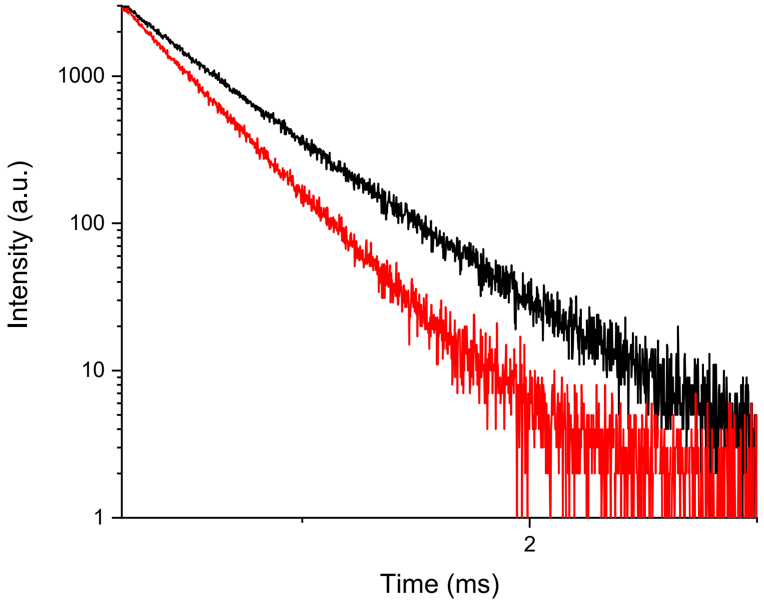

Figure S44b. Decay curves of luminescence of **Tb•4f** in water (black line) and D_2O (red line) at room temperature

46. Figure S45. Luminescence spectrum and decay curves of Eu•4g

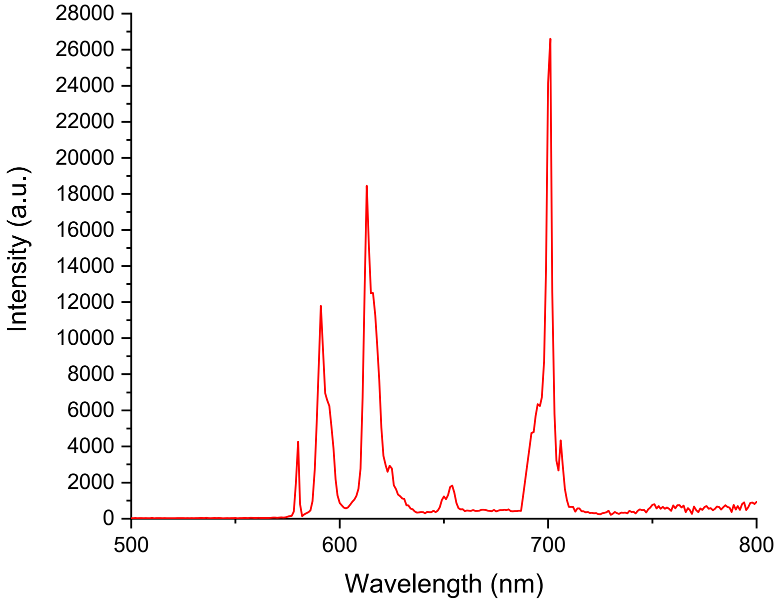

Figure S45a. Europium cation luminescence spectrum of **Eu•4g** in water at room temperature

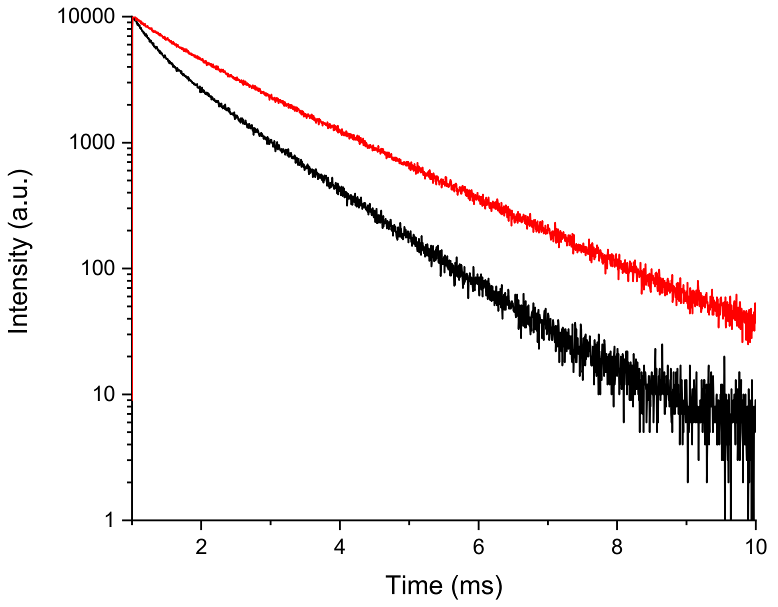

Figure S45b. Decay curves of luminescence of **Eu•4g** in water (black line) and D_2O (red line) at room temperature

47. Figure S46. Luminescence spectrum and decay curve of Tb•4g

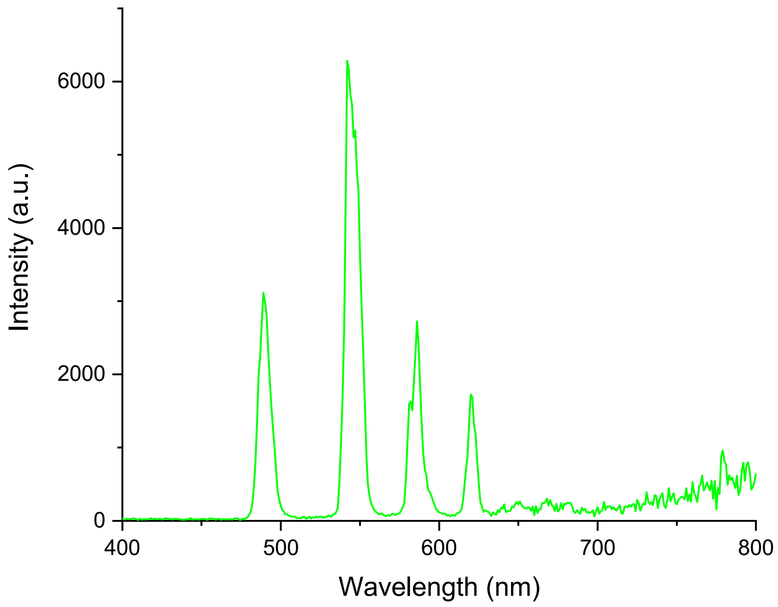

Figure S46a. Terbium cation luminescence spectrum of **Tb•4g** in water at room temperature

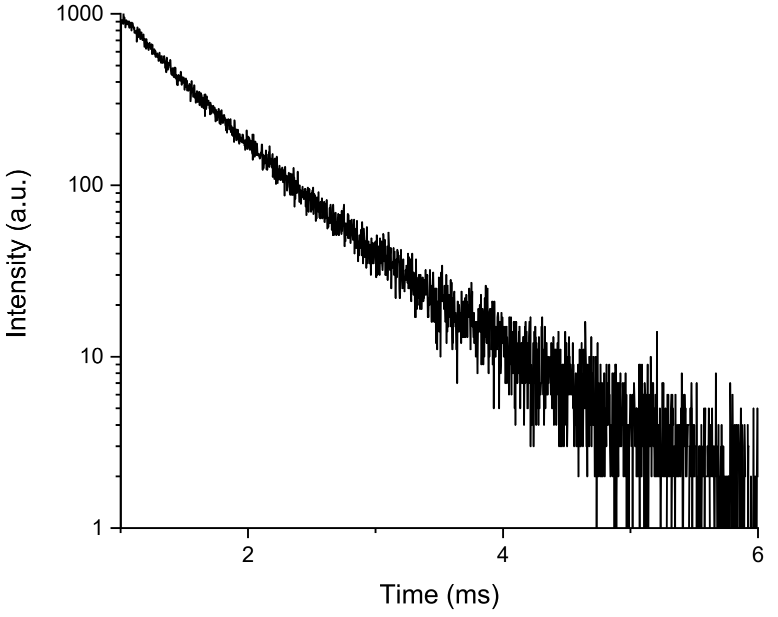

Figure S46b. Decay curve of luminescence of **Tb•4g** in water at room temperature

48. Figure S47. Luminescence spectrum and decay curves of Eu•4h

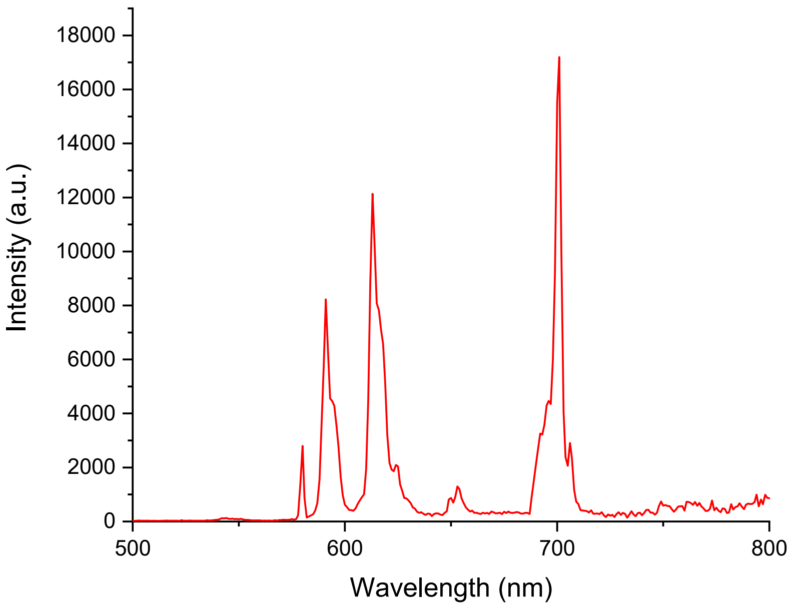

Figure S47a. Europium cation luminescence spectrum of **Eu•4h** in water at room temperature

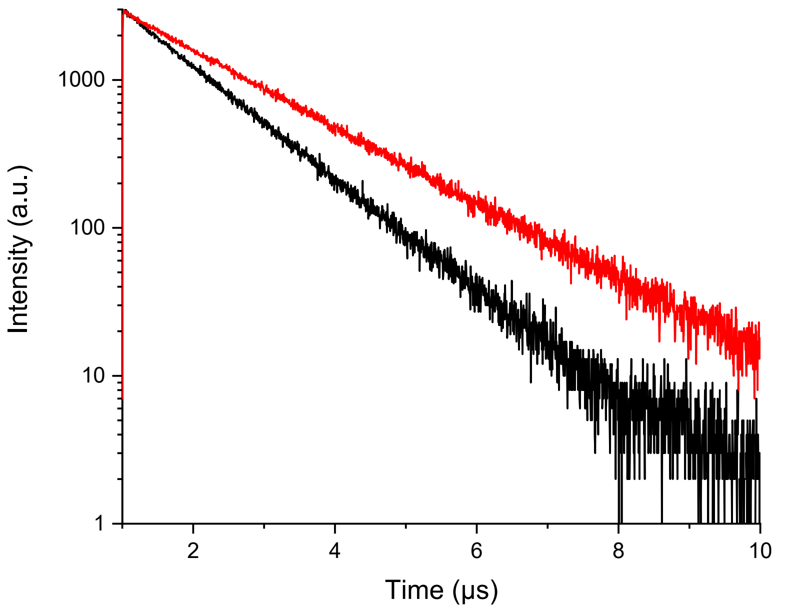

Figure S47b. Decay curves of luminescence of **Eu•4h** in water (black line) and D_2O (red line) at room temperature

49. Figure S48. Luminescence spectrum and decay curves of Tb•4h

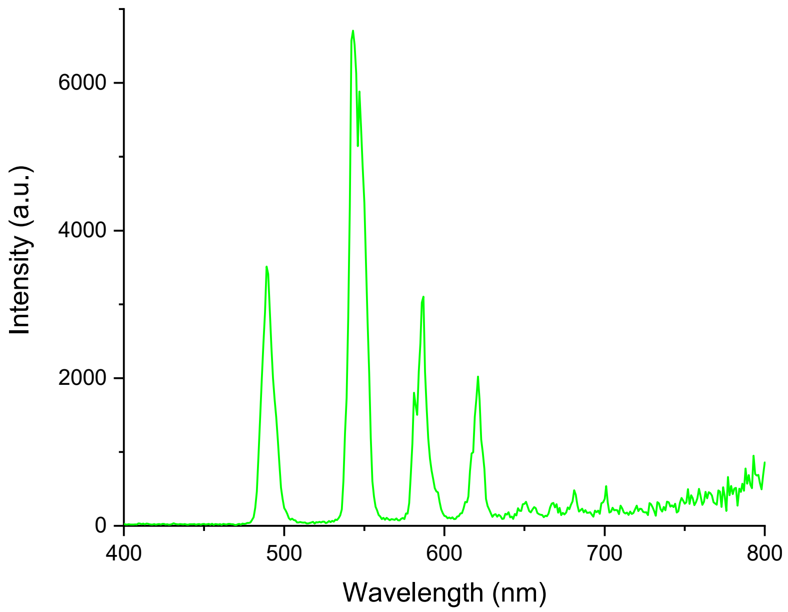

Figure S48a. Terbium cation luminescence spectrum of **Tb•4h** in water at room temperature

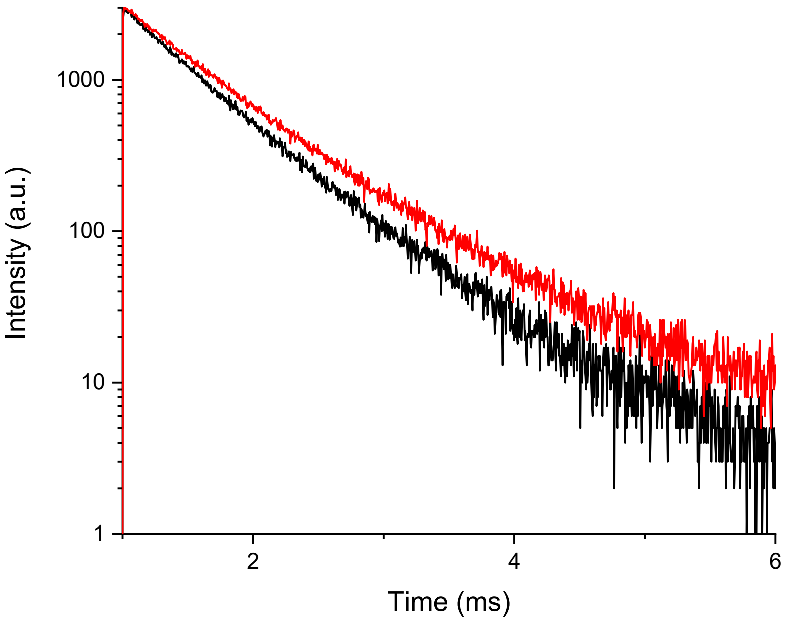

Figure S48b. Decay curves of luminescence of **Tb•4h** in water (black line) and D_2O (red line) at room temperature

50. Figure S49. Luminescence spectrum and decay curves of Eu•4i

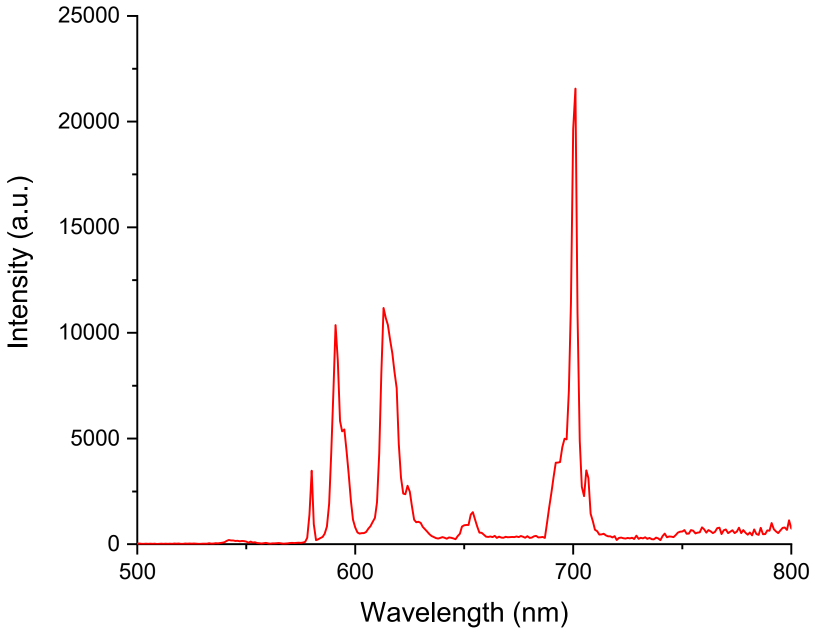

Figure S49a. Europium cation luminescence spectrum of **Eu•4i** in water at room temperature

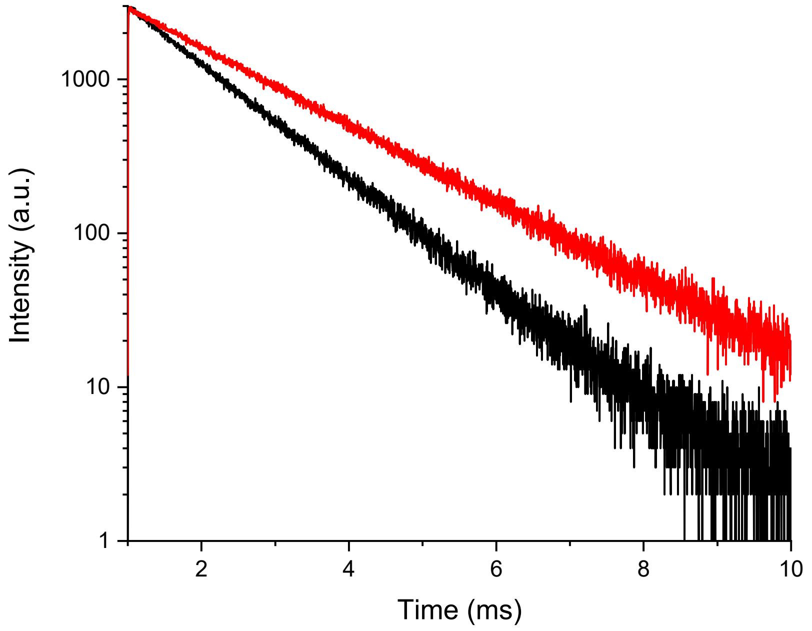

Figure S49b. Decay curves of luminescence of **Eu•4i** in water (black line) and D_2O (red line) at room temperature
